# Scope and limitation of propylene carbonate as a sustainable solvent in the Suzuki–Miyaura reaction†

**DOI:** 10.1039/c9ra07044c

**Published:** 2019-11-20

**Authors:** Andrea Czompa, Balázs László Pásztor, Jennifer Alizadeh Sahar, Zoltán Mucsi, Dóra Bogdán, Krisztina Ludányi, Zoltán Varga, István M. Mándity

**Affiliations:** Department of Organic Chemistry, Faculty of Pharmacy, Semmelweis University Hőgyes Endre u. 7 H-1092 Budapest Hungary mandity.istvan@pharma.semmelweis-univ.hu mandity.istvan@ttk.mta.hu; Femtonics Ltd Tűzoltó u. 59 H-1094 Budapest Hungary; MTA TTK Lendület Artificial Transporter Research Group, Institute of Materials and Environmental Chemistry, Research Center for Natural Sciences, Hungarian Academy of Sciences Magyar Tudósok krt. 2 H-1117 Budapest Hungary; Department of Pharmaceutics, Semmelweis University Hőgyes Endre u. 7 H-1092 Budapest Hungary; Institute of Materials and Environmental Chemistry, Research Center for Natural Sciences, Hungarian Academy of Sciences Magyar Tudósok krt. 2 H-1117 Budapest Hungary

## Abstract

The Suzuki–Miyaura reaction is one of the most used transformations in drug research. Thus making this reaction more sustainable is of considerable current interest. Here we show that propylene carbonate (PC) can be used as a solvent for the Suzuki–Miyaura reaction. PC is one of the greenest solvents since it is synthesized under green conditions by the use of carbon dioxide in the air. All reactions proceeded well and good or excellent yields were observed for the biaryl products. Nonetheless in the case of pyridazinones, 2-hydroxypropyl- chain containing side-products were observed. Importantly, this fact allowed the isolation of several novel compounds which were generated under prominently green conditions.

## Abstract 2

The Suzuki–Miyaura reaction was carried out in propylene carbonate yielding an interesting side-product besides the biphenyl derivative.

## Graphical abstract

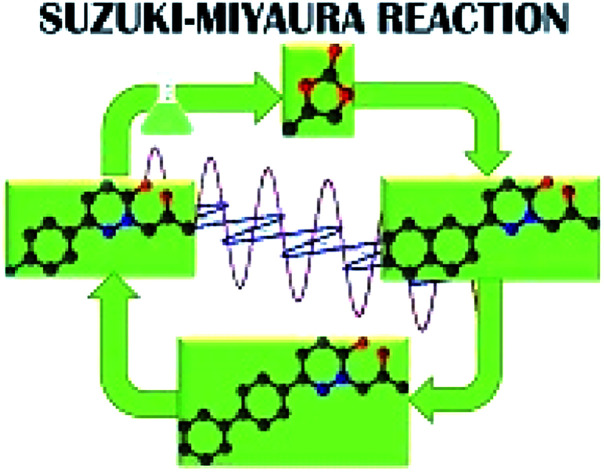

## Introduction

The Suzuki–Miyaura reaction is the palladium-catalysed cross-coupling reaction of organoboranes with organic halides, triflates or perfluorinated sulfonates and proceeds with high stereo- and regioselectivity.^1^ Recent developments with respect to catalysts and methods have broadened the possible applications enormously, that is, the scope of the reaction partners is not restricted to aryls, but includes alkyls, alkenyls and alkynyls too.^2,3^ Usually, the boronic acid is activated by a base and the reaction is run in a polar aprotic solvent, ionic liquid or water.^4^ At room temperature, the reaction takes place with low yield; therefore, the use of special catalyst might be required^5^ or increased temperature and pressure are needed.^6^

Several efforts were taken to carry out Suzuki–Miyaura and further cross coupling reactions under green and sustainable conditions.^7–20^ Reeves and co-workers studied the effects of propylene carbonate (PC) and various polar and nonpolar solvents on palladium-catalyzed Suzuki–Miyaura coupling of chloroaryl triflates. They highlighted the similar selectivity of the solvents and the synthetic value of it.^21^ PC was used with no loss of enantioselectivity in an iridium- and rhodium-phosphite/oxazoline catalytic system catalysed asymmetric hydrogenation of functionalized cyclic β-enamines,^22^ and in cathodic reduction of aryl halides, considered for its high dielectric permittivity.^23^ PC was used in an aminophosphine palladium pincer-catalyzed carbonylative Sonogashira reaction with 10^−4^ mol%, while the Suzuki–Miyaura cross-coupling reaction was carried out at 10^−6^ mol%.^24^ Nonetheless, Pd/C catalysed phenoxycarbonylation of aryl iodide was carried out using *N*-formylsaccharin as a CO surrogate in PC as a sustainable solvent.^25^ Heck reaction was carried in cyclic carbonates as greener solvents, offering effective alternative to traditionally used dipolar aprotic solvents.^26^ Nanostructured palladium clusters catalysed also Heck reaction and were stable in propylene carbonate even at 140–155 °C.^27^

A recent article^28^ reports that nanoparticles formed from inexpensive FeCl_3_ naturally contains parts-per-million (ppm) levels of Pd and can catalyse Suzuki–Miyaura reaction in water. There is also a nickel-catalysed, but base-free Suzuki–Miyaura reaction of carboxylic acid fluorides^29^ and we can found a heterogeneous and ligand-free Suzuki–Miyaura reaction accomplished with the use of a palladium catalyst supported on a double-structure amphiphilic polymer composite.^30^ Interestingly, a heterogeneous single-atom palladium preparation anchored on exfoliated graphitic carbon nitride was used in a homogenous systems for Suzuki coupling.^31^ In 2018, researchers from the Pfizer company published an article^32^ about the use of an automated flow-based synthesis platform, that integrates both rapid nanomole-scale reaction screening and micromole-scale synthesis in Suzuki–Miyaura coupling at elevated temperature. The continuous-flow technology was utilized for several cross coupling reactions too.^11,33–38^

Small and homogenous palladium particules (4–6 nm) were introduced in multi-walled carbon nanotubes used for selective hydrogenation of cinnamaldehyde,^39^ while palladium–nickel and palladium–silver nanoparticles supported on carbon exhibited high activity toward the glycerol electrooxidation in alkaline medium.^40^ Glycerol electrooxidation was achieved also on active self-supported Pd_1_Sn_*x*_ nanoparticles, when the modification of palladium by tin species led to suppress the dissociative adsorption process of glycerol.^41^ de Souza and co-workers published an article describing the resolution of amines.^42^ They immobilized lipase on functionalized Pd–SiO_2_ nanoparticles and this new simplified Pd-lipase hybrid biocatalysts showed immobilization efficiencies of around 80% when containing 1–10% of Pd. Numerous studies focused on the bacterial synthesis of Pd-nanoparticles (bio-Pd NPs) by uptake of Pd(ii) ions and their enzymatically-mediated reduction to Pd(0). One of them deal with microwave-injured Pd(ii)-treated cells (and non MW-treated controls) which were contacted with H_2_ to promote Pd(ii) reduction.^43^

**Scheme 1:**
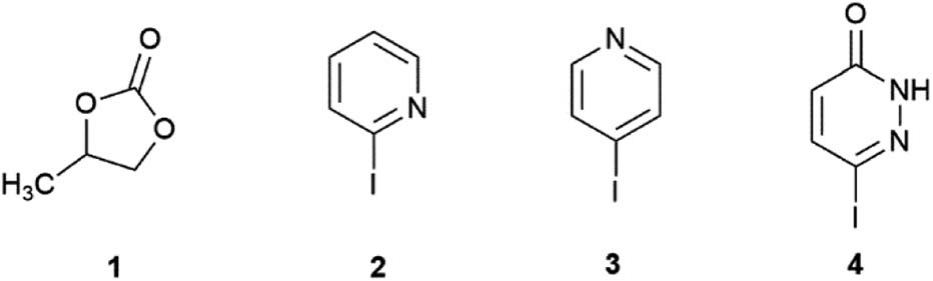
Structure of solvent and substrates.

The Suzuki–Miyaura reaction was investigated under traditional oil-heating and microwave conditions using propylene carbonate (PC, 1,Scheme 1) as a green solvent. Three heterocyclic substrates: 2-iodopyridine (2), 4-iodopyridine (3) and 6-iodopyridazin-3(2*H*)-one (4), were used as coupling partners.

The use of PC allows to increase the temperature of the Suzuki reaction (usually lower or around 100 °C) and meantime, decrease the reaction time. Propylene carbonate (1) is among the green solvents^44^ in the GlaxoSmithKline solvent sustainability guide. It is known as a carbon-dioxide neutral solvent^45^ and can be obtained from propylene oxide and carbon dioxide.^46^ The cycloaddition of CO_2_ and propylene oxide catalysed by NiO-modified TiO_2_ nanoparticles with tetrabutylammonium iodide used as co-catalyst, under solvent free conditions was achieved in excellent yield^47^ and the green synthesis of PC was also published.^48^ In this last paper, the authors used microwave in order to intensify the lipase catalysed transesterification of 1,2-propanediol with dimethyl carbonate in non-aqueous media and observed that microwave irradiation not only increase the reaction rate, but also improved the thermal stability of the enzyme. This “green” solvent is a good electrolyte during lithium-ion intercalation and de-intercalation in lattice contraction/recovery of W_18_O_49_ nanowires used as electrochromic actuator.^49^ The molecular dynamics of lithium and PC in lithium–metal batteries was studied in order to explore the next-generation high-energy lithium–sulfur and lithium–air batteries.^50^ PC can increase optoelectronic properties, photovoltaic parameters and environmental stability of efficient perovskite solar cells against humidity, light and heat.^51^

Herein we show the use of PC as prominent green solvent for the Suzuki–Miyaura reaction. We observed the limitation of PC as solvent, since nucleophiles can open the ring and 2-hydroxypropylation occurs. Nonetheless, this side-reaction allowed us the isolation of several novel compounds, thus, PC can be treated as a prominently green 2-hydroxypropylation reagent.

## Results and discussion

**Scheme 2:**
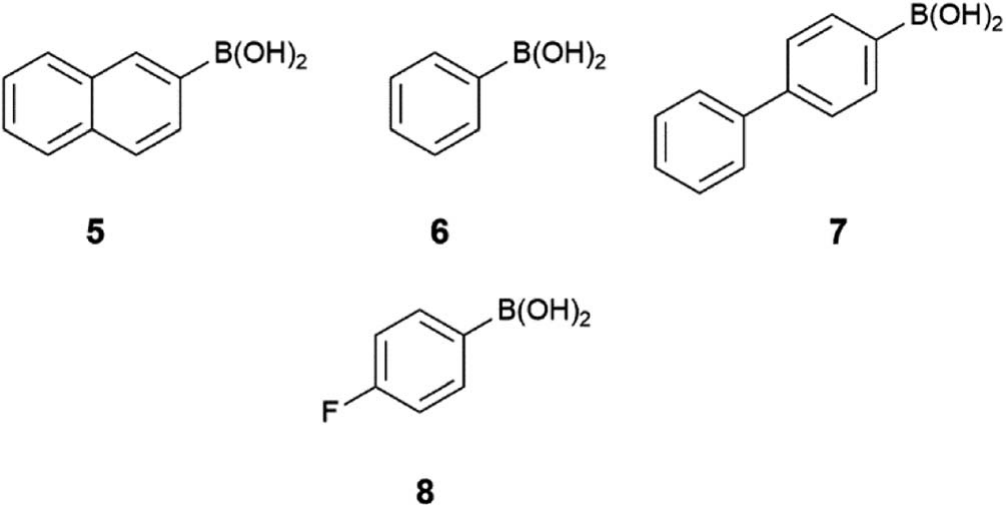
Structure of boronic acids.

As mentioned, two different heating ways (oil bath and MW irradiation) were applied in the Suzuki–Miyaura reaction with 2-iodopyridine (2), 4-iodopyridine (3) and 6-iodopyridazin-3(2*H*)-one (4) as starting materials. The first two are commercial products, while the last one was synthesized in two steps from commercially available 3,6-dichloropyridazine. The chlorine atoms were substituted with iodine^52,53^ in the presence of aq. HI followed by alkaline hydrolysis of the formed 3,6-diiodopyridazine^54,55^ in order to obtain 6-iodopyridazin-3(2*H*)-on (4). The heterocyclic substrates were reacted with four different boronic acids (Scheme 2), namely, 2-naphthylboronic acid (5), phenylboronic acid (6), 4-biphenylboronic acid (7) and 4-fluorophenylboronic acid (8).

**Scheme 3:**
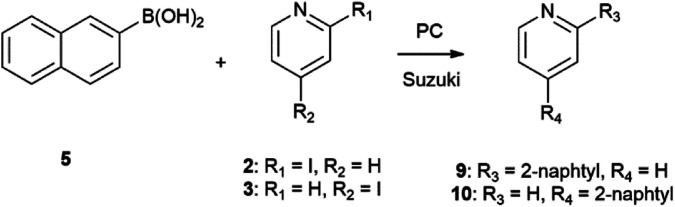
Synthesis of 2- and 4-(naphthalen-2-yl)pyridine.

**Scheme 4:**
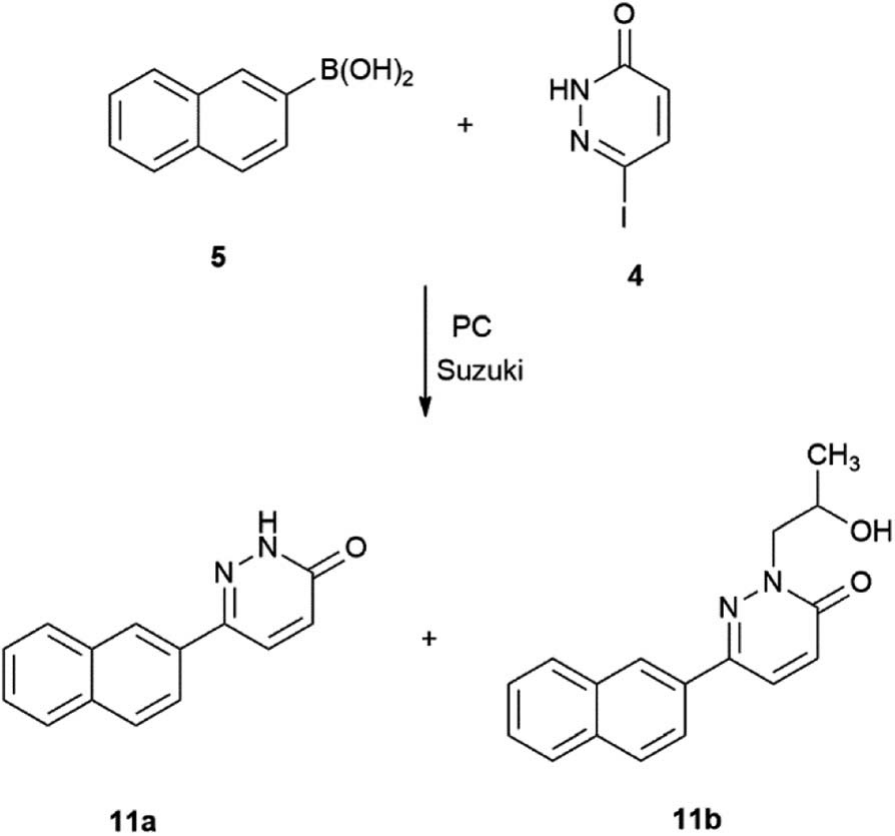
Suzuki–Miyaura reaction of 6-iodopyridazin-3(2*H*)-one with 2-naphthaleneboronic acid.

Reactions were carried out in the presence of tetrakis(triphenylphosphane)palladium(0) as catalyst and disodium carbonate as base. The Suzuki–Miyaura reaction of 5 with iodopyridines is shown on Scheme 3, while with iodopyridazin-3(2*H*)-one 4 is depicted on Scheme 4.

In case of the reaction 5 with 4 a side-product was observed of which is depicted in Scheme 4.

**Scheme 5:**
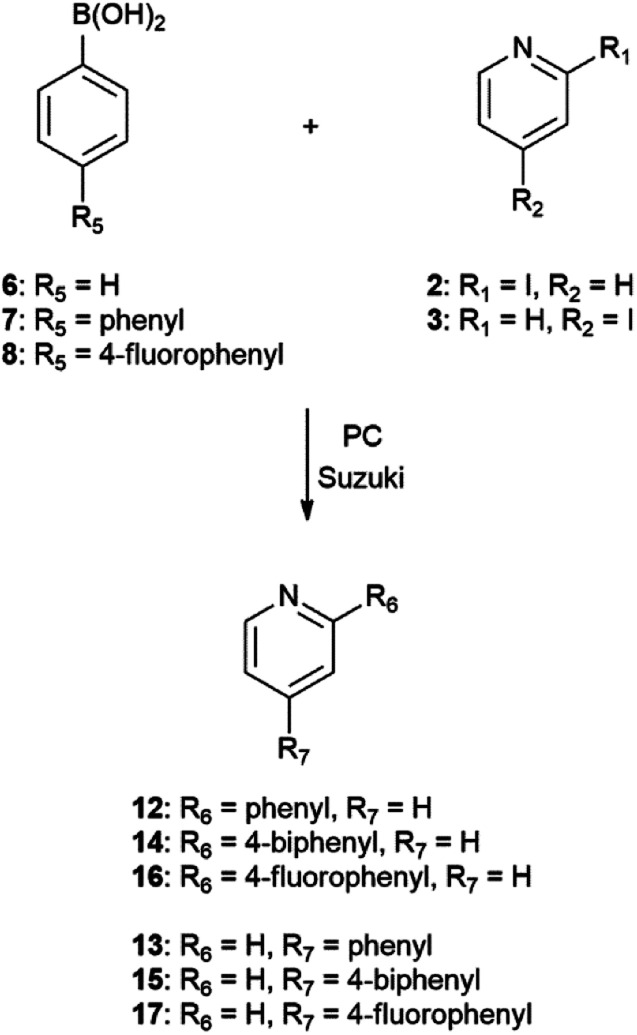
Suzuki–Miyaura reaction of iodopyridines 2 and 3 with phenylboronic acid (6), 4-biphenylboronic acid (7) and 4-fluorobenzeneboronic acid (8).General method: 1 mmol hetrocyclic substrates (2, 3 or 4), 1.25 mmol boronic acids (5, 6, 7 or 8), 0.05 mmol Pd(PPh_3_)_4_, 5 mL PC (1) and 2 mL 0.5 M Na_2_CO_3_ heated at 130 °C.

The Suzuki–Miyaura reaction of phenylboronic acids 6, 7, and 8 with iodopyridines 2 and 3 is shown in Scheme 5.

In case of the Suzuki–Miyaura reaction of iodocompound 4 with phenylboronic acids 6, 7 and 8 again the side-product was formed.

**Scheme 6:**
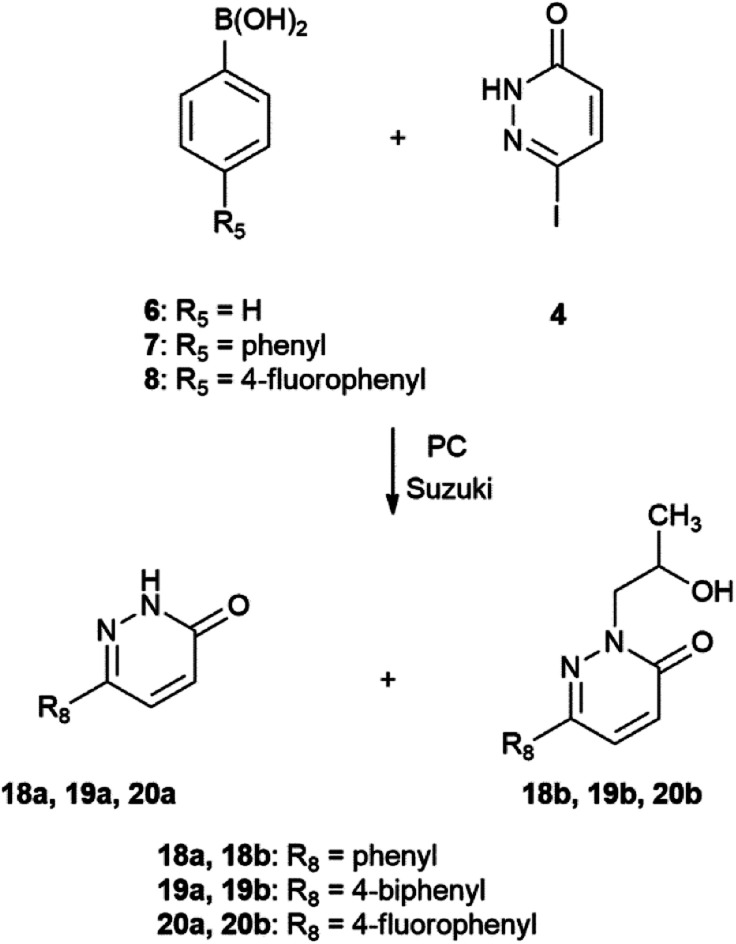
Suzuki–Miyaura reaction of 6-iodopyridazin-3(2*H*)-one (4) with phenylboronic acid (6), 4-biphenylboronic acid (7) and 4-fluorobenzeneboronic acid (8).

By traditional oil-bath heating, reactions were run until full conversion of substrates monitored by TLC at 254 nm. Under microwave irradiation, in turn, in all cases, the reaction time was adjusted to 1 hour, but the same temperature (130 °C) was utilized. The oil-heating experiments of 2-iodopyridine (2) and 4-iodopyridine (3) were performed in the dark (flask covered by tinfoil), because they are light and air sensitive (Schemes 3 and 5). In the case of the third substrate: 6-iodopyridazin-3(2*H*)-one (4), we did not observe stability problems of the starting material, thus there was no need to carry out the reaction in the dark. However, side-products were formed (Schemes 4 and 6; see further discussion).

The Suzuki–Miyaura cross-coupling reactions were carried out under the above-mentioned conditions. On the basis of the necessary reaction times shown in Table 1, we can state that: 4-fluorophenylboronic acid (8) has higher reactivity, while phenylboronic acid (6) is less reactive than all other boronic acids.

**Table 1:** Reaction time (in hours) to attain complete conversion by conventional heating

Reagents	5	6	7	8
2	5 h	7 h	3 h	3 h
3	3 h	3 h	2 h	1 h
4	4 h	6 h	3 h	3 h

Furthermore, 2-naphthylboronic acid (5) and 4-biphenylboronic acid (7) show similar reactivity. The results suggest that the reactivity of aromatic boronic acid is not dramatically influenced by the aromatic substituent. That is the reaction time of compounds 5 and 7 with a fused and isolated aromatic ring, respectively, is very similar. There can be found several data in the literature, such as HPLC of phenylboronic acid^56^ (6) and LC-MS data of 6-phenylpyridazin-3(2*H*)-one^57^ (18a). No information is available, however, for our new products: 11b, 19b and 20b. We measured the maximum absorbance (*λ*_max_) of the starting materials and all products by HPLC-UV method and the retention time of all received products at 254 nm (ESI†).

The results of our experiments are summarized in Table 2. All yields are acceptable or excellent and, in general, the yields of the isolated compounds are in good accordance with those results obtained by HPLC analysis of the raw products.

**Table 2:** Obtained yields for the Suzuki–Miyaura reactions

Yield (%)	5	6	7	8
2	92% (9)^a^	65% (12)^a^	95% (14)^a^	43% (16)^a^
	89% (9)^c^	64% (12)^c^	89% (14)^c^	58% (16)^c^
	93% (9)^b^	65% (12)^b^	97% (14)^b^	50% (16)^b^
	88% (9)^c^	67% (12)^c^	92% (14)^c^	63% (16)^c^
3	85% (10)^a^	47% (13)^a^	92% (15)^a^	71% (17)^a^
	82% (10)^c^	47% (13)^c^	87% (15)^c^	79% (17)^c^
	85% (10)^b^	74% (13)^b^	91% (15)^b^	80% (17)^b^
	86% (10)^c^	69% (13)^c^	84% (15)^c^	84% (17)^c^
4	19% (11a) + 20% (11b)^a^	47% (18a) + 52% (18b)^a^	32% (19a) + 58% (19b)^a^	33% (20a) + 56% (20b)^a^
	21% (11a) + 19% (11b)^c^	40% (18a) + 57% (18b)^c^	21% (19a) + 76% (19b)^c^	43% (20a) + 54% (20b)^c^
	36% (11a) + 52% (11b)^b^	45% (18a) + 45% (18b)^b^	26% (19a) + 49% (19b)^b^	44% (20a) + 50% (20b)^b^
	48% (11a) + 50% (11b)^c^	51% (18a) + 48% (18b)^c^	29% (19a) + 51% (19b)^c^	44% (20a) + 51% (20b)^c^

a Isolated yield for the conventionally heated reaction.

b Isolated yield for microwave assisted reaction.

c Estimated yield from HPLC analysis and weight of the raw products.

According to the necessary reaction times to attain total conversion shown in Table 1, 2-iodopyridine (2) had higher reactivity compared to 4-iodopyridine (3) in the Suzuki–Miyaura reaction with boronic acids 5, 6 and 7. The lowest yield was found in the reaction of 2-iodopyridine derivatives with 4-fluorophenylboronic acid both under conventional heating and under microwave conditions (8). After 3 h reaction time, product 16^58–60^ was formed only in an acceptable average yield of 51% by oil heating and 57% average yield under MW conditions. 4-(4-Fluorophenyl)pyridine^61–63^ (17) was obtained with a 75% average yield by traditional heating in 1 h and 82% average yield under microwave irradiation. From this results we conclude that, in the same reaction time, reactions conducted under microwave irradiation afford higher yields, than those for oil-heating experiments. One explanation can be the inhomogenity of temperature in the oil bath, although the oil was well-stirred with a stirring bar. Product 12^64–66^ can be obtained from 2-iodopyridine (2) and phenylboronic acid (6) in approximately 65% average yield in the longest reaction (7 h, oil bath) and with 66% average yield in 1 h (MW heating). Note that the use of MW irradiation in Suzuki–Miyaura reaction allows us to save time and energy.

4-Iodopyridine 3 in the reaction with boronic acids 5 and 7 gave the corresponding products in very high yields. Product 10^67–69^ was obtained with 84% average yield in 3 h by oil heating, and 86% average yield under MW conditions. Because of its reactivity, 4-biphenylboronic acid (7) gave full conversion under conventional oil heating in only 2 h to afford product 15^70,71^ isolated with 90% average yield. An 88% average isolated yield was observed under MW irradiation.

Both 2-(naphthalen-2-yl)pyridine^72,73^ (9) and 2-(biphenyl-4-yl)pyridine^74,75^ (14) could be obtained in excellent average yields in oil bath: 91% in 5 h (9) and 92% in 3 h (14). Similar tendency can be observed for reactions conducted under microwave irradiation, but in shorter reactions of 1 h: 91% for 9 and 95% for 14. Interestingly, compound 13^76–78^ was obtained in the lowest yield of 47% by oil heating; however, this increased to an average yield of 72% under MW conditions.

In the Suzuki–Miyaura reaction of 6-iodopyridazin-3(2*H*)-one (4), beside the expected products (11a, 18a, 19a and 20a) four other compounds (11b, 18b, 19b and 20b) were also formed. Furthermore, these side-products seem to be formed in 1 : 1 proportion with the expected ones, like in the reaction of 4 with 5 under conventionally heating or with 6 under microwave irradiation (see related data in Table 2). Compounds 11a,^79,80^18a^79,81,82^ and 18b^83,84^ are known in the literature; however, side-product 11b is not yet published. As shown, it was obtained in a yield similar to those of the expected product 11a. The same is true for products 18a and 18b.

In contrast, in the reaction of 7 and 8, the unexpected products (19b, 20b) seem to be formed in higher yields than those for the expected one (19a, 20a). We observed a 27% average yield for 19a^85^ and a 59% average yield both under oil bath and MW heating conditions for 19b. A similar tendency was observed in the reaction of 4 with 4-fluorophenylboronic acid (8). Product 20b was formed in an average yield of 53%, while only a 41% yield was measured for 20a^86–88^ both under microwave conditions and oil-bath heating. The structure of the side-products was elucidated by NMR spectroscopy and mass spectroscopy (ESI†).

**Scheme 7:**
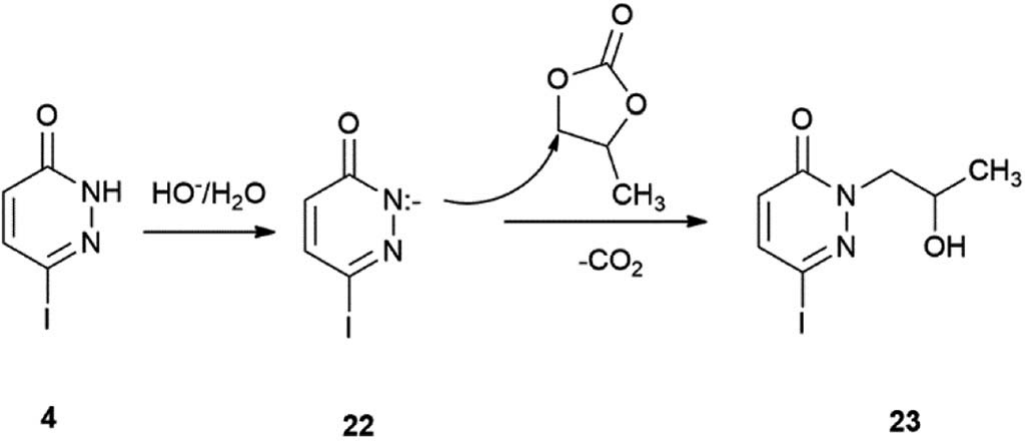
Reaction of PC with 6-iodopyridazin-3(2*H*)-one under alkaline conditions.

The explanation for the formation of byproducts 11b, 18b, 19b and 20b is the base-induced deprotonation of the pyridazinone and opening of propylene carbonate^89^ ring (Scheme 7). The hydrolysis of propylene carbonate to 1,2-propylene glycol was published by the use of a supported basic ionic liquid in a stainless steel autoclave heated to 140 °C for 4 h.^90^

We did not observe the formation of side-products in the reaction of 2-iodopyridine (2) and 4-iodopyridine (3), because they do not have acidic H attached to the nitrogen atom. The hydrogen on the *N*-2 atom of 6-iodopyridazin-3(2*H*)-one (4) is acidic (Scheme 7); therefore, it can easily undergo deprotonation by aqueous Na_2_CO_3_ and the formed negatively charged nucleophilic *N*-atom of the pyridazinone ring (22) will attack the most electrophilic C-atom of propylene carbonate (1). Subsequent ring opening results in the formation of 2-(2-hydroxypropyl)-6-iodopyridazin-3(2*H*)-one (23), which will react with the corresponding boronic acid derivatives and give the side-compounds. Although, Allerton and co-workers published the alkylation of the *N*-2 atom of the 6-piperazinyl substituted pyridazinone ring, but only with a side chain formed from ethylene carbonate, in DMF-solution and KOH as base. Similarly, the *N*-alkylation of the benzyl 4-(6-oxo-1,6-dihydropyridazin-3-yl)piperazin-1-carboxylate with (*R*)- and (*S*)-propylene oxide was carried out under phase transfer conditions in DCM and water, respectively.^91^

**Scheme 8:**
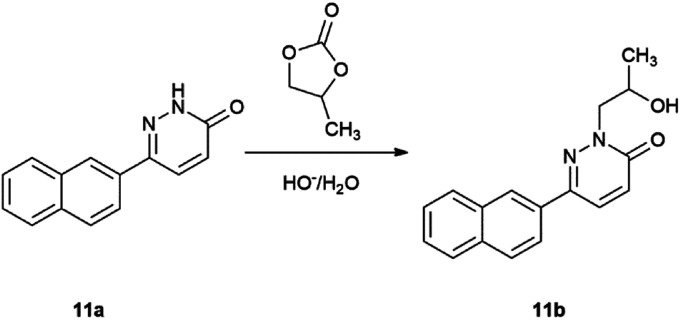
Reaction of PC with the product of Suzuki–Miyaura reaction.

In order to support our theory, we made additional experiments. Starting from pyridazinone (4) we synthesized compound (23) under 1 h MW irradiation (Scheme 7), in the presence of the solvent (PC), base (Na_2_CO_3_), without and with Pd(PPh_3_)_4_ as catalyst. Compound 23, an unknown product, was obtained in good yields of 80% and 78%. On the other hand, we performed a reaction starting from 11a, only in the presence of propylene carbonate (1) and the base and obtained product 11b in 67% yield (Scheme 8).

This experimental observation suggests that under similar reaction conditions (1 h MW irradiation or 130 °C, oil bath heating) more side-product 11b can be obtained rather than the expected Suzuki-coupled product (11a) and we propose that compound 23 is an intermediate in all Suzuki–Miyaura reactions starting from heterocyclic substrate 4. Additionally, not only 23 can lead to the formation of 11b, however the cross-coupled 11a can react with PC yielding 11b.

**Fig. 1:**
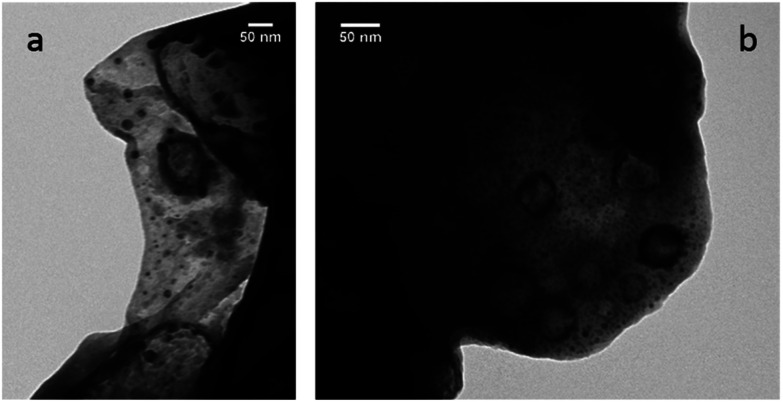
Pd nanoparticles in the 1–5 nm diameter range found in the reaction mixture of the synthesis of 17 after 40 (a) and 60 (b) min reaction time.

It was proposed that Pd nanoparticles are effective catalysts for Suzuki–Miyaura reaction.^92^ To test the presence of nanoparticles in our reactions carried out in PC, TEM measurements were carried out. The synthesis of 17 was used as test reactions, and aliquots were taken at 20, 40 and 60 min. After an hour the reaction is complete. The aliquots were lyophilized, the resulted solid was dissolved in methanol, and this solution was investigated by TEM measurements. The results indicate that nanoparticles in the diameter range of 1–5 nm can be observed in the sample at 40 and 60 min (Fig. 1).

**Scheme 9:**
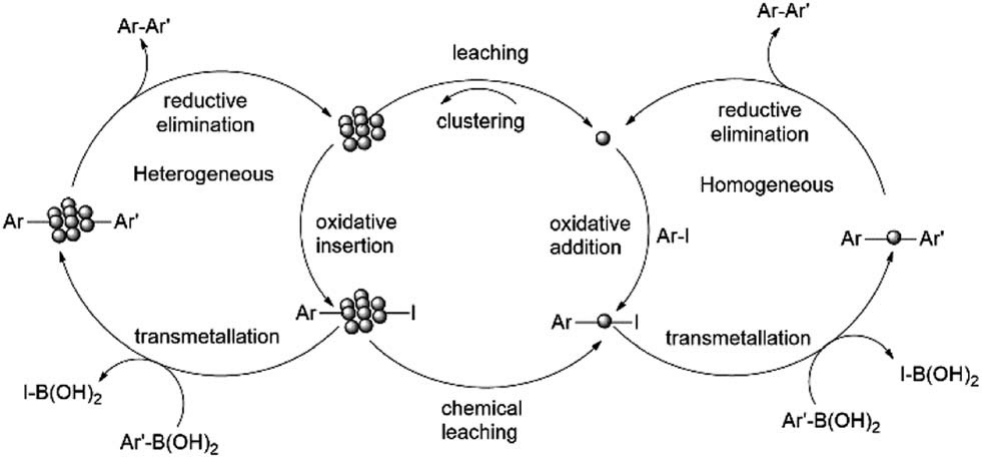
Reaction mechanism showing nanoparticle formation and Suzuki-product synthesis both in homogeneous and heterogeneous phase.^92^

The mechanism depicting the nanoparticle formation and the Suzuki-product synthesis both in homogeneous and heterogeneous phase is shown in Scheme 9.

## Conclusions

PC was used as prominently green solvent for the Suzuki–Miyaura cross coupling reaction. The reaction was examined with 3 different heterocyclic iodocompounds (2, 3, 4) with 4 different boronic acids (5, 6, 7, 8) under microwave condition and conventional oil bath heating. In general, iodopyridines 2 and 3 reacted smoothly in the expected way providing the cross-coupled product. However, 6-iodopyridazin-3(2*H*)-on (4) reacted alternatively and side-product formation was observed. Nevertheless, 6-iodopyridazin-3(2*H*)-on (4) is the most reactive with phenyl- (6) and 4-fluorophenylboronic acid (8) (containing a single aromatic ring), while 2-iodopyridine (2) gave the best yield with 2-naphthylboronic acid (5), and 4-biphenylboronic acid (7) containing two aromatic rings. 4-Iodopyridine (3) provided higher yields with boronic acids 5 and 7, and also afforded good yield with 4-fluorophenylboronic acid 8.

Despite of its high boiling point, solvent PC was found to be rather reactive under our reaction conditions. This lead to the formation of side-products in case of iodopyridazin-3(2*H*)-on. However, as a result of the degradation of PC, we were able to synthesize new heterocyclic compounds possessing 2-hydroxypropyl substituent and 11b, 19b, 20b and 23 were isolated in good yields. Besides of the isolated novel compounds, we demonstrated that PC can be an efficient and cost-effective solvent to perform “green” Suzuki reactions by traditional oil and MW heating. Importantly, considerable attention should be payed for the substrate selection, since nucleophiles can open the ring of PC yielding side-products with 2-hydroxypropyl side-chain.

## Conflicts of interest

There are no conflicts to declare.

## Supplementary Material

RA-009-C9RA07044C-s001
