# Supplementary material for: Scope and limitation of propylene carbonate as a sustainable solvent in the Suzuki–Miyaura reaction
Source: RSC Adv. 2019 Nov 20;9(65):37818–24. doi: 10.1039/c9ra07044c (PMC9075783; doi:10.1039/c9ra07044c)
Supplement: RA-009-C9RA07044C-s001 [file RA-009-C9RA07044C-s001.pdf]

Electronic Supplementary Material (ESI) for RSC Advances.
This journal is © The Royal Society of Chemistry 2019

Scope and limitation of propylene carbonate as a sustainable solvent in Suzuki–Miyaura reaction

Andrea Czompa, [a] Balázs László Pásztor, [a] Jennifer Alizadeh Sahar, [a] Zoltán Mucsi, [b] Dóra Bogdán, [a,c] Krisztina Ludányi, [d] Zoltán Varga, [e] and István M. Mándity *[a,c]

[a]Department of Organic Chemistry, Faculty of Pharmacy, Semmelweis University, H-1092 Budapest Hőgyes Endre u. 7 Hungary.

[b]Femtonics Ltd, H-1094 Budapest, Hungary.

[c]MTA TTK Lendület Artificial Transporter Research Group, Institute of Materials and Environmental Chemistry, Research Center for Natural Sciences, Hungarian Academy of Sciences, H-1117 Budapest Magyar Tudósok krt. 2. Hungary.

[d]Department of Pharmaceutics, Semmelweis University, H-1092 Budapest Hőgyes Endre u. 7 Hungary.

[e]Institute of Materials and Environmental Chemistry, Research Center for Natural Sciences, Hungarian Academy of Sciences, H-1117 Budapest Magyar Tudósok krt. 2. Hungary.

TOC

Experimental section S8

General procedures S8

Method A S8

Suzuki cross-coupling reaction of iodopyridine isomers (Figure 3 and Figure 5) S8

Suzuki cross-coupling reaction of 6-iodopyridazin-3(2*H*)-one (Figure 4 and Figure 6) S8

Method B: Reactions under MW conditions S9

NMR measurements S9

HPLC-UV method for measuring max. absorbance of the compounds S9

Table S1. Gradient elution in HPLC-UV method S10

Table S2: Measured maximum absorbance by HPLC-UV method. S10

HPLC-MS method for the characterization of products purity S10

Table S3. Gradient elution in HPLC-MS method S10

Table S4: Retention time measured on 254 nm. S10

3,6-diiodopyridazine: S11

6-iodopyridazine-3(2H)-one (4): S11

2-(naphthalen-2-yl)pyridine (9): S11

4-(naphthalen-2-yl)pyridine (10): S11

6-(naphthalen-2-yl)pyridazin-3(2*H*)-one (11a) and 2-(2-hydroxypropyl)-6-(naphthalen-2-yl)pyridazin-3(2*H*)-one (11b): S12

2-phenylpyridine (12): S12

4-phenylpyridine (13): S13

2-(biphenyl-4-yl)pyridine (14): S13

4-(biphenyl-4-yl)pyridine (15): S14

2-(4-fluorophenyl)pyridine (16): S14

4-(4-fluorophenyl)pyridine (17): S14

6-phenylpyridazin-3(2*H*)-one (18a) and 2-(2-hydroxypropyl)-6-phenylpyridazin-3(2*H*)-one (18b): S15

6-(biphenyl-4-yl)pyridazin-3(2*H*)-one (19a) and 6-(biphenyl-4-yl)-2-(2-hydroxypropyl)-pyridazin-3(2*H*)-one (19b): S15

6-(4-fluorophenyl)pyridazin-3(2*H*)-one (20a) and 6-(4-fluorophenyl)-2-(2-hydroxypropyl)pyridazin-3(2*H*)-one (20b): S16

2-(2-hydroxypropyl)-6-iodopyridazin-3(2*H*)-one (23) obtained without and with catalyst: S17

2-(2-hydroxypropyl)-6-(naphthalen-2-yl)pyridazin-3(2*H*)-one (11b) from 6-(naphthalen-2-yl)pyridazin-3(2*H*)-one (11a):..S17

Fig. S1: UV spectrum of 2-iodopyridine (2)..S18

Fig. S2: UV spectrum of 4-iodopyridine (3)..S18

Fig. S3: UV spectrum of 6-iodopyridazin-3(2*H*)-one (4)..S18

Fig. S4: UV spectrum of 2-naphthaleneboronic acid (5)..S19

Fig. S5: UV spectrum of phenylboronic acid (6)..S19

Fig. S6: UV spectrum of 4-biphenylboronic acid (7)..S19

Fig. S7: UV spectrum of 4-fluorophenylboronic acid (8)..S20

Fig. S8: UV spectrum of 2-(naphthalen-2-yl)pyridine (9)...S20

Fig. S9: UV spectrum of 4-(naphthalen-2-yl)pyridine (10)...S20

Fig. S10: UV spectrum of 6-(naphthalen-2-yl)pyridazin-3(2*H*)-one (11a)...........................S21

Fig. S11: UV spectrum of 2-(2-hydroxypropyl)-6-(naphthalen-2-yl)pyridazin-3(2*H*)-one (11b)...S21

Fig. S12: UV spectrum of 2-phenylpyridine (12)..S22

Fig. S13: UV spectrum of 4-phenylpyridine (13)..S22

Fig. S14: UV spectrum of 2-(biphenyl-4-yl)pyridine (14)..S22

Fig. S15: UV spectrum of 4-(biphenyl-4-yl)pyridine (15)..S23

Fig. S16: UV spectrum of 2-(4-fluorophenyl)pyridine (16)..S23

Fig. S17: UV spectrum of 4-(4-fluorophenyl)pyridine (17)..S23

Fig. S18: UV spectrum of 6-phenylpyridazin-3(2*H*)-one (18a)...S24

Fig. S19: UV spectrum of 2-(2-hydroxypropyl)-6-phenylpyridazin-3(2*H*)-one (18b)..................S24

Fig. S20: UV spectrum of 6-(biphenyl-4-yl)pyridazin-3(2*H*)-one (19a)...............................S24

Fig. S21: UV spectrum of 6-(biphenyl-4-yl)-2-(2-hydroxypropyl)-pyridazin-3(2*H*)-one (19b)......S25

Fig. S22: UV spectrum of 6-(4-fluorophenyl)pyridazin-3(2*H*)-one (20a)..............................S25

Fig. S23: 6-(4-fluorophenyl)-2-(2-hydroxypropyl)pyridazin-3(2*H*)-one (20b)............................S25

Fig. S24: 2-(2-hydroxypropyl)-6-iodopyridazin-3(2*H*)-one (23)..S26

Fig. S25. HPLC-MS data for 3,6-diiodopyridazine:..S26

Fig. S26. HPLC-MS data for 6-iodopyridazine-3(2*H*)-one (4):...S27

Fig. S27. HPLC-MS data for purified 2-(naphthalen-2-yl)pyridine (9):..................................S27

Fig S28. HPLC-MS data for 2-(naphthalen-2-yl)pyridine (9) by Method A:.............................S28

Fig. S29. HPLC-MS data for 2-(naphthalen-2-yl)pyridine (9) by Method B:............................S28

Fig. S30. HPLC-MS data for purified 4-(naphthalen-2-yl)pyridine (10):..................................S29

Fig. S31. HPLC-MS data for 4-(naphthalen-2-yl)pyridine (10) by Method A:...........................S29

Fig. S32. HPLC-MS data for 4-(naphthalen-2-yl)pyridine (10) by Method B:...........................S30

Fig. S33. HPLC-MS data for purified 6-(naphthalen-2-yl)pyridazin-3(2*H*)-one (11a): S30

Fig. S34. HPLC-MS data for 6-(naphthalen-2-yl)pyridazin-3(2*H*)-one (11a) S31

by Method A: S31

Fig. S35. HPLC-MS data for 6-(naphthalen-2-yl)pyridazin-3(2*H*)-one (11a) S32

by Method B: S32

Fig. S36. HPLC-MS data for purified 2-(2-hydroxypropyl)-6-(naphthalen-2-yl)pyri-dazin-3(2*H*)-one (11b): S32

Fig. S37. HPLC-MS data for 2-(2-hydroxypropyl)-6-(naphthalen-2-yl)pyridazin-3(2*H*)-one (11b) by Method A: S33

Fig. S38. HPLC-MS data for 2-(2-hydroxypropyl)-6-(naphthalen-2-yl)pyridazin-3(2*H*)-one (11b) by Method B: S34

Fig. S39. HPLC-MS data for 6-(naphthalen-2-yl)pyridazin-3(2*H*)-one (11a) and 2-(2-hydroxypropyl)-6-(naphthalen-2-yl)pyridazin-3(2*H*)-one (11b) by Method B: S35

Fig. S40. HPLC-MS data for purified 2-phenylpyridine (12): S36

Fig. S41. HPLC-MS data for 2-phenylpyridine (12) by Method A: S36

Fig. S42. HPLC-MS data for purified 2-phenylpyridine (12): S37

Fig. S43. HPLC-MS data for 2-phenylpyridine (12) by Method B: S37

Fig. S44. HPLC-MS data for purified 4-phenylpyridine (13): S38

Fig. S45. HPLC-MS data for 4-phenylpyridine (13) by Method A: S39

Fig. S46. HPLC-MS data for 4-phenylpyridine (13) by Method B: S40

Fig. S47. HPLC-MS data for 4-phenylpyridine (13) by Method B: S40

Fig. S48. HPLC-MS data for purified 2-(biphenyl-4-yl)pyridine (14):...S41

Fig. S49. HPLC-MS data for 2-(biphenyl-4-yl)pyridine (14) by Method A: S41

Fig. S50. HPLC-MS data for 2-(biphenyl-4-yl)pyridine (14) by Method B:..................................S42

Fig. S51. HPLC-MS data for 2-(biphenyl-4-yl)pyridine (14) by Method B:..................................S43

Fig. S52. HPLC-MS data for purified 4-(biphenyl-4-yl)pyridine (15):...S44

Fig. S53. HPLC-MS data for 4-(biphenyl-4-yl)pyridine (15) by Method A: S44

Fig. S54. HPLC-MS data for 4-(biphenyl-4-yl)pyridine (15) by Method B:..................................S45

Fig. S55. HPLC-MS data for 4-(biphenyl-4-yl)pyridine (15) by Method B:..................................S46

Fig. S56. HPLC-MS data for purified 2-(4-fluorophenyl)pyridine (16): S47

Fig. S57. HPLC-MS data for 2-(4-fluorophenyl)pyridine (16) by Method A:................................S47

Fig. S58. HPLC-MS data for 2-(4-fluorophenyl)pyridine (16) by Method B:................................S48

Fig. S59. HPLC-MS data for 2-(4-fluorophenyl)pyridine (16) by Method B:................................S48

Fig. S60. HPLC-MS data for purified 4-(4-fluorophenyl)pyridine (17): S49

Fig. S61. HPLC-MS data for 4-(4-fluorophenyl)pyridine (17) by Method A:................................S50

Fig. S62. HPLC-MS data for 4-(4-fluorophenyl)pyridine (17) by Method B:................................S50

Fig. S63. HPLC-MS data for 4-(4-fluorophenyl)pyridine (17) by Method B:................................S51

Fig. S64. HPLC-MS data for purified 6-phenylpyridazin-3(2*H*)-one (18a): S52

Fig. S65. HPLC-MS data for 6-phenylpyridazin-3(2*H*)-one (18a) by Method A S52

Fig. S66. HPLC-MS data for 6-phenylpyridazin-3(2*H*)-one (18a) and 2-(2-hydroxypropyl)-6-phenylpyridazin-3(2*H*)-one (18b) by Method A:..S53

Fig. S67. HPLC-MS data for purified 2-(2-hydroxypropyl)-6-phenylpyridazin-3(2H)-one (18b):..S54

Fig. S68. HPLC-MS data for 2-(2-hydroxypropyl)-6-phenylpyridazin-3(2*H*)-one (18b) S54

by Method B: S54

Fig. S69. HPLC-MS data for 6-phenylpyridazin-3(2*H*)-one (18a) and 2-(2-hydroxypropyl)-6-phenylpyridazin-3(2*H*)-one (18b) by Method B:..S55

Fig. S70. HPLC-MS data for purified 6-(biphenyl-4-yl)pyridazin-3(2*H*)-one (19a):......................S56

Fig. S71. HPLC-MS data for 6-(biphenyl-4-yl)pyridazin-3(2*H*)-one (19a) by Method A:..............S56

Fig. S72. HPLC-MS data for 6-(biphenyl-4-yl)pyridazin-3(2*H*)-one (19a) and 6-(biphenyl-4-yl)-2-(2-hydroxypropyl)-pyridazin-3(2*H*)-one (19b) by Method A:..S57

Fig. S73. HPLC-MS data for purified 6-(biphenyl-4-yl)-2-(2-hydroxypropyl)pyridazin-3(2*H*)-one (19b): S58

Fig. S74. HPLC-MS data for 6-(biphenyl-4-yl)-2-(2-hydroxypropyl)pyridazin-3(2*H*)-one (19b) by Method B:..S59

Fig. S75. HPLC-MS data for 6-(biphenyl-4-yl)pyridazin-3(2*H*)-one (19a) and 6-(biphenyl-4-yl)-2-(2-hydroxypropyl)-pyridazin-3(2*H*)-one (19b) by Method B: S60

Fig. S76. HPLC-MS data for purified 6-(4-fluorophenyl)pyridazin-3(2*H*)-one (20a): S61

Fig. S77. HPLC-MS data for purified 6-(4-fluorophenyl)-2-(2-hydroxypropyl)pyridazin-3(2*H*)-one (20b): S62

Fig. S78. HPLC-MS data for 6-(4-fluorophenyl)pyridazin-3(2*H*)-one (20a) and 6-(4-fluorophenyl)-2-(2-hydroxypro-pyl)pyridazin-3(2*H*)-one (20b) by Method A: S63

Fig. S79. HPLC-MS data for 6-(4-fluorophenyl)-2-(2-hydroxypropyl)pyridazin-3(2*H*)-one (20b) by Method B:..S63

Fig. S80. HPLC-MS data for 6-(4-fluorophenyl)pyridazin-3(2*H*)-one (20a) and 6-(4-fluorophenyl)-2-(2-hydroxypro-pyl)pyridazin-3(2*H*)-one (20b) by Method B: S64

Fig. S81. HPLC-MS data for purified 2-(2-hydroxypropyl)-6-iodopyridazin-3(2*H*)-one (23):........S65

Fig. S82. HPLC-MS data for purified 2-(2-hydroxypropyl)-6-(naphthalene-2-yl)pyridazin-3(2*H*)-one (11b) obtained from 6-(naphthalene-2-yl)pyridazin-3(2*H*)-one (11a):...............................S65

Fig. S83. ^{1}H NMR spectrum of 3,6-diiodopyridazine:...S66

Fig. S84. ^{13}C NMR spectrum of 3,6-diiodopyridazine:..S66

Fig. S85. ^{1}H NMR spectrum of (4): 6-iodopyridazin-3(2*H*)-one..S67

Fig. S86. ^{13}C NMR spectrum of (4): 6-iodopyridazin-3(2*H*)-one S67

Fig. S87. ^{1}H NMR spectrum of (9): 2-(naphthalen-2-yl)pyridine S68
Fig. S88. ^{13}C NMR spectrum of (9): 2-(naphthalen-2-yl)pyridine S68
Fig. S89. ^{1}H NMR spectrum of (10): 4-(naphthalen-2-yl)pyridine S69
Fig. S90. ^{13}C NMR spectrum of (10): 4-(naphthalen-2-yl)pyridine S69
Fig. S91. ^{1}H NMR spectrum of (11a): 6-(naphthalen-2-yl)pyridazin-3(2*H*)-one S70
Fig. S92. ^{13}C NMR spectrum of (11a): 6-(naphthalen-2-yl)pyridazin-3(2*H*)-one S70
Fig. S93. ^{1}H NMR spectrum of (11b): 2-(2-hydroxypropyl)-6-(naphthalen-2-yl)pyri-dazin-3(2*H*)-one S71
Fig. S94. ^{13}C NMR spectrum of (11b): 2-(2-hydroxypropyl)-6-(naphthalen-2-yl)pyri-dazin-3(2*H*)-one S71
Fig. S95. ^{1}H NMR spectrum of (12): 2-phenylpyridine S72
Fig. S96. ^{13}C NMR spectrum of (12): 2-phenylpyridine S72
Fig. S97. ^{1}H NMR spectrum of (13): 4-phenylpyridine S73
Fig. S98. ^{13}C NMR spectrum of (13): 4-phenylpyridine S73
Fig. S99. ^{1}H NMR spectrum of (14): 2-(biphenyl-4-yl)pyridine S74
Fig. S100. ^{13}C NMR spectrum of (14): 2-(biphenyl-4-yl)pyridine..S74
Fig. S101. ^{1}H NMR spectrum of (15): 4-(biphenyl-4-yl)pyridine S75
Fig. S102. ^{13}C NMR spectrum of (15): 4-(biphenyl-4-yl)pyridine..S75
Fig. S103. ^{1}H NMR spectrum of (16): 2-(4-fluorophenyl)pyridine..S76
Fig. S104. ^{13}C NMR spectrum of (16): 2-(4-fluorophenyl)pyridine..S76
Fig. S105. ^{1}H NMR spectrum of (17): 4-(4-fluorophenyl)pyridine..S77
Fig. S106. ^{13}C NMR spectrum of (17): 4-(4-fluorophenyl)pyridine..S77
Fig. S107. ^{1}H NMR spectrum of (18a): 6-phenylpyridazin-3(2*H*)-one S78
Fig. S108. ^{13}C NMR spectrum of (18a): 6-phenylpyridazin-3(2*H*)-one S78
Fig. S109. ^{1}H NMR spectrum of (18b): 2-(2-hydroxypropyl)-6-phenylpyridazin-3(2*H*)-one.........S79
Fig. S110. ^{13}C NMR spectrum of (18b): 2-(2-hydroxypropyl)-6-phenylpyridazin-3(2*H*)-one........S79
Fig. S111. ^{1}H NMR spectrum of (19a): 6-(biphenyl-4-yl)pyridazin-3(2*H*)-one.............................S80
Fig. S112. ^{13}C NMR spectrum of (19a): 6-(biphenyl-4-yl)pyridazin-3(2*H*)-one............................S80
Fig. S113. ^{1}H NMR spectrum of (19b): 6-(biphenyl-4-yl)-2-(2-hydroxypropyl)pyridazin-3(2*H*)-one S81
Fig. S114. ^{13}C NMR spectrum of (19b): 6-(biphenyl-4-yl)-2-(2-hydroxypropyl)pyridazin-3(2*H*)-one S81
Fig. S115. ^{1}H NMR spectrum of (20a): 6-(4-fluorophenyl)pyridazin-3(*2H*)-one S82
Fig. S116. ^{13}C NMR spectrum of (20a): 6-(4-fluorophenyl)pyridazin-3(*2H*)-one S82

Fig. S117. ^{1}H NMR spectrum of (20b): 6-(4-fluorophenyl)-2-(2-hydroxypropyl)pyri-dazin-3(*2H*)-one S83

Fig. S118. ^{13}C NMR spectrum of (20b): 6-(4-fluorophenyl)-2-(2-hydroxypropyl)pyri-dazin-3(*2H*)-one S83

Fig. S119. ^{1}H NMR spectrum of (23): 2-(2-hydroxypropyl)-6-iodopyridazin-3(*2H*)-one S84

Fig. S120. ^{13}C NMR spectrum of (23): 2-(2-hydroxypropyl)-6-iodopyridazin-3(*2H*)-one S84

References: S85

Experimental section

General procedures

3,6-dichloropyridazine, 57% aq. HI, phenylboronic acid and 4-biphenylboronic acid were purchased from Alfa Aesar. 2-naphtaleneboronic acid and 4-fluorobenzeneboronic acid were purchased from Lancaster. 2-iodopyridine and 4-iodopyridine were purchased from TCI, while the propylene carbonate was purchased from Sigma-Aldrich and tetrakis-(triphenylphosphine)palladium (0) from FluoroChem.
Thin-layer chromatography (TLC) was performed on aluminium sheets precoated with Merck 5735 Kieselgel 60F254. Column chromatography was carried out either with Merck 5735 Kieselgel 60F (0.040-0.063 nm mesh). All other chemicals and solvents were purchased from different commercial sources and used as received without further purification.
Freeze-drying was performed one night in a LYPH-Lock 1L lyophilizer LabConco (Kansas City, Missouri) with high vacuum pump, at 10 mmHg and -50 °C. Melting points were measured on a Büchi M-550 apparatus (Büchi Labortechnik AG, Switzerland) and are not corrected.

Method A

Suzuki cross-coupling reaction of iodopyridine isomers (Figure 3 and Figure 5)

In a two necked round bottom flask, covered by tinfoil and under argon was measured 2-, or 4-iodopyridine (1.00 mmol, d = 1.93 g/mL, 0.11 mL or 205 mg), the corresponding aromatic boronic acid: **5, 6, 7** or **8** (1.25 mmol), 0.5M Na_2CO_3 (1 mL), tetrakis(triphenyl-phosphine)palladium (0) (0.05 mmol, 58 mg) and propylene carbonate (5 mL). The reaction mixture was heated by oil-bath at max. 130 °C, refluxed for different time (**Table 1**) and monitored by TLC. After completion of the reaction, the suspension was cooled down and water (25 mL) was added. The aqueous layer was extracted with dichloromethane (2x25 mL), followed by neutralization with few drops of 5% H_2SO_4 and again extracted with dichloromethane (2x25 mL). The collected organic phase was washed with 10% $CuSO_4$ solution (2x15 mL) and evaporated, after drying over Na_2SO_4 and filtration. The crude product was lyophilized overnight at 10 mmHg and -50°C and weighted the mass of raw product. In all new reactions the product was purified by column chromatography and the pure products: **9, 10, 12, 13, 14, 15, 16** and **17** were characterized by ^{1}H-, ^{13}C-NMR-spectroscopy and determined the max. absorbance by HPLC-UV method. For the HPLC-MS-analysis we take off sample from the raw product after freeze-drying and from the purified one too.

Suzuki cross-coupling reaction of 6-iodopyridazin-3(2*H*)-one (Figure 4 and Figure 6)

In a two necked round bottom flask, under argon was measured 6-iodopyridazin-3(2*H*)-one (2.00 mmol, 444 mg), the corresponding aromatic boronic acid: **5, 6, 7** or **8** (2.50 mmol), 0.5M Na_2CO_3 (2 mL), tetrakis(triphenylphosphine)palladium (0) (0.10 mmol, 116 mg) and propylene carbonate (5 mL). The reaction mixture was heated by oil-bath at max. 130 °C, refluxed for different time (**Table 1**) and monitored by TLC. After completion of the reaction, the suspension was cooled down and water (50 mL) was added. The aqueous layer was extracted with chloroform (3x25 mL), followed by neutralization with few drops of 5% H_2SO_4 and again extracted with chloroform (3x25 mL). The collected organic phase was washed with 10% $CuSO_4$ solution (3x15 mL) and evaporated, after drying over Na_2SO_4 and filtration. The crude product was lyophilized overnight at 10 mmHg and -50°C and weighted

the mass of raw product. In the new reactions the products: **11a** and **11b, 18a** and **18b, 19a** and **19b, 20a** and **20b** were separated by column chromatography (silica gel, 0.040–0.063 mesh size). The separated pure products were characterized by ^{1}H-, ^{13}C-NMR-spectroscopy and determined the max. absorbance by HPLC-UV method. For the HPLC-MS-analysis we take off sample from the raw product after freeze-drying and from the purified too.

Method B: Reactions under MW conditions

MW assisted experiments were carried out in a monomode CEM-Discover MW reactor, using the standard configuration as delivered, including proprietary software. The experiments were executed in 80 mL MW process vials, dynamic method with control of the temperature by infrared detection. Conditions: 5 min. ramp rime, 130 °C temperature, 60 min. hold time, max. 200 Psi pressure and 300 W power. After completion of the reaction, the vial was cooled to 50 °C by air jet cooling, followed by usual work-up. In the Suzuki reaction of 2- and 4-iodopyridine the work-up is described in Method A, and in the case of 6-iodopyridazin-3(2*H*)-on was similar, but two products were prepared. Sample were taken of for NMR-spectroscopy, as well as for HPLC-MS-measurements.

NMR measurements

^{1}H(400 MHz) and ^{13}C(100 MHz) NMR spectra were recorded at room temperature on Varian Mercury Plus spectrometer. Amounts of 10-15 mg of compounds were dissolved in 0.6 ml $CDCl_3$ or DMSO-d_6 and transferred to 5 mm NMR sample tubes. Chemical shifts are given on the δ-scale and referenced to the solvent ($CDCl_3$: δ_C=77.00 ppm and δ_H=7.27 ppm or DMSO-d_6: δ_C=39.50 ppm and δ_H=2.50 ppm). For ^{1}H NMR measurement 24 K data points, 2.0 s acquisition time and 6400 Hz sweep width were used. ^{13}C spectra were recorded with 62 K data points and 24000 Hz sweep width. For 2D measurements, in case of the gHSQC spectrum the sweep width in F2 was 3000 Hz; data points (t2 x t1) were acquired with 1 K x 128, in case of the gHMBC spectrum the sweep width in F2 was 3000 Hz; data points (t2 x t1) were acquired with 1 K x 256, respectively.

HPLC-UV method for measuring max. absorbance of the compounds

The analysis was carried out with an Agilent LC MSD 1100 High Performance Liquid Chromatograph (Agilent Technologies Inc. Palo Alto, CA, USA) with HP Chemstation software Rev. A10.02 (Agilent, Waldbronn, Germany). The system consisted of a binary pump, on-line degasser, autosampler, column heater and diode array detector (DAD). An Agilent Zorbax Esclipse Plus C18, 3 mm×150 mm, 3.5 μm column (Agilent Technologies, Waldbronn, Germany) was used for the separation. Column temperature was 40°C. The concentration of the sample was 1 mg/mL and the solvent was acetonitrile. This concentrated solution was diluted with water to: 0.1 mg/mL. Sample injection volume was 10 μL, the flow rate was 1.0 mL min^{-1} and the run time was 10 min. Gradient elution was applied, eluent A contained water with 0.05 % formic acid, eluent B contained acetonitrile with 0.05 % formic acid. The used gradient can be seen in **Table S1**. Detections were in 210–400 nm wavelength range and the maximum wavelenght (λmax) of the products was summarized in **Table S2.**

TEM measurements

Samples were dissolved in methanol and dried on Formvar coated 200 mesh copper grids and examined in a JEOL JEM 1011 transmission electron microscope operating at 80 kV. Images were taken with an Olympus Morada 11 megapixel camera and iTEM software (Olympus).

Table S1. Gradient elution in HPLC-UV method

**Time (min.)**	A(%)	B(%)
**0.0**	95.0	5.0
**8.0**	0.0	100.0

A. water with 0.05% formic acid, B. acetonitrile with 0.05% formic acid.

Table S2: Measured maximum absorbance by HPLC-UV method.

**λ max. (nm)**	**2** (270, 218)	**3** (262)	**4** (228)
**5** (228)	**9** (308, 246)	**10** (316, 274)	**11a, 11b** (254)
**6** (268, 218)	**12** (294, 242)	**13** (288)	**18a, 18b** (250)
**7** (260)	**14** (300)	**15** (322, 236)	**19a, 19b** (278)
**8** (262, 214)	**16** (294, 242)	**17** (292)	**20a, 20b** (250)

HPLC-MS method for the characterization of products purity

The HPLC-MS system consists of a Shimadzu LC-20AD parallel pumps, with Shimadzu DG-20As Degasser, Shimadzu SIL-20A HT Autosampler, CTO-20A thermostat at 40 °C, and LCMS-2020 mass spectrometer, equipped with ESI ionisator (Drying gas 15L/min, Nebulizing gas 1.5 L/min; the DL temp is 250 °C, ESI 10 000 V). The HPLC column was a Supelco C-18 5µm, 50 x 4.6 mm. The eluent A was 0.1% TFA in water (from Merck); eluent B was 0.1% TFA in acetonitrile (from Merck). The gradient was set by a linear program, where eluent B was increased from 0% to 100%, within 10 min (**Table S3**). The flow rate was 1 ml/min. The obtained retention times measured on 254 nm are summarized in **Table S4.**

Table S3. Gradient elution in HPLC-MS method

**Time (min.)**	A(%)	B(%)
**0.0**	95.0	5.0
**10.0**	0.0	100.0

A. water with 0.1% TFA, B. acetonitrile with 0.1% TFA.

Table S4: Retention time measured on 254 nm.

**Product rt (min.)**	**5**	**6**	**7**	**8**
**2**	**9** (6.8)	**12** (5.3)	**14** (7.4)	**16** (5.6)
**3**	**10** (6.9)	**13** (5.7)	**15** (7.5)	**17** (5.9)

**4**	**11a** (7.8)	**18a** (6.6)	**19a** (8.4)	**20a** (6.8)
	**11b** (8.1)	**18b** (7.1)	**19b** (8.7)	**20b** (7.3)

The substrate **4** (**Figure 1**) was prepared according to literature procedures:
3,6-diiodopyridazine: A mixture of 6.00 g (40.27 mmol) 3,6-dichloropyridazine and 90 mL 57% (in water) HI (d = 1.7 g/mL, 51.3 mL, 681 mmol) was refluxed 5h at max. 120-130 °C (oil temperature). After cooling down, the reaction mixture was poured on ice, neutralized with 4M NaOH and filtered. The solid was washed with 2x50 mL water, 2x50 mL sat. solution of sodium thiosulfate. The crude product was recrystallized from 100 mL EtOAc[1]. Yield: 9.40 g yellow solid (70%), Mp: 164.1-166.3 °C[2], HPLC (254 nm): 100%, rt = 6.8 min., $C_4H_2I_2N_2$: 331.88, m/z = 333, ^{1}H NMR (400 MHz, $CDCl_3$) δ 7.50 (s, 2H, C*H*), ^{13}C NMR (100 MHz, $CDCl_3$) δ 137.9, 124.7.
6-iodopyridazine-3(2H)-one (4): A mixture of 9.36 g (28.19 mmol) 3,6-diiodopyridazine and 58 mL 8% (in water) NaOH (4.60 g, 115 mmol) was refluxed 2h at max. 110-120 °C (oil temperature). After cooling down, the reaction mixture was poured on ice, neutralized with 50% AcOH and filtered. The solid was washed with 2x30 mL water and the crude product was recrystallized from 70 mL EtOAc. Yield: 4.90 g white solid (78%), Mp: 170.5-172.3 °C[3], HPLC (254 nm): 100%, rt = 2.0 min., $C_4H_3IN_2O$: 221.98, m/z = ^{1}H NMR (400 MHz, DMSO-*d$_6$*) δ 13.31 (brs, 1H, N*H*), 7.62 (d, *J*=9.7 Hz, 1H, OCC*H*), 6.65 (d, *J*=9.7 Hz, 1H, ICC*H*), ^{13}C NMR (100 MHz, DMSO-*d$_6$*) δ 159.6, 142.1, 131.3, 102.1.

2-(naphthalen-2-yl)pyridine (9):
Reagents refluxed 5h according to Method A. Crude product purified by column chromatography (silica gel, 0.040–0.063 mesh size) with net dichloromethane as eluent. Yield: 188 mg yellow solid (92%), Mp: 68.7-70.5 °C[4, 5], HPLC (254 nm): 100%, rt = 6.8 min., $C_{15}H_{11}N$: 205.25, m/z = 206, ^{1}H NMR (400 MHz, $CDCl_3$) δ 8.76 (d, *J*= 4.3 Hz, 1H, NC*H*), 8.49 (s, 1H, CC*H*C), 8.14 (dd, *J*=8.6, 1.7 Hz, 1H, pyr-CC*H*CHC), 8.00-7.92 (m, 2H, CCHCC*H*C*H*), 7.92-7.84 (m, 2H, NCC*H*, CCHCHCC*H*), 7.80 (td, *J*=7.6, 1.7 Hz, 1H, NCCHC*H*), 7.55-7.48 (m, 2H, pyr-CC*H*CHC, CCHCHCCHC*H*), 7.27 (m, 1H, NCHC*H*), ^{13}C NMR (100 MHz, $CDCl_3$) δ 157.3, 149.8, 136.8, 136.6, 133.6, 133.5, 128.7, 128.4, 127.6, 126.5, 126.3, 126.3, 124.5, 122.2, 120.8.
Repetition of Method A: 210 mg crude product, HPLC (254 nm): 86.53%, estimated yield: 182 mg (89%).

Reagents refluxed 1h according to Method B. Crude product purified by column chromatography (silica gel, 0.040–0.063 mesh size) with net dichloromethane as eluent. Yield: 190 mg yellow solid (93%), HPLC (254 nm): 100%, rt = 6.8 min., $C_{15}H_{11}N$: 205.25, m/z = 206.
Repetition of Method B: 220 mg crude product, HPLC (254 nm): 82.26%, estimated yield: 181 mg (88%).

4-(naphthalen-2-yl)pyridine (10):
Reagents refluxed 3h according to Method A. Crude product purified by column chromatography (silica gel, 0.040–0.063 mesh size) with gradient elution: net dichloromethane, followed by dichloromethane : ethyl acetate 5:1, respectively 1:1. Yield: 174 mg yellow solid (85%), Mp: 103.8-105.6 °C[6-8], HPLC (254 nm): 100%, rt = 6.9 min.,

$C_{15}H_{11}N$: 205.25, m/z = 206, ^{1}H NMR (400 MHz, $CDCl_3$) δ 8.72 (brs, 2H, NC*H*), 8.12 (s, 1H, CC*H*C), 8.01-7.54 (m, 3H, CCHC*H*CC*H*, CCHCC*H*,), 7.75 (dd, *J*=8.5, 1.7 Hz, 1H, CC*H*CHC), 7.72-7.60 (m, 2H, NCHC*H*), 7.60-7.51 (m, 2H, CCHCCHC*H*C*H*), ^{13}C NMR (100 MHz, $CDCl_3$) δ 149.8, 148.6, 135.2, 133.4, 133.4, 129.0, 128.4, 127.7, 126.9, 126.7, 126.5, 124.5, 122.0.
Repetition of Method A: 200 mg crude product, HPLC (254 nm): 83.79%, estimated yield: 168 mg (82%).

Reagents refluxed 1h according to Method B. Crude product purified by column chromatography (silica gel, 0.040–0.063 mesh size) with gradient elution: net dichloromethane, followed by dichloromethane : ethyl acetate 5:1, respectively 1:1. Yield: 175 mg yellow solid (85%), HPLC (254 nm): 100%, rt = 6.9 min., $C_{15}H_{11}N$: 205.25, m/z = 206.
Repetition of Method B: 200 mg crude product, HPLC (254 nm): 88.02%, estimated yield: 176 mg (86%).

6-(naphthalen-2-yl)pyridazin-3(2*H*)-one (11a) and 2-(2-hydroxypropyl)-6-(naphthalen-2-yl)pyridazin-3(2*H*)-one (11b):

Reagents refluxed 4h according to Method A. Crude product purified by column chromatography (silica gel, 0.040–0.063 mesh size) with gradient elution: net chloroform, followed by chloroform : ethyl acetate 10:1, respectively 5:1.
Product **11a:** 85 mg white solid (19%), Mp: 249.7-252.1 °C[9, 10], HPLC (254 nm): 100%, rt = 7.8 min., $C_{14}H_{10}N_2O$: 222.24, m/z = 223, ^{1}H NMR (400 MHz, DMSO-*d₆*) δ 13.29 (brs, 1H, N*H*), 8.45 (s, 1H, pyr-CC*H*C), 8.23 (d, *J*=9.9 Hz, 1H, NCC*H*), 8.09-7.89 (m, 4H, pyr-CC*H*C*H*CC*H*, pyr-CCHCC*H*), 7.60-7.54 (m, 2H, pyr-CCHCCHC*H*C*H*), 7.06 (d, *J*=9.9 Hz, 1H, NHCOC*H*), ^{13}C NMR (100 MHz, DMSO-d_6) δ 160.3, 143.6, 133.0, 132.9, 132.0, 131.5, 130.2, 128.6, 128.5, 127.6, 127.0, 126.7, 125.2, 123.0.
Product **11b:** 110 mg light yellow solid (20%), Mp: 116.5-118.1 °C, HPLC (254 nm): 96.68%, rt = 8.1 min., $C_{17}H_{16}N_2O_2$: 280.32, m/z = 281, ^{1}H NMR (400 MHz, DMSO-d_6) δ 8.46 (s, 1H, pyr-CC*H*C), 8.22 (d, *J*=9.7 Hz, 1H, NCOCHC*H*), 8.07 (dd, *J*=8.6, 1.6 Hz, 1H, pyr-CC*H*CH), 8.05-7.99 (m, 3H, pyr-CCHC*H*CC*H*, pyr-CCHCC*H*), 7.61-7.53 (m, 2H, pyr-CCHCCHC*H*C*H*), 7.10 (d, *J*=9.7 Hz, 1H, NCOC*H*), 4.90 (d, *J*=4.9 Hz, 1H, O*H*), 4.27-4.12 (m, 2H, NCH_2C*H*), 4.11-3.97 (m, 1H, NCH_2C*H*), 1.15 (d, *J*=5.6 Hz, 3H, CH_3), ^{13}C NMR (100 MHz, DMSO-d_6) δ 159.3, 143.0, 133.1, 132.9, 131.9, 130.5, 129.7, 128.6, 128.5, 127.6, 127.0, 126.8, 125.3, 123.2, 64.1, 58.3, 21.1.
Repetition of Method A: Product **11a:** 95 mg white solid (21%), Mp: 246.5-250.0 °C[9, 10], HPLC (254 nm): 97.11%, rt = 7.8 min., $C_{14}H_{10}N_2O$: 222.24, m/z = 223. Product **11b:** 117 mg light yellow solid (19%), Mp: 121.7-128.9 °C, HPLC (254 nm): 82.14%, rt = 8.1 min., $C_{17}H_{16}N_2O_2$: 280.32, m/z = 281.

Reagents refluxed 1h according to Method B. Crude product purified by column chromatography (silica gel, 0.040–0.063 mesh size) with gradient elution: net chloroform, followed by chloroform : ethyl acetate 10:1, respectively 5:1. Product **11a:** 158 mg white solid (36%), HPLC (254 nm): 94.58%, rt = 7.8 min., $C_{14}H_{10}N_2O$: 222.24, m/z = 223. Product **11b:** 292 mg light yellow solid (52%), HPLC (254 nm): 96.65%, rt = 8.1 min., $C_{17}H_{16}N_2O_2$: 280.32, m/z = 281.
Repetition of Method B: 500 mg crude product, HPLC (254 nm): 42.58% **11a** and 55.67% **11b**, estimated yield: 215 mg (48%) **11a** and 280 mg (50%) **11b.**

2-phenylpyridine (12):

Reagents refluxed 7h according to Method A. Crude product purified by column chromatography (silica gel, 0.040–0.063 mesh size) with gradient elution: net chloroform, followed by chloroform : ethyl acetate 10:1, respectively 5:1. Yield: 200 mg yellow solid (65%), Mp: 75.1-76.8 °C[11-13], HPLC (254 nm): 89.97%, rt = 5.2 min., $C_{11}H_9N$: 155.20, m/z = 156, ^{1}H NMR (400 MHz, $CDCl_3$) δ 8.71 (d, *J*=4.3 Hz, 1H, NC*H*), 8.00 (d, *J*=7.3 Hz, 2H, orto-phenyl H), 7.81-7.68 (m, 2H, NCC*H*C*H*), 7.53-7.37 (m, 3H, meta-, para-phenyl H), 7.31-7.20 (m, 1H, NCHC*H*), ^{13}C NMR (100 MHz, $CDCl_3$) δ 157.5, 149.7, 139.4, 136.7, 128.9, 128.7, 126.9, 122.1, 120.6.
Repetition of Method A: 150 mg crude product, HPLC (254 nm): 66.16%, estimated yield: 99 mg (64%).

Reagents refluxed 1h according to Method B. Crude product purified by column chromatography (silica gel, 0.040–0.063 mesh size) with net chloroform, followed by chloroform : ethyl acetate 10:1, respectively 5:1. Yield: 100 mg yellow solid (65%), HPLC (254 nm): 97.53%, rt = 5.3 min., $C_{11}H_9N$: 155.20, m/z = 156.
Repetition of Method B: 120 mg crude product, HPLC (254 nm): 86.45%, estimated yield: 104 mg (67%).

4-phenylpyridine (13):

Reagents refluxed 3h according to Method A. Crude product purified by column chromatography (silica gel, 0.040–0.063 mesh size) with gradient elution: net chloroform, followed by chloroform : ethyl acetate 10:1, respectively 5:1. Yield: 73 mg yellow solid (47%), Mp: 68.3-69.4 °C[14-16], HPLC (254 nm): 100%, rt = 5.5 min., $C_{11}H_9N$: 155.20, m/z = 156, ^{1}H NMR (400 MHz, $CDCl_3$) δ 8.68 (d, *J*=4.4 Hz, 2H, NC*H*), 7.66 (d, *J*=7.4 Hz, 2H, orto-phenyl H), 7.56-7.41 (m, 5H, NCHC*H*, meta-, para-phenyl H), ^{13}C NMR (100 MHz, $CDCl_3$) δ 150.2, 148.4, 138.1, 129.1, 129.1, 127.0, 121.7.
Repetition of Method A: 200 mg crude product, HPLC (254 nm): 36.77%, estimated yield: 73 mg (47%).

Reagents refluxed 1h according to Method B. Crude product purified by column chromatography (silica gel, 0.040–0.063 mesh size) with gradient elution: net chloroform, followed by chloroform : ethyl acetate 10:1, respectively 5:1. Yield: 115 mg yellow solid (74%), HPLC (254 nm): 97.60%, rt = 5.8 min., $C_{11}H_9N$: 155.20, m/z = 156.
Repetition of Method B: 250 mg crude product, HPLC (254 nm): 42.58%, estimated yield: 106 mg (69%).

2-(biphenyl-4-yl)pyridine (14):

Reagents refluxed 3h according to Method A. Crude product purified by column chromatography (silica gel, 0.040–0.063 mesh size) with dichloromethane as eluent. Yield: 220 mg yellow solid (95%), Mp: 136.3-138.4 °C[17, 18], HPLC (254 nm): 100%, rt = 7.4 min., $C_{17}H_{13}N$: 231.29, m/z = 232, ^{1}H NMR (400 MHz, DMSO-d_6) δ 8.69 (d, *J*=4.3 Hz, 1H, NC*H*), 8.20 (d, *J*=8.3 Hz, 2H, NCCC*H*), 8.03 (d, *J*=8.0 Hz, 1H, NCC*H*), 7.90 (tm, *J*=8.0 Hz, 1H, NCCHC*H*), 7.81 (d, *J*=8.3 Hz, 2H, NCCCHC*H*), 7.75 (d, *J*=7.4 Hz, 2H, orto-phenyl H), 7.50 (t, *J*=7.4 Hz, 2H, meta-phenyl H), 7.44-7.33 (m, 2H, NCHC*H*, para-phenyl H), ^{13}C NMR (100 MHz, DMSO-d_6) δ 155.5, 149.6, 140.6, 139.5, 137.7, 137.3, 129.0, 127.7, 127.1, 127.0, 126.7, 122.7, 120.2.
Repetition of Method A: 255 mg crude product, HPLC (254 nm): 80.59%, estimated yield: 205 mg (89%).

Reagents refluxed 1h according to Method B. Crude product purified by column chromatography (silica gel, 0.040–0.063 mesh size) with dichloromethane as eluent. Yield: 225 mg yellow solid (97%) HPLC (254 nm): 100%, rt = 7.4 min., $C_{17}H_{13}N$: 231.29, m/z = 232.
Repetition of Method B: 270 mg crude product, HPLC (254 nm): 78.96%, estimated yield: 213 mg (92%).

4-(biphenyl-4-yl)pyridine (15):
Reagents refluxed 2h according to Method A. Crude product purified by column chromatography (silica gel, 0.040–0.063 mesh size) with gradient elution: net dichloromethane, followed by dichloromethane : ethyl acetate 10:1, 5:1, respectively 1:1. Yield: 212 mg light yellow solid (92%), Mp: 201.7-203.9 °C[19, 20], HPLC (236 nm): 92.41%, rt = 7.5 min., $C_{17}H_{13}N$: 231.29, m/z = 232, ^{1}H NMR (400 MHz, $CDCl_3$) δ 8.78 (brs, 2H, NC*H*), 7.78-7.70 (m, 4H, pyr-CC*H*C*H*C), 7.70-7.54 (m, 4H, NCHC*H*, orto-phenyl H), 7.48 (t, *J*=7.4 Hz, 2H, meta-phenyl H), 7.43-7.36 (m, 1H, para-phenyl H), ^{13}C NMR (100 MHz, $CDCl_3$) δ 149.8, 148.0, 142.2, 142.1, 141.6, 140.2, 136.7, 134.2, 128.9, 128.7, 127.8, 127.8, 127.4, 127.2, 127.1, 126.4, 121.9.
Repetition of Method A: 350 mg crude product, HPLC (254 nm): 57.51%, estimated yield: 201 mg (87%).

Reagents refluxed 1h according to Method B. Crude product purified by column chromatography (silica gel, 0.040–0.063 mesh size) with gradient elution: net dichloromethane, followed by dichloromethane : ethyl acetate 10:1, 5:1, respectively 1:1. Yield: 210 mg yellow solid (91%), HPLC (236 nm): 92.41%, rt = 7.5 min., $C_{17}H_{13}N$: 231.29, m/z = 232.
Repetition of Method B: 350 mg crude product, HPLC (254 nm): 55.17%, estimated yield: 194 mg (84%).

2-(4-fluorophenyl)pyridine (16):
Reagents refluxed 3h according to Method A. Crude product purified by column chromatography (silica gel, 0.040–0.063 mesh size) with gradient elution: net dichloromethane, followed by dichloromethane : ethyl acetate 10:1, 5:1, respectively 1:1. Yield: 74 mg yellow oil (43%)[21-23], HPLC (254 nm): 100%, rt = 5.6 min., $C_{11}H_8FN$: 173.19, m/z = 174, ^{1}H NMR (400 MHz, $CDCl_3$) δ 8.68 (d, *J*=4.1 Hz, 1H, NC*H*), 8.06-7.91 (m, 2H, FCCHC*H*), 7.76 (tm, *J*=7.7 Hz, 1H, NCCHC*H*), 7.69 (d, *J*=7.7 Hz, 1H, NCC*H*), 7.24 (m, 1H, NCHC*H*), 7.21-7.08 (m, 2H, FCC*H*), ^{13}C NMR (100 MHz, $CDCl_3$) δ 163.5 (d, *J*=248.6 Hz), 156.4, 149.7, 136.8, 135.5 (d, *J*=3.1 Hz), 128.7 (d, *J*=8.3 Hz), 122.1, 120.3, 115.7 (d, *J*=21.7 Hz).
Repetition of Method A: 120 mg crude product, HPLC (254 nm): 84.08%, estimated yield: 101 mg (58%).

Reagents refluxed 1h according to Method B. Crude product purified by column chromatography (silica gel, 0.040–0.063 mesh size) with gradient elution: net dichloromethane, followed by dichloromethane : ethyl acetate 10:1, 5:1, respectively 1:1. Yield: 86 mg yellow oil (50%), HPLC (254 nm): 100%, rt = 5.6 min., $C_{11}H_8FN$: 173.19, m/z = 174.
Repetition of Method B: 120 mg crude product, HPLC (254 nm): 91.23%, estimated yield: 109 mg (63%).

4-(4-fluorophenyl)pyridine (17):

Reagents refluxed 1h according to Method A. Crude product purified by column chromatography (silica gel, 0.040–0.063 mesh size) with gradient elution: net dichloromethane, followed by dichloromethane : ethyl acetate 10:1, 5:1, respectively 1:1. Yield: 123 mg yellow oil (71%)[24-26], HPLC (254 nm): 100%, rt = 5.9 min., $C_{11}H_8FN$: 173.19, m/z = 174, ^{1}H NMR (400 MHz, $CDCl_3$) δ 8.86 (brs, 2H, NC*H*), 7.74-7.39 (m, 4H, NCHC*H*, FCCHC*H*), 7.24-7.10 (m, 2H, FCC*H*), ^{13}C NMR (100 MHz, $CDCl_3$) δ 163.5 (d, *J*=249.3 Hz), 150.0, 147.1, 134.3 (d, *J*=3.1 Hz), 132.1, 128.7 (d, *J*=8.3 Hz), 116.2 (d, *J*=21.9 Hz).

Repetition of Method A: 150 mg crude product, HPLC (254 nm): 90.54%, estimated yield: 136 mg (79%).

Reagents refluxed 1h according to Method B. Crude product purified by column chromatography (silica gel, 0.040–0.063 mesh size) with gradient elution: net dichloromethane, followed by dichloromethane : ethyl acetate 10:1, 5:1, respectively 1:1. Yield: 138 mg yellow oil (80%), HPLC (254 nm): 100%, rt = 5.9 min., $C_{11}H_8FN$: 173.19, m/z = 174.

Repetition of Method B: 150 mg crude product, HPLC (254 nm): 97.20%, estimated yield: 146 mg (84%).

6-phenylpyridazin-3(2*H*)-one (18a) and 2-(2-hydroxypropyl)-6-phenylpyridazin-3(2*H*)-one (18b):

Reagents refluxed 6h according to Method A. Crude product purified by column chromatography (silica gel, 0.040–0.063 mesh size) with gradient elution: net dichloromethane, followed by dichloromethane : ethyl acetate 5:1, respectively 1:1.

Product **18a:** 160 mg light yellow solid (47%), Mp: 195.5-197.8 °C[9, 27, 28], HPLC (254 nm): 96.73%, rt = 6.6 min., $C_{10}H_8N_2O$: 172.18, m/z = 173, ^{1}H NMR (400 MHz, $CDCl_3$) δ 11.84 (brs, 1H, N*H*), 7.84-7.74 (m, 3H, NCC*H*, orto-phenyl H), 7.54-7.39 (m, 3H, meta-, para-phenyl H), 7.09 (d, *J*=9.9 Hz, 1H, COC*H*), ^{13}C NMR (100 MHz, $CDCl_3$) δ 161.4, 145.6, 134.4, 131.7, 130.4, 129.6, 129.0, 125.9.

Product **18b:** 240 mg yellow solid (52%), Mp: 95.7-97.1 °C[29, 30], HPLC (254 nm): 100%, rt = 7.1 min., $C_{13}H_{14}N_2O_2$: 230.26, m/z = 231, ^{1}H NMR (400 MHz, $CDCl_3$) δ 7.81-7.74 (m, 2H, orto-phenyl H), 7.73 (d, *J*=9.7 Hz, 1H, NCC*H*), 7.53-7.41 (m, 3H, meta-, para-phenyl H), 7.08 (d, *J*=9.7 Hz, 1H, COC*H*), 4.43-4.25 (m, 3H, O*H*, NC*H*$_2$C*H*), 3.67-3.58 (m, 1H, NC*H*$_2$CH), 1.32 (d, *J*=6.0 Hz, 3H, C*H*$_3$), ^{13}C NMR (100 MHz, $CDCl_3$) δ 161.1, 145.2, 134.4, 130.6, 130.3, 129.7, 129.1, 126.0, 67.6, 59.8, 21.0.

Repetition of Method A: 400 mg crude product, HPLC (254 nm): 34.52% **18a** and 65.48% **18b,** estimated yield: 138 mg (40%) **18a** and 262 mg (57%) **18b.**

Reagents refluxed 1h according to Method B. Crude product purified by column chromatography (silica gel, 0.040–0.063 mesh size) with gradient elution: net dichloromethane, followed by dichloromethane : ethyl acetate 5:1, respectively 1:1. Product **18a:** 155 mg light yellow solid (45%), HPLC (254 nm): 97.92%, rt = 5.8 min., $C_{10}H_8N_2O$: 172.18, m/z = 173. Product **18b:** 207 mg yellow solid (45%), HPLC (254 nm): 96.59%, rt = 6.4 min., $C_{13}H_{14}N_2O_2$: 230.26, m/z = 231.

Repetition of Method B: 400 mg crude product, HPLC (254 nm): 44.18% **18a** and 55.05% **18b**, estimated yield: 177 mg (51%) **18a** and 220 mg (48%) **18b.**

6-(biphenyl-4-yl)pyridazin-3(2*H*)-one (19a) and 6-(biphenyl-4-yl)-2-(2-hydroxypropyl)-pyridazin-3(2*H*)-one (19b):

Reagents refluxed 3h according to Method A. Crude product purified by column chromatography (silica gel, 0.040–0.063 mesh size) with gradient elution: net dichloromethane, followed by dichloromethane : ethyl acetate 10:1, 5:1, respectively 1:1.
Product **19a:** 160 mg light yellow solid (32%), Mp: 234.1-236.2 °C[31], HPLC (254 nm): 100%, rt = 8.4 min., $C_{16}H_{12}N_2O$: 248.28, m/z = 249, ^1H NMR (400 MHz, DMSO-d_6) δ 13.24 (brs, 1H, N*H*), 8.10 (d, *J*=9.9 Hz, 1H, NCC*H*), 7.96 (d, *J*=8.5 Hz, 2H, NCCCHC*H*), 7.79 (d, *J*=8.5 Hz, 2H, NCCC*H*), 7.73 (d, *J*=7.4 Hz, 2H, orto-phenyl H), 7.49 (t, *J*=7.4 Hz, 2H, meta-phenyl H), 7.39 (t, *J*=7.4 Hz, 1H, para-phenyl H), 7.01 (d, *J*=9.9 Hz, 1H, COC*H*), ^{13}C NMR (100 MHz, DMSO-d_6) δ 160.2, 143.4, 140.7, 139.2, 133.6, 131.4, 130.2, 129.0, 127.8, 127.1, 126.7, 126.2.
Product **19b:** 355 mg white solid (58%), Mp: 130.8-132.7 °C, HPLC (254 nm): 100%, rt = 8.7 min., $C_{19}H_{18}N_2O_2$: 306.36, m/z = 307, ^1H NMR (400 MHz, DMSO-d_6) δ 8.09 (d, *J*=9.7 Hz, 1H, NCC*H*), 7.99 (d, *J*=8.5 Hz, 2H, NCCCHC*H*), 7.80 (d, *J*=8.5 Hz, 2H, NCCC*H*), 7.74 (d, *J*=7.3 Hz, 2H, orto-phenyl H), 7.50 (t, *J*=7.3 Hz, 2H, meta-phenyl H), 7.40 (t, *J*=7.3 Hz, 1H, para-phenyl H), 7.06 (d, *J*=9.7 Hz, 1H, COC*H*), 4.89 (d, *J*=4.9 Hz, 1H, O*H*), 4.23-4.09 (m, 2H, NC*H*$_2$C*H*), 4.06-3.95 (m, 1H, NC*H*$_2$C*H*), 1.13 (d, *J*=5.8 Hz, 3H, C*H*$_3$), ^{13}C NMR (100 MHz, DMSO-d_6) δ 159.3, 142.8, 140.8, 139.3, 133.5, 130.4, 129.6, 129.1, 127.9, 127.1, 126.7, 126.4, 64.1, 58.3, 21.0.
Repetition of Method A: 680 mg crude product, HPLC (254 nm): 15.61% **19a** and 68.45% **19b**, estimated yield: 106 mg (21%) **19a** and 465 mg (76%) **19b.**

Reagents refluxed 1h according to Method B. Crude product purified by column chromatography (silica gel, 0.040–0.063 mesh size) with gradient elution: net dichloromethane, followed by dichloromethane : ethyl acetate 10:1, 5:1, respectively 1:1. Product **19a:** 130 mg light yellow solid (26%), HPLC (254 nm): 84.24%, rt = 8.4 min., $C_{18}H_{12}N_2O$: 248.28, m/z = 249. Product **19b:** 302 mg white solid (49%), HPLC (254 nm): 95.63%, rt = 8.7 min., $C_{19}H_{18}N_2O_2$: 306.36, m/z = 307.
Repetition of Method B: 600 mg crude product, HPLC (254 nm): 24.34% **19a** and 52.18% **19b**, estimated yield: 146 mg (29%) **19a** and 313 mg (51%) **19b.**

6-(4-fluorophenyl)pyridazin-3(2*H*)-one (20a) and 6-(4-fluorophenyl)-2-(2-hydroxypropyl)pyridazin-3(2*H*)-one (20b):

Reagents refluxed 3h according to Method A. Crude product purified by column chromatography (silica gel, 0.040–0.063 mesh size) with gradient elution: net chloroform, followed by chloroform : ethyl acetate 10:1, 5:1, respectively 1:1.
Product **20a:** 126 mg white solid (33%), Mp: 266.7-268.9 °C[32-34], HPLC (254 nm): 100%, rt = 6.8 min., $C_{10}H_7FN_2O$: 190.17, m/z = 191, ^1H NMR (400 MHz, DMSO-d_6) δ 13.21 (brs, 1H, N*H*), 8.04 (d, *J*=9.9 Hz, 1H, NCC*H*), 7.97-7.84 (m, 2H, FCCHC*H*), 7.39-7.23 (m, 2H, FCC*H*), 7.00 (d, *J*=9.9 Hz, 1H, COC*H*), ^{13}C NMR (100 MHz, DMSO-d_6) δ 162.8 (d, *J*=246.7 Hz), 160.2, 143.0, 131.4, 131.2 (d, *J*=3.0 Hz), 130.2, 127.9 (d, *J*=8.6 Hz), 115.8 (d, *J*=21.8 Hz).
Product **20b:** 280 mg yellow solid (56%), Mp: 136.3-138.8 °C, HPLC (254 nm): 96.19%, rt = 7.3 min., $C_{13}H_{13}FN_2O_2$: 248.25, m/z = 249, ^1H NMR (400 MHz, DMSO-d_6) δ 8.03 (d, *J*=9.7 Hz, 1H, COCHC*H*), 7.98-7.88 (m, 2H, FCCHC*H*), 7.39-7.27 (m, 2H, FCC*H*), 7.04 (d, *J*=9.7 Hz, 1H, COC*H*), 4.86 (d, *J*=4.5 Hz, 1H, O*H*), 4.19-4.06 (m, 2H, NC*H*$_2$C*H*), 4.04-3.92 (m, 1H, NC*H*$_2$CH), 1.11 (d, *J*=5.7 Hz, 3H, C*H*$_3$), ^{13}C NMR (100 MHz, DMSO-d_6) δ 162.8 (d,

J=246.0 Hz), 159.1, 142.3, 131.1 (d, *J*=2.9 Hz), 130.4, 129.7, 128.1 (d, *J*=8.6 Hz), 115.8 (d, *J*=21.8 Hz), 64.0, 58.2, 21.0.
Repetition of Method A: 430 mg crude product, HPLC (254 nm): 37.70% **20a** and 62.30% **20b,** estimated yield: 162 mg (43%) **20a** and 268 mg (54%) **20b.**

Reagents refluxed 1h according to Method B. Crude product purified by column chromatography (silica gel, 0.040–0.063 mesh size) with gradient elution: net chloroform, followed by chloroform : ethyl acetate 10:1, 5:1, respectively 1:1. Product **20a:** 168 mg white solid (44%), HPLC (254 nm): 100%, rt = 6.8 min., $C_{10}H_7FN_2O$: 190.17, m/z = 191. Product **20b:** 250 mg yellow solid (50%), HPLC (254 nm): 94.44%, rt = 7.3 min., $C_{13}H_{13}FN_2O_2$: 248.25, m/z = 249.
Repetition of Method B: 420 mg crude product, HPLC (254 nm): 39.73% **20a** and 60.27% **20b**, estimated yield: 167 mg (44%) **20a** and 253 mg (51%) **20b.**

2-(2-hydroxypropyl)-6-iodopyridazin-3(2*H*)-one (23) obtained without and with catalyst:

In an 80 mL vial was measured 6-iodopyridazin-3(2*H*)-one (0.80 mmol, 178 mg), 0.5M Na_2CO_3 (1.6 mL) and propylene carbonate (4 mL). In another experiment, to the before mentioned reagents was added tetrakis(triphenylphosphine)palladium (0) (0.04 mmol, 46 mg) The suspensions were reacted separated according to Method B. After 1h reaction time, the suspensions were cooled down and water (25 mL) was added. The aqueous layer was extracted with chloroform (3x10 mL), followed by neutralization with few drops of 5% H_2SO_4 and again extracted with chloroform (2x10 mL). The collected organic phase was washed with 10% $CuSO_4$ solution (2x10 mL) and evaporated, after drying over Na_2SO_4 and filtration. The crude product was lyophilized overnight at 10 mmHg and -50°C and weighted the mass of raw product.
Without catalyst: 180 mg yellow solid (80%), Mp: 119.1-122.0 °C, HPLC (254 nm): 91.26%, rt = 5.7 min., $C_7H_9IN_2O_2$: 280.06, m/z = 281.

Crude product purified by column chromatography (silica gel, 0.040–0.063 mesh size) with gradient elution: net dichloromethane, followed by dichloromethane : ethyl acetate 5:1, respectively 1:1.
With catalyst: 175 mg yellow solid (78%), Mp: 120.2-122.0 °C, HPLC (254 nm): 95.23%, rt = 5.7 min., $C_7H_9IN_2O_2$: 280.06, m/z = 281.
^{1}H NMR (400 MHz, $CDCl_3$-*d₆*) δ 7.42 (d, *J*=9.6 Hz, 1H, ICC*H*), 6.67 (d, *J*=9.6 Hz, 1H, OCC*H*), 4.31-4.09 (m 3H, C*H₂*C*H*OH), 1.27 (d, *J*=9.6 Hz, 3H, CHC*H*3) ^{13}C NMR (100 MHz, $CDCl_3$-*d₆*) δ 160.3, 141.6, 131.0, 99.4, 67.3, 59.6, 21.0.

2-(2-hydroxypropyl)-6-(naphthalen-2-yl)pyridazin-3(2*H*)-one (11b) from 6-(naphthalen-2-yl)pyridazin-3(2*H*)-one (11a):

In an 80 mL vial was measured 6-naphthalen-2-yl)pyridazin-3(2*H*)-one (**11a,** 0.50 mmol, 111 mg), 0.5M Na_2CO_3 (1.00 mL) and propylene carbonate (2.5 mL). The reaction mixture was reacted according to Method B. After 1h reaction time, the suspension was cooled down and water (20 mL) was added. The aqueous layer was extracted with chloroform (2x10 mL), followed by neutralization with few drops of 5% H_2SO_4 and again extracted with chloroform (2x10 mL). The collected organic phase was washed with 10% $CuSO_4$ solution (2x10 mL) and evaporated, after drying over Na_2SO_4 and filtration. The crude product was lyophilized overnight at 10 mmHg and -50°C and weighted the mass of raw product: 94 mg yellow solid (67%), Mp: 116.5-118.2 °C, HPLC (254 nm): 95.83%, rt = 8.1 min., $C_{17}H_{16}N_2O_2$: 280.32, m/z

= 281, ^1H NMR (400 MHz, DMSO-d_6) δ 8.46 (s, 1H, pyr-CC*H*C), 8.22 (d, *J*=9.7 Hz, 1H, NCOCHC*H*), 8.07 (dd, *J*=8.6, 1.6 Hz, 1H, pyr-CC*H*CH), 8.05-7.99 (m, 3H, pyr-CCHC*H*CC*H*, pyr-CCHCC*H*), 7.61-7.53 (m, 2H, pyr-CCHCCHC*H*C*H*), 7.10 (d, *J*=9.7 Hz, 1H, NCOC*H*), 4.90 (d, *J*=4.9 Hz, 1H, O*H*), 4.27-4.12 (m, 2H, NCH_2C*H*), 4.11-3.97 (m, 1H, NCH_2C*H*), 1.15 (d, *J*=5.6 Hz, 3H, CH_3), ^{13}C NMR (100 MHz, DMSO-d_6) δ 159.3, 143.0, 133.1, 132.9, 131.9, 130.5, 129.7, 128.6, 128.5, 127.6, 127.0, 126.8, 125.3, 123.2, 64.1, 58.3, 21.1.

Fig. S1: UV spectrum of 2-iodopyridine (2)

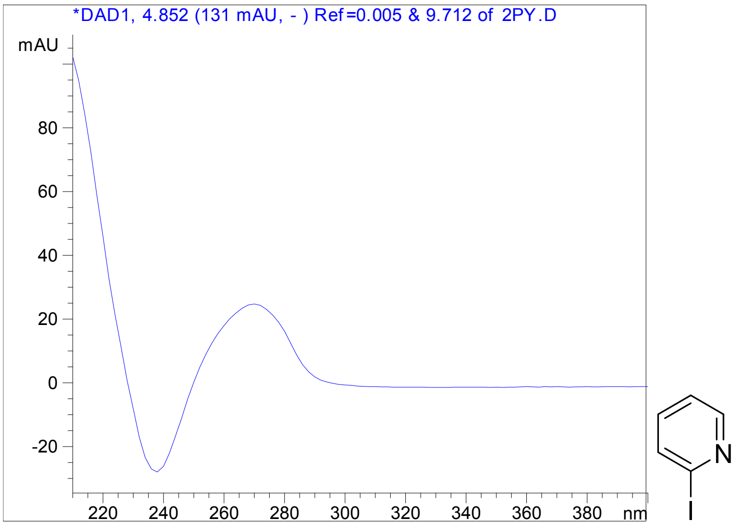

Fig. S2: UV spectrum of 4-iodopyridine (3)

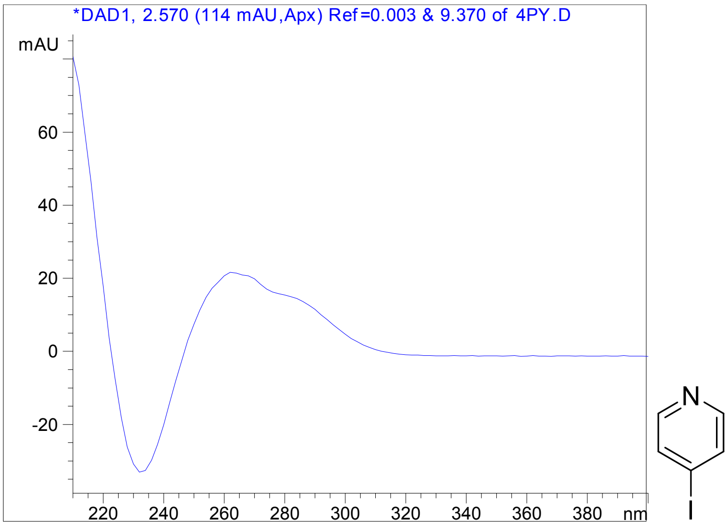

Fig. S3: UV spectrum of 6-iodopyridazin-3(2*H*)-one (4)

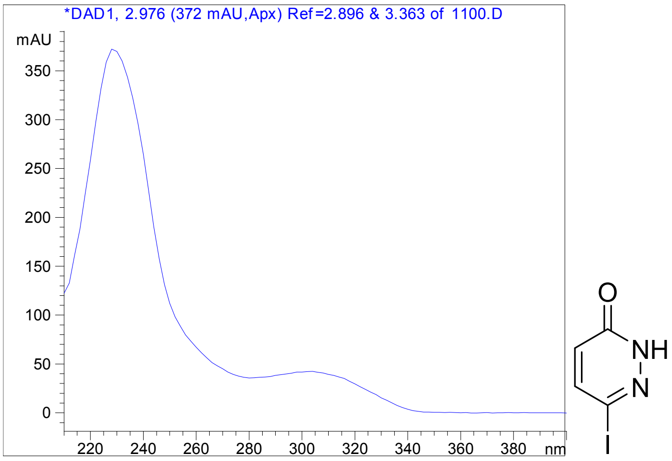

Fig. S4: UV spectrum of 2-naphthaleneboronic acid (5)

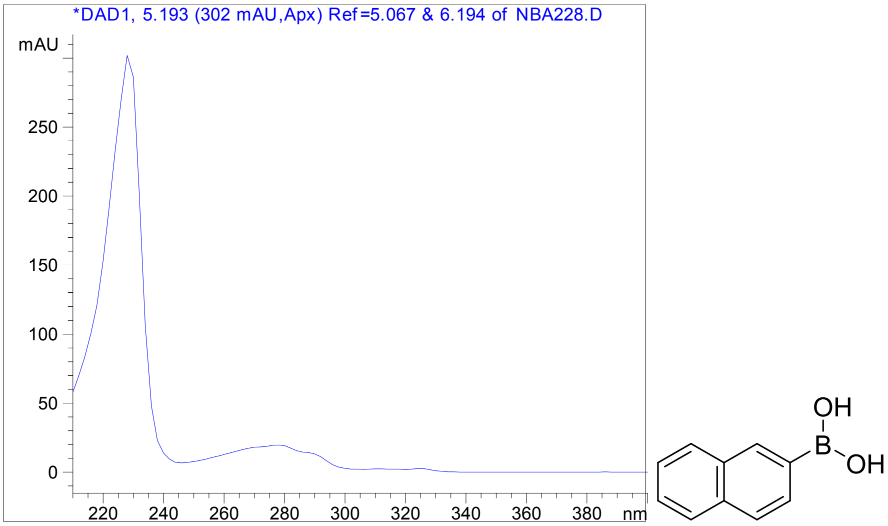

Fig. S5: UV spectrum of phenylboronic acid (6)

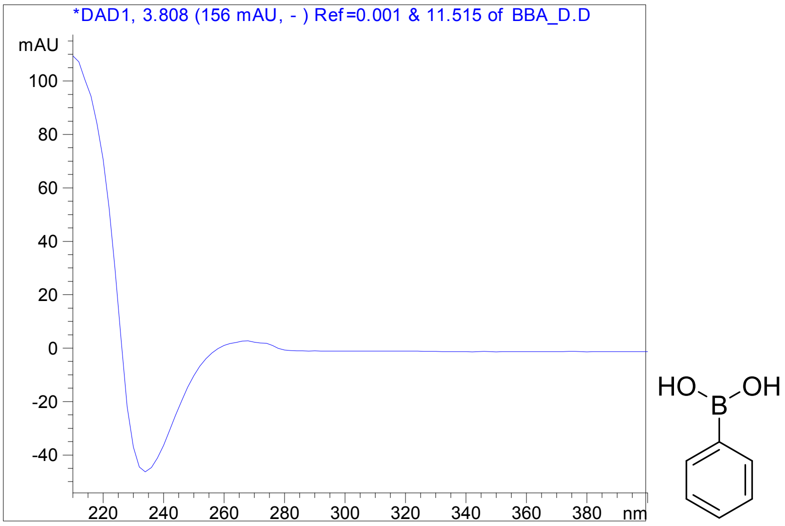

Fig. S6: UV spectrum of 4-biphenylboronic acid (7)

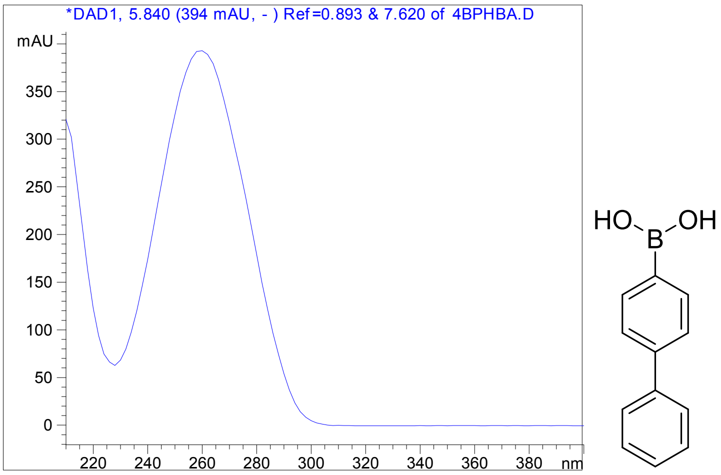

Fig. S7: UV spectrum of 4-fluorophenylboronic acid (8)

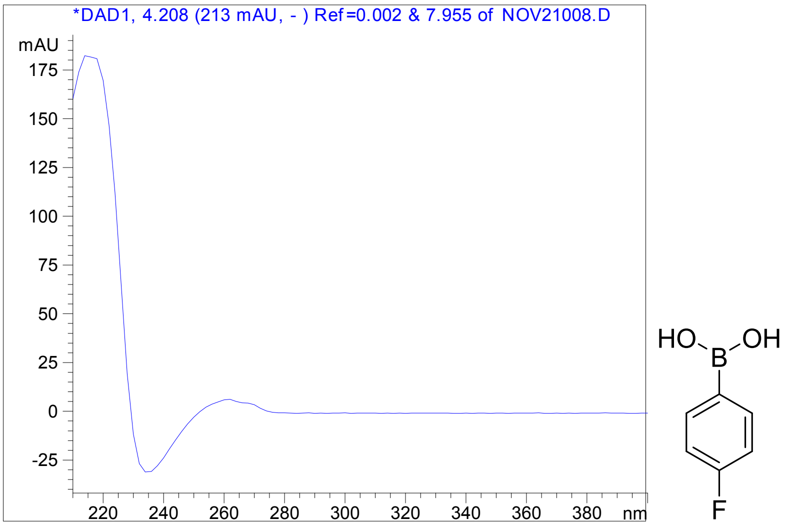

Fig. S8: UV spectrum of 2-(naphthalen-2-yl)pyridine (9)

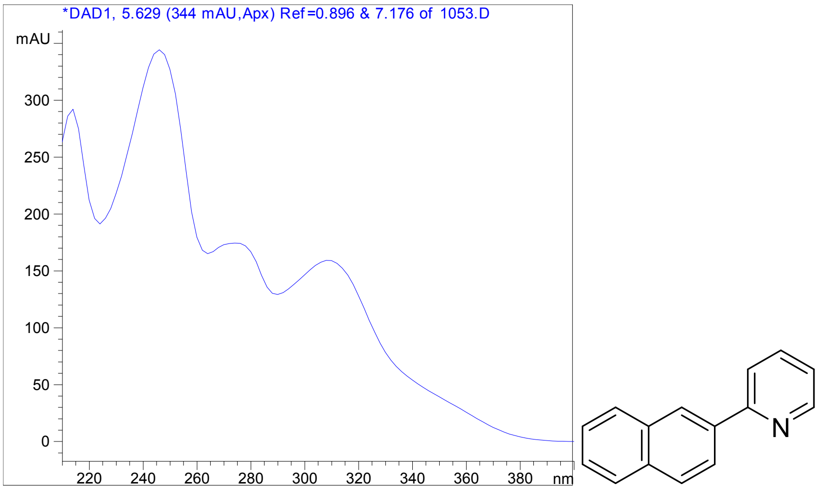

Fig. S9: UV spectrum of 4-(naphthalen-2-yl)pyridine (10)

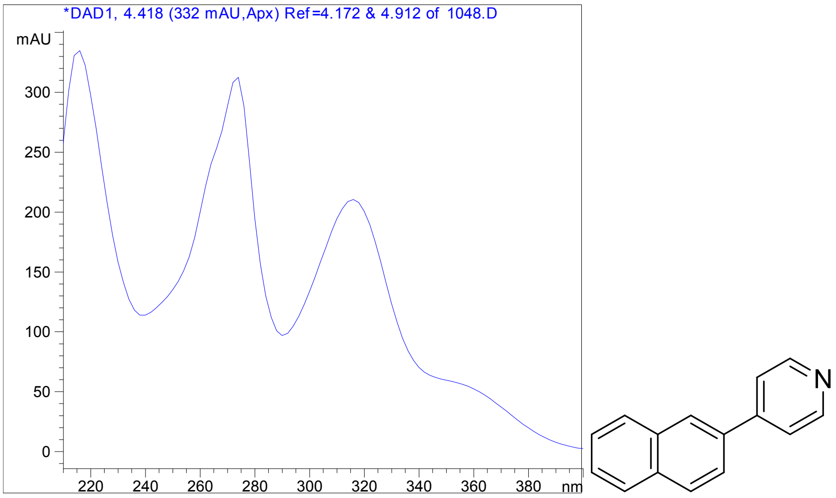

Fig. S10: UV spectrum of 6-(naphthalen-2-yl)pyridazin-3(2*H*)-one (11a)

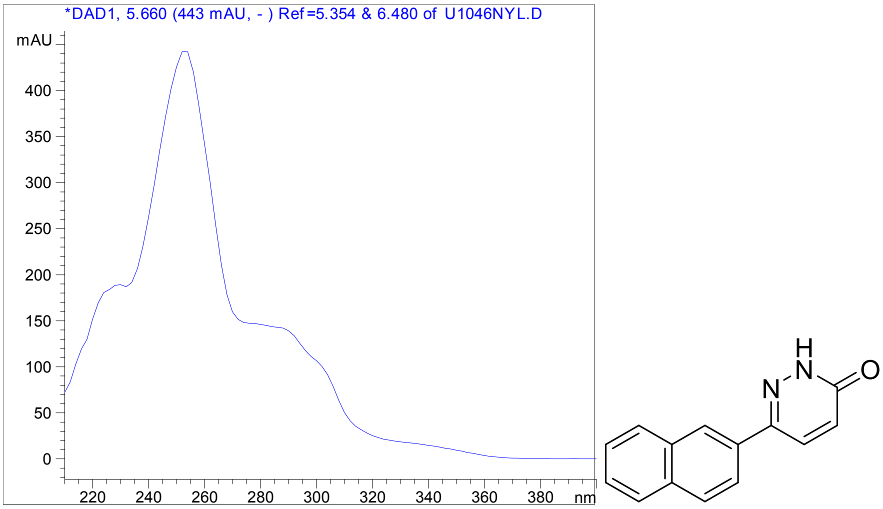

Fig. S11: UV spectrum of 2-(2-hydroxypropyl)-6-(naphthalen-2-yl)pyridazin-3(2*H*)-one (11b)

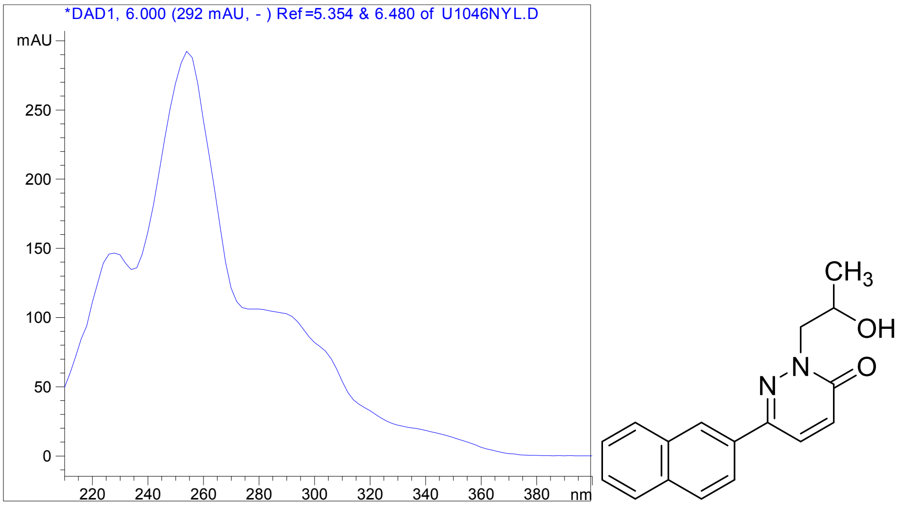

Fig. S12: UV spectrum of 2-phenylpyridine (12)

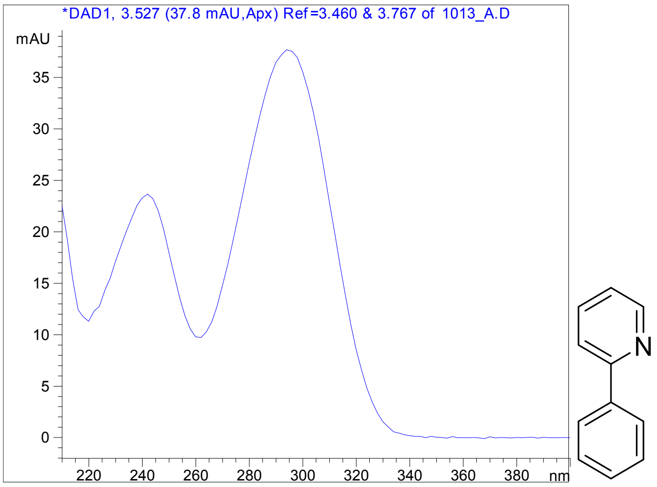

Fig. S13: UV spectrum of 4-phenylpyridine (13)

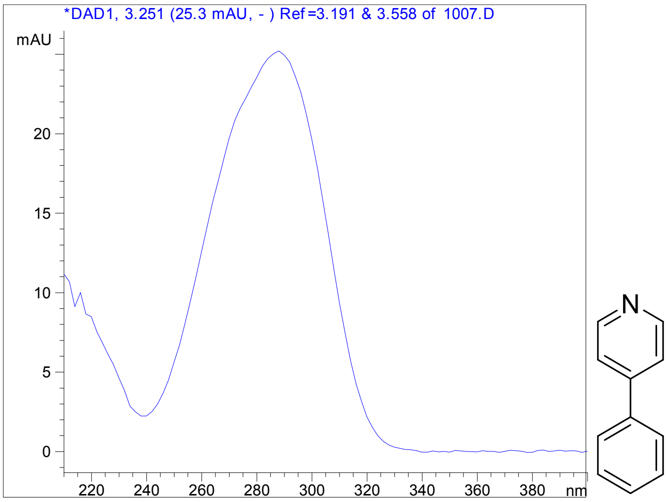

Fig. S14: UV spectrum of 2-(biphenyl-4-yl)pyridine (14)

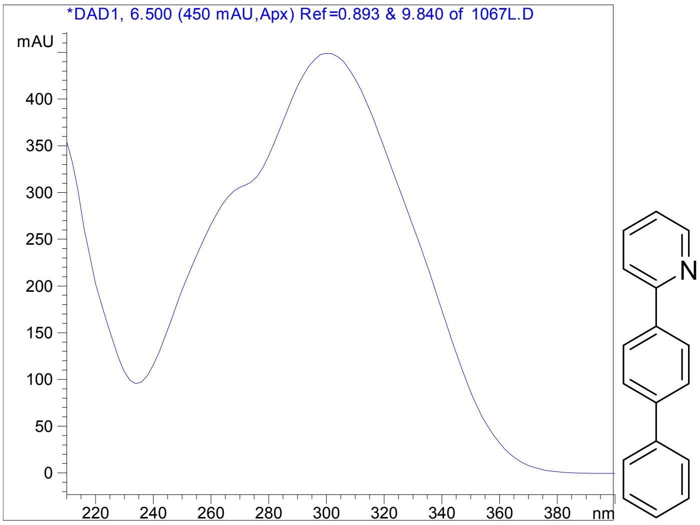

Fig. S15: UV spectrum of 4-(biphenyl-4-yl)pyridine (15)

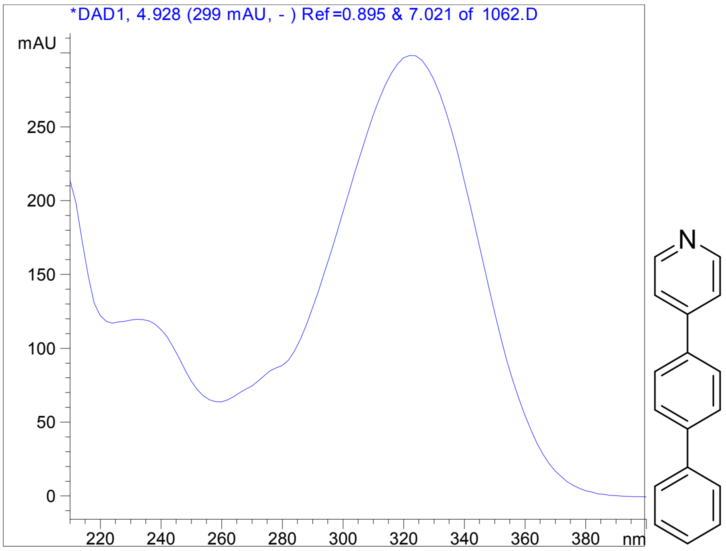

Fig. S16: UV spectrum of 2-(4-fluorophenyl)pyridine (16)

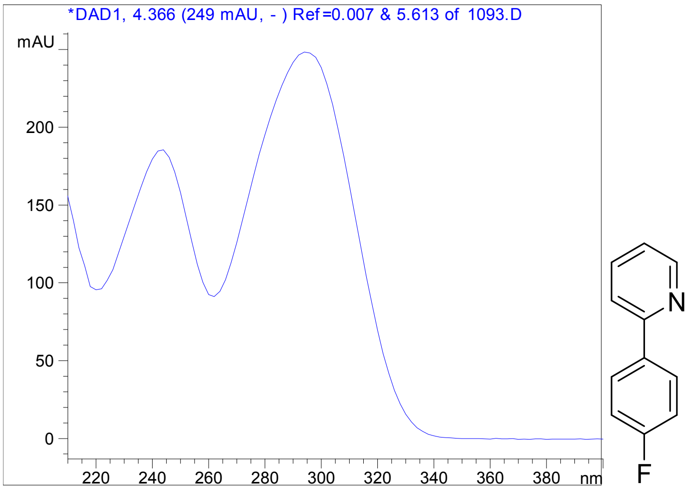

Fig. S17: UV spectrum of 4-(4-fluorophenyl)pyridine (17)

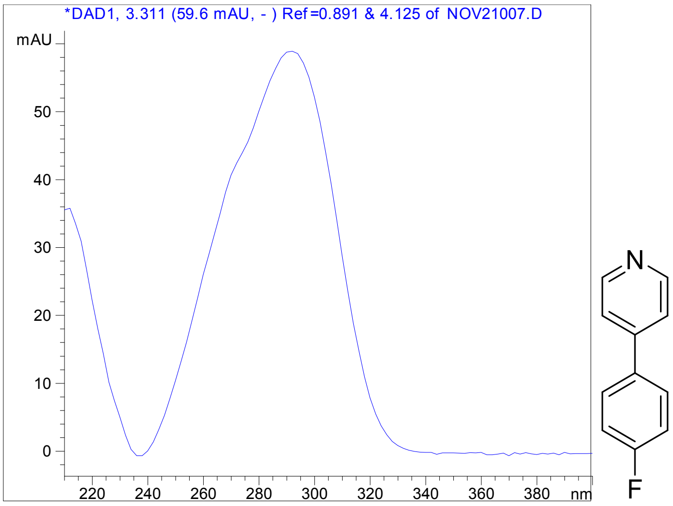

Fig. S18: UV spectrum of 6-phenylpyridazin-3(2*H*)-one (18a)

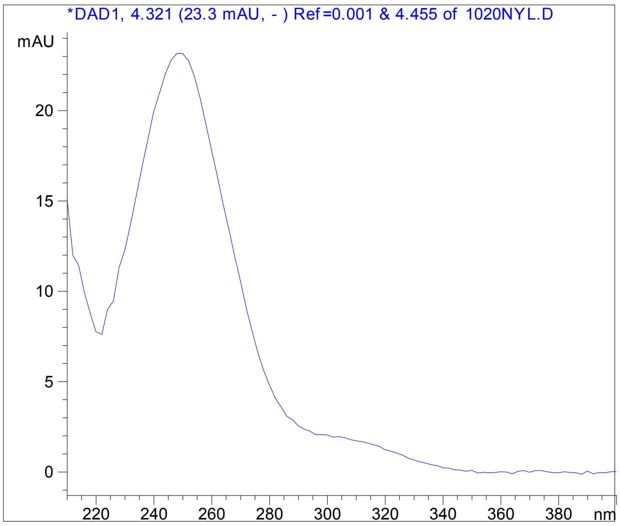

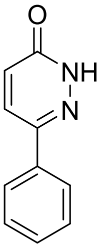

Fig. S19: UV spectrum of 2-(2-hydroxypropyl)-6-phenylpyridazin-3(2*H*)-one (18b)

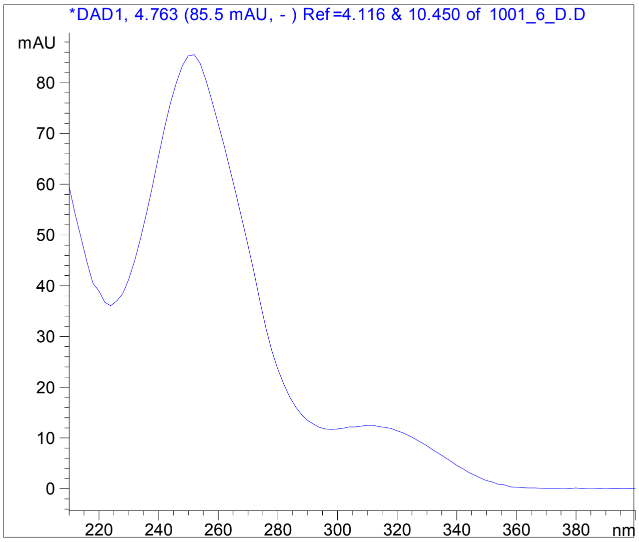

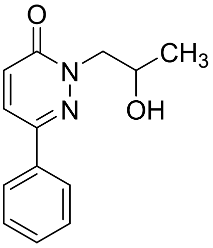

Fig. S20: UV spectrum of 6-(biphenyl-4-yl)pyridazin-3(2*H*)-one (19a)

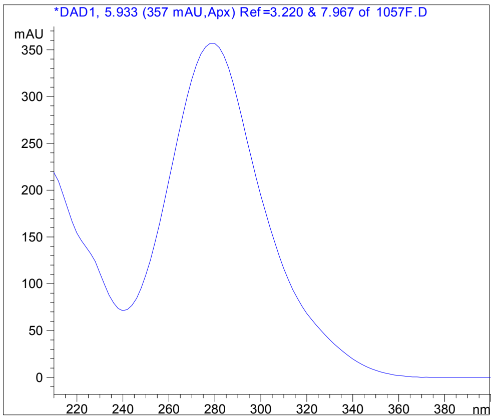

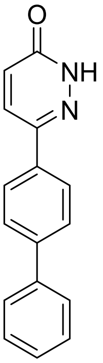

Fig. S21: UV spectrum of 6-(biphenyl-4-yl)-2-(2-hydroxypropyl)-pyridazin-3(2*H*)-one (19b)

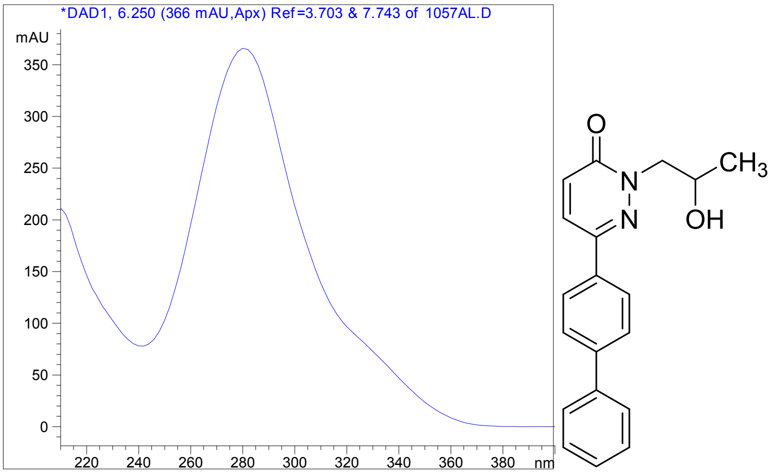

Fig. S22: UV spectrum of 6-(4-fluorophenyl)pyridazin-3(2*H*)-one (20a)

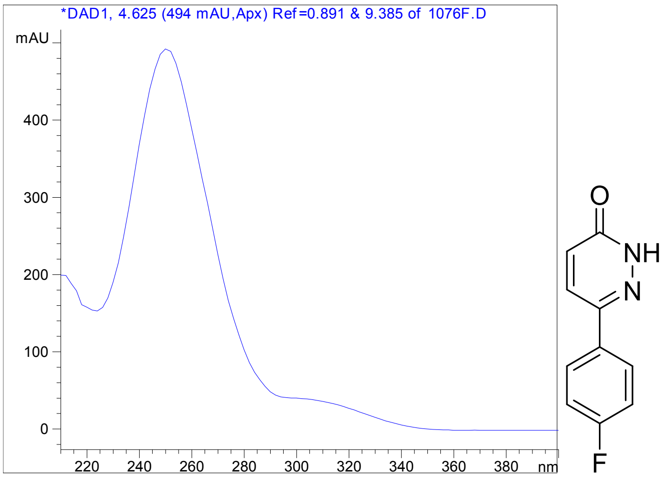

Fig. S23: 6-(4-fluorophenyl)-2-(2-hydroxypropyl)pyridazin-3(2*H*)-one (20b)

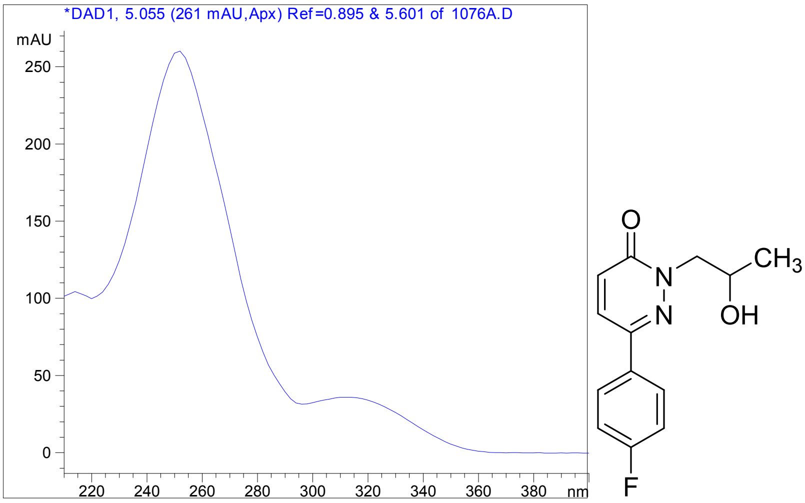

Fig. S24: 2-(2-hydroxypropyl)-6-iodopyridazin-3(2*H*)-one (23)

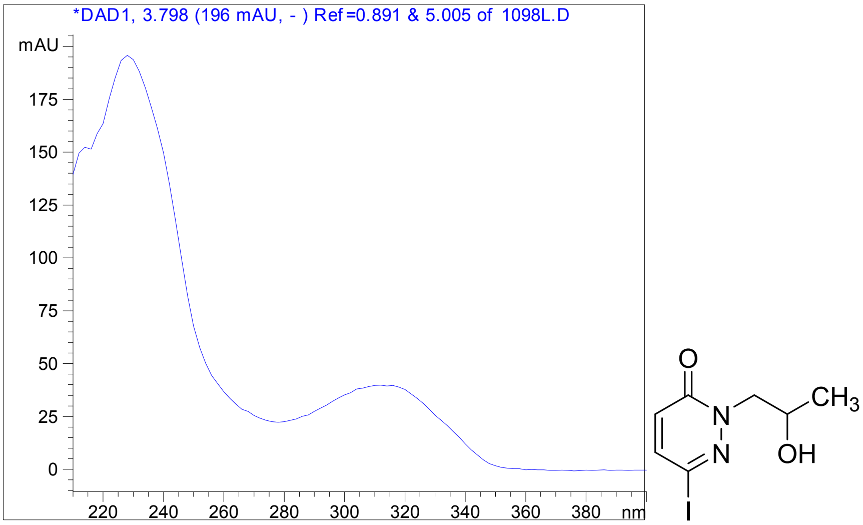

Fig. S25. HPLC-MS data for 3,6-diiodopyridazine:

Sample Information

Acquired by	: System Administrator
Sample Name	: CSA1099_AT1
Sample ID	:
Tray#	: 1
Vail#	: 18
Injection Volume	: 3 uL
Data Filename	: CSA1099_AT1.lcd
Method Filename	: A_5_100_1ml_13min.lcm
Batch Filename	: 20190318.lcb
Report Filename	: DEFAULT.lsr
Date Acquired	: 2019.03.18. 16:11:46
Data Processed	: 2019.03.20. 10:36:55

Chromatogram
CSA1099_AT1 C:\LabSolutions\Data\Project1\DR\20190318\CSA1099_AT1.lcd

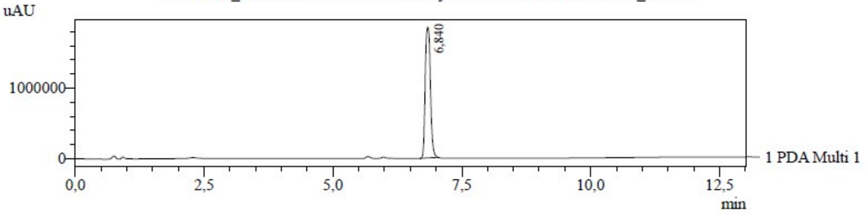

1 PDA Multi 1 / 254nm,4nm

PeakTable C:\LabSolutions\Data\Project1\DR\20190318\CSA1099_AT1.lcd

PDA Ch1 254nm

Peak#	Ret. Time	Area	Height	Area %	Height %
1	6,840	12997726	1848301	100,000	100,000
Total		12997726	1848301	100,000	100,000

MS Spectrum

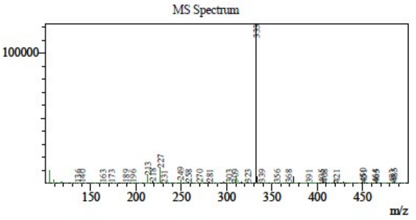

Fig. S26. HPLC-MS data for 6-iodopyridazine-3(2*H*)-one (4):

Sample Information

Acquired by	: System Administrator
Sample Name	: CSA1100_AT1
Sample ID	:
Tray#	: 1
Vail#	: 14
Injection Volume	: 3 uL
Data Filename	: CSA1100_AT1.lcd
Method Filename	: A_5_100_1ml_13min.lcm
Batch Filename	: 20190318.lcb
Report Filename	: DEFAULT.lsr
Date Acquired	: 2019.03.18. 15:13:47
Data Processed	: 2019.03.20. 10:39:59

Chromatogram
CSA1100_AT1 C:\LabSolutions\Data\Project1\DR\20190318\CSA1100_AT1.lcd

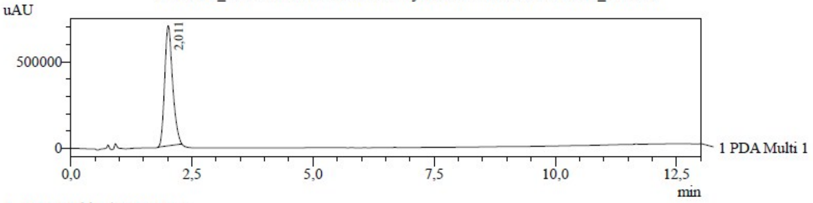

1 PDA Multi 1 / 254nm,4nm

PeakTable C:\LabSolutions\Data\Project1\DR\20190318\CSA1100_AT1.lcd

PDA Ch1 254nm

Peak#	Ret. Time	Area	Height	Area %	Height %
1	2,011	7901918	692280	100,000	100,000
Total		7901918	692280	100,000	100,000

MS Spectrum

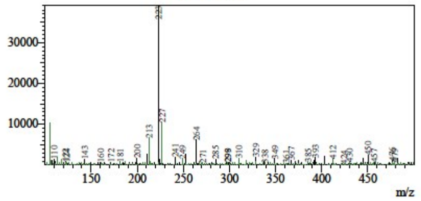

Fig. S27. HPLC-MS data for purified 2-(naphthalen-2-yl)pyridine (9):

Sample Information

Acquired by	: System Administrator
Date Acquired	: 2018.09.05. 8:49:38
Sample Name	: CSA1053_AT1
Vial#	: 18
Injection Volume	: 1
Data File	: CSA1053_AT1.lcd
Method File	: A_5_100_1ml_13min.lcm
Tuning File	: Tuning_20170803.lct

Chromatogram

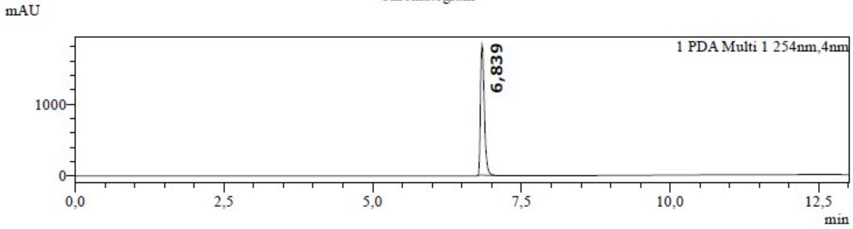

Peak Table

PDA Ch1 254nm

Peak#	Ret. Time	Area	Height	Area%
1	6,839	8148202	1818844	100,000
Total		8148202	1818844	100,000

MS Spectrum

R.Time:6,867(Scan#:413)

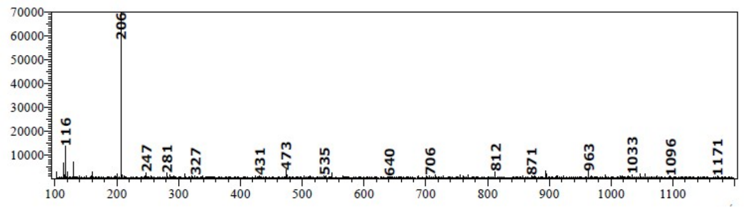

Fig S28. HPLC-MS data for 2-(naphthalen-2-yl)pyridine (9) by Method A:

Sample Information

Acquired by	: System Administrator
Date Acquired	: 2018.09.03. 10:43:26
Sample Name	: CSA1055_ny_lio_A
Vial#	: 103
Injection Volume	: 1
Data File	: CSA1055_ny_lio_A.lcd
Method File	: A_5_100_1ml_13min.lcm
Tuning File	: Tuning_20170803.lct

Chromatogram

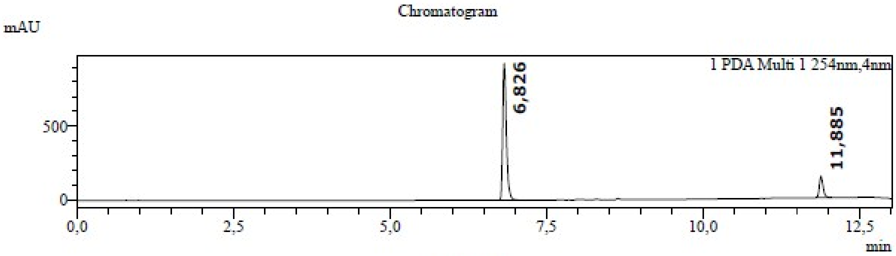

Peak Table

PDA Ch1 254nm

Peak#	Ret. Time	Area	Height	Area%
1	6.826	3676004	923867	86.526
2	11.885	572431	140040	13.474
Total		4248435	1063907	100.000

MS Spectrum

Peak#:1 R.Time:6,897(Scan#:413)

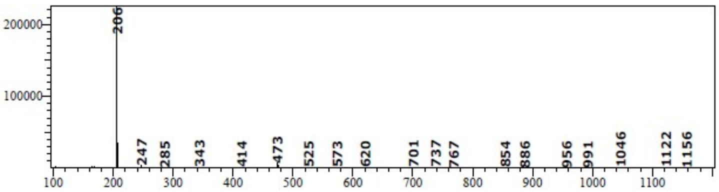

Fig. S29. HPLC-MS data for 2-(naphthalen-2-yl)pyridine (9) by Method B:

Sample Information

Acquired by	: System Administrator
Date Acquired	: 2018.09.03. 11:52:52
Sample Name	: CSA1056_ny_lio_A
Vial#	: 105
Injection Volume	: 1
Data File	: CSA1056_ny_lio_A.lcd
Method File	: A_5_100_1ml_13min.lcm
Tuning File	: Tuning_20170803.lct

Chromatogram

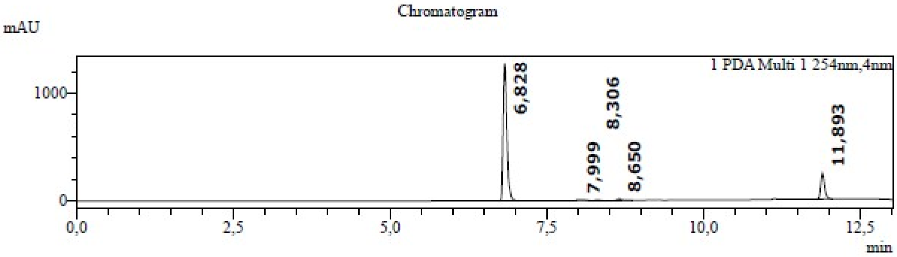

Peak Table

PDA Ch1 254nm

Peak#	Ret. Time	Area	Height	Area%
1	6.828	5163014	1275924	82.262
2	7.999	13998	2250	0.223
3	8.306	28006	7107	0.446
4	8.650	71303	16948	1.136
5	11.893	999961	240589	15.932
Total		6276283	1542818	100.000

MS Spectrum

Peak#:1 R.Time:6,900(Scan#:415)

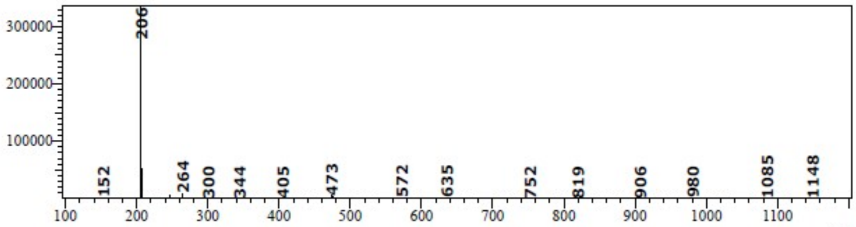

Fig. S30. HPLC-MS data for purified 4-(naphthalen-2-yl)pyridine (10):

Sample Information

Acquired by	: System Administrator
Date Acquired	: 2018.09.05. 9:10:31
Sample Name	: CSA1050A_lio_AT1
Vial#	: 20
Injection Volume	: 1
Data File	: CSA1050A_lio_AT1.lcd
Method File	: A_5_100_1ml_13min.lcm
Tuning File	: Tuning_20170803.lct

Chromatogram

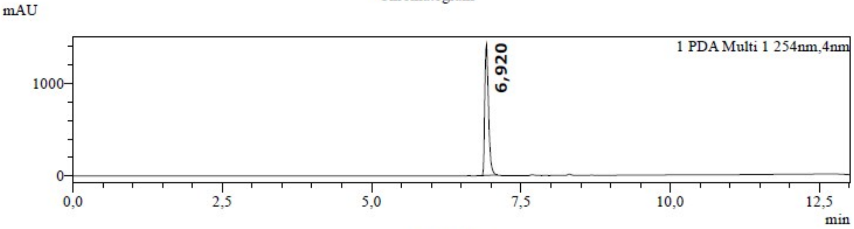

Peak Table

PDA Ch1 254nm

Peak#	Ret. Time	Area	Height	Area%
1	6,920	6116984	1412987	100,000
Total		6116984	1412987	100,000

MS Spectrum

R.Time:6,900(Scan#:415)

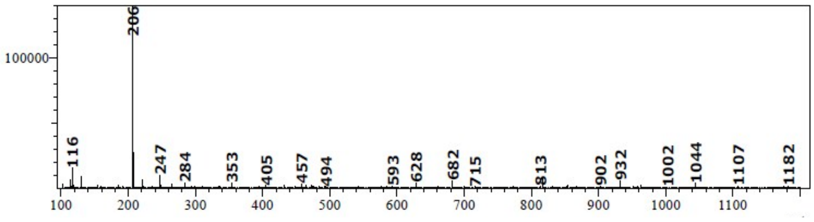

Fig. S31. HPLC-MS data for 4-(naphthalen-2-yl)pyridine (10) by Method A:

Sample Information

Acquired by	: System Administrator
Date Acquired	: 2018.09.03. 10:04:44
Sample Name	: CSA1052_ny_A
Vial#	: 101
Injection Volume	: 2
Data File	: CSA1052_ny_A.lcd
Method File	: A_5_100_1ml_13min.lcm
Tuning File	: Tuning_20170803.lct

Chromatogram

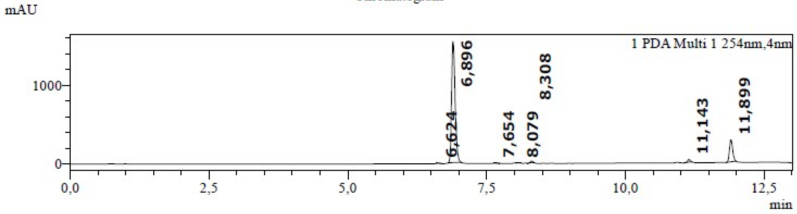

Peak Table

PDA Ch1 254nm

Peak#	Ret. Time	Area	Height	Area%
1	6,624	9741	5253	0,120
2	6,896	6813298	1533189	83,789
3	7,654	18155	6277	0,223
4	8,079	11612	5410	0,143
5	8,308	50722	18690	0,624
6	11,143	104847	31483	1,289

MS Spectrum

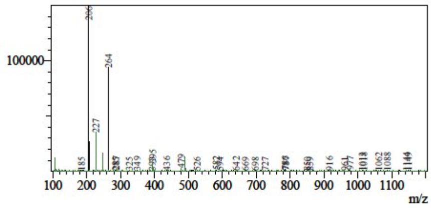

Fig. S32. HPLC-MS data for 4-(naphthalen-2-yl)pyridine (10) by Method B:

Sample Information

Acquired by	: System Administrator
Date Acquired	: 2018.09.03. 8:44:21
Sample Name	: CSA1051_ny_lio_A
Vial#	: 97
Injection Volume	: 1
Data File	: CSA1051_ny_lio_A.lcd
Method File	: A_5_100_1ml_13min.lcm
Tuning File	: Tuning_20170803.lct

Chromatogram

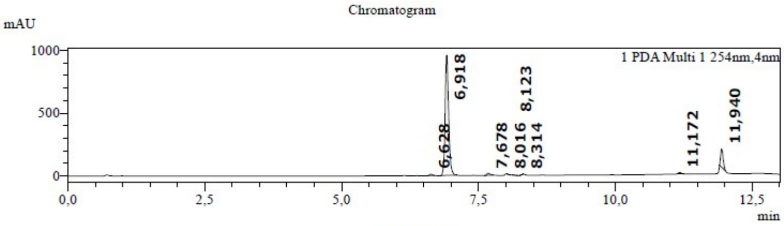

Peak Table

PDA Ch1 254nm

Peak#	Ret. Time	Area	Height	Area%
1	6,628	36208	10489	0,760
2	6,918	4192764	955398	88,017
3	7,678	67852	15961	1,424
4	8,016	-6657	761	-0,140
5	8,123	-6785	255	-0,142
6	8,314	5480	1071	0,115

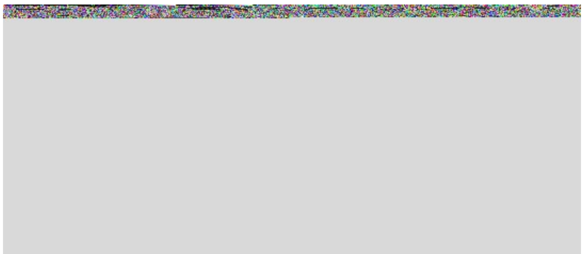

Fig. S33. HPLC-MS data for purified 6-(naphthalen-2-yl)pyridazin-3(2*H*)-one (11a):

Sample Information

Acquired by	: System Administrator
Date Acquired	: 2018.08.29. 13:23:45
Sample Name	: CSA1040_sz_AT1
Vial#	: 68
Injection Volume	: 1
Data File	: CSA1040_sz_AT1.lcd
Method File	: A_5_100_1ml_13min.lcm
Tuning File	: Tuning_20170803.lct

Chromatogram

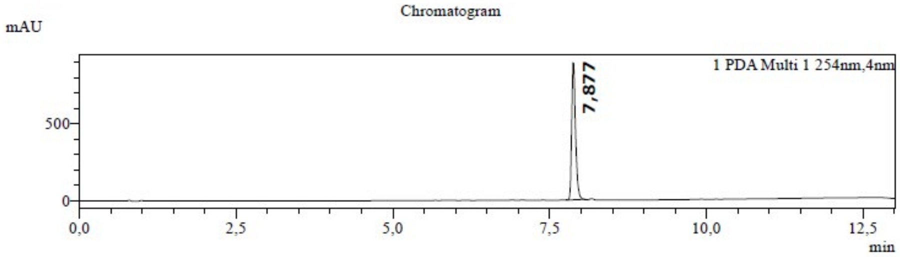

Peak Table

PDA Ch1 254nm

Peak#	Ret. Time	Area	Height	Area%
1	7,877	3819600	890788	100,000
Total		3819600	890788	100,000

MS Spectrum

R.Time:7,867(Scan#:473)

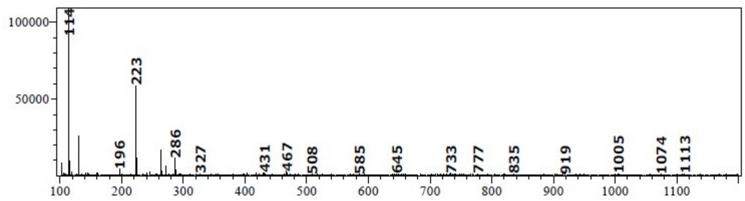

Fig. S34. HPLC-MS data for 6-(naphthalen-2-yl)pyridazin-3(2*H*)-one (11a) by Method A:

Sample Information

Acquired by	: System Administrator
Date Acquired	: 2018.06.27. 13:43:00
Sample Name	: CSA1034_F_AT1
Vial#	: 83
Injection Volume	: 2
Data File	: CSA1034_F_AT1.lcd
Method File	: A_5_100_1ml_13min.lcm
Tuning File	: Tuning_20170803.lct

Chromatogram

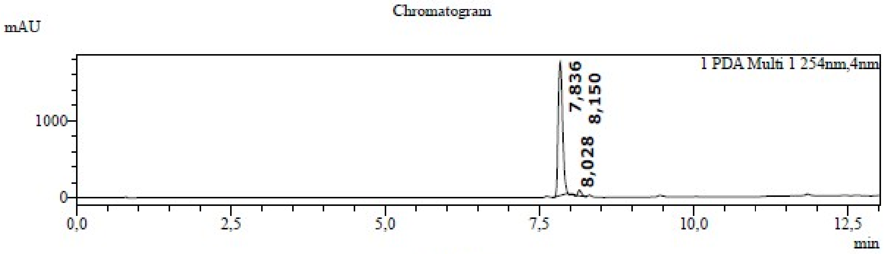

Peak Table

PDA Ch1 254nm

Peak#	Ret. Time	Area	Height	Area%
1	7.836	8393464	1733731	97.109
2	8.028	6734	10025	0.078
3	8.150	243153	72906	2.813
Total		8643350	1816663	100.000

Fig. S35. HPLC-MS data for 6-(naphthalen-2-yl)pyridazin-3(2*H*)-one (11a) by Method B:

Sample Information

Acquired by	: System Administrator
Sample Name	: CSA1090F_AT1
Sample ID	:
Tray#	: 1
Vail#	: 99
Injection Volume	: 2 uL
Data Filename	: CSA1090F_AT1.lcd
Method Filename	: A_5_100_1ml_13min.lcm
Batch Filename	:
Report Filename	: Default.lsr
Date Acquired	: 2019.01.29. 11:20:53
Data Processed	: 2019.01.29. 12:09:36

Chromatogram
CSA1090F_AT1 C:\LabSolutions\Data\Project1\DR\CSA5\CSA1090F_AT1.lcd

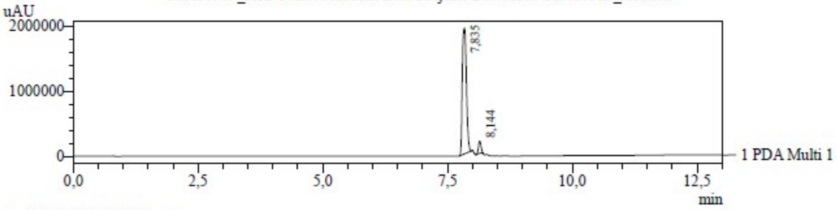

1 PDA Multi 1 / 254nm,4nm

PeakTable C:\LabSolutions\Data\Project1\DR\CSA5\CSA1090F_AT1.lcd

PDA Ch1 254nm

Peak#	Ret. Time	Area	Height	Area %	Height %
1	7,835	11165980	1927886	94,580	91,073
2	8,144	639890	188982	5,420	8,927
Total		11805870	2116868	100,000	100,000

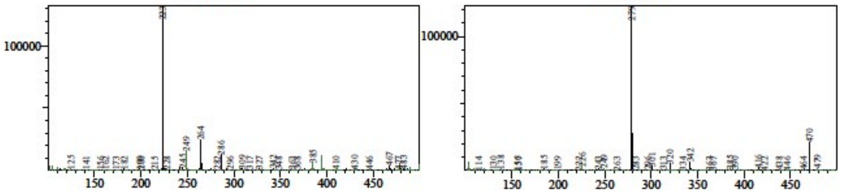

Fig. S36. HPLC-MS data for purified 2-(2-hydroxypropyl)-6-(naphthalen-2-yl)pyri-dazin-3(2*H*)-one (11b):

Sample Information

Acquired by	: System Administrator
Sample Name	: CSA1040A_AT1
Sample ID	:
Tray#	: 1
Vail#	: 29
Injection Volume	: 2 uL
Data Filename	: CSA1040A_AT1.lcd
Method Filename	: A_5_100_1ml_13min.lcm
Batch Filename	:
Report Filename	: Default.lsr
Date Acquired	: 2018.12.06. 10:16:15
Data Processed	: 2018.12.06. 10:57:43

Chromatogram
CSA1040A_AT1 C:\LabSolutions\Data\Project1\DR\CSA3\CSA1040A_AT1.lcd

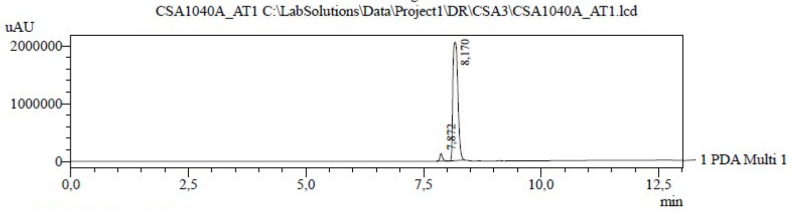

1 PDA Multi 1 / 254nm,4nm

PeakTable C:\LabSolutions\Data\Project1\DR\CSA3\CSA1040A_AT1.lcd

PDA Ch1 254nm

Peak#	Ret. Time	Area	Height	Area %	Height %
1	7,872	521948	129027	3,325	5,932
2	8,170	15175429	2046193	96,675	94,068
Total		15697377	2175220	100,000	100,000

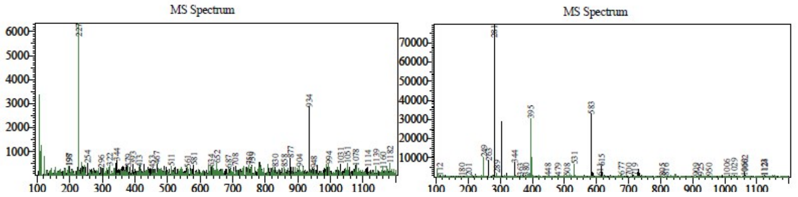

Fig. S37. HPLC-MS data for 2-(2-hydroxypropyl)-6-(naphthalen-2-yl)pyridazin-3(2*H*)-one (11b) by Method A:

Sample Information

Acquired by	: System Administrator
Date Acquired	: 2018.06.26. 10:06:12
Sample Name	: CSA1034A_AT1
Vial#	: 64
Injection Volume	: 2
Data File	: CSA1034A_AT1.lcd
Method File	: A_5_100_1ml_13min.lcm
Tuning File	: Tuning_20170803.lct

Chromatogram

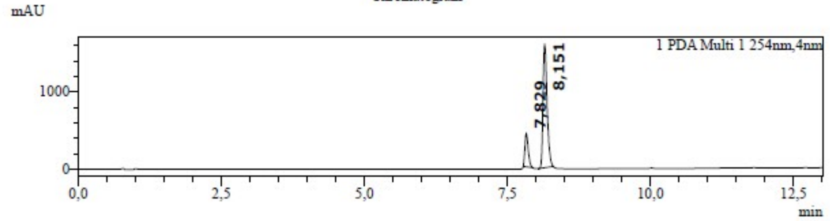

Peak Table

PDA Ch1 254nm

Peak#	Ret. Time	Area	Height	Area%
1	7.829	1721695	428355	17.862
2	8.151	7916983	1608233	82.138
Total		9638677	2036588	100.000

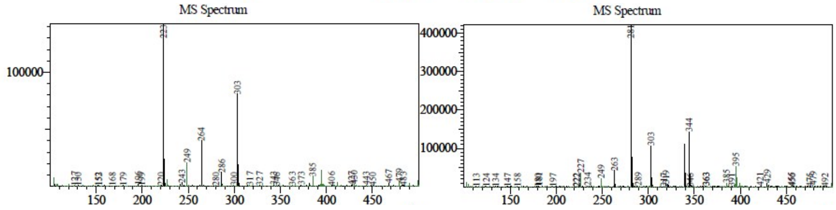

Fig. S38. HPLC-MS data for 2-(2-hydroxypropyl)-6-(naphthalen-2-yl)pyridazin-3(2*H*)-one (11b) by Method B:

Sample Information

Acquired by	: System Administrator
Sample Name	: CSA1090A_AT1
Sample ID	:
Tray#	: 1
Vail#	: 3
Injection Volume	: 2 uL
Data Filename	: CSA1090A_AT1.lcd
Method Filename	: A_5_100_1ml_13min.lcm
Batch Filename	:
Report Filename	: Default.lsr
Date Acquired	: 2019.01.29. 14:16:29
Data Processed	: 2019.01.29. 14:30:36

Chromatogram

CSA1090A_AT1 C:\LabSolutions\Data\Project1\DR\CSA5\CSA1090A_AT1.lcd

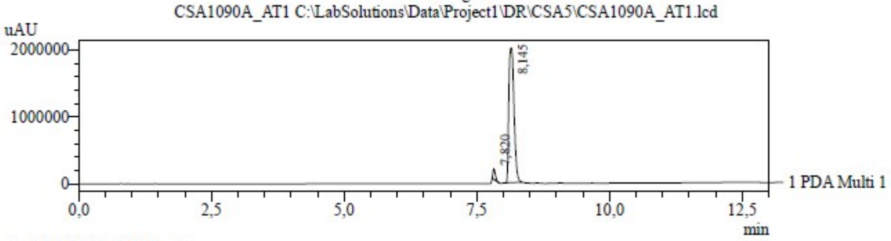

1 PDA Multi 1 / 254nm,4nm

PeakTable C:\LabSolutions\Data\Project1\DR\CSA5\CSA1090A_AT1.lcd

PDA Ch1 254nm

Peak#	Ret. Time	Area	Height	Area %	Height %
1	7,820	471020	156504	3,355	7,222
2	8,145	13569351	2010492	96,645	92,778
Total		14040371	2166996	100,000	100,000

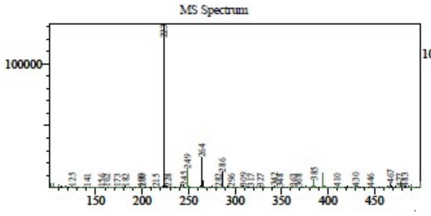

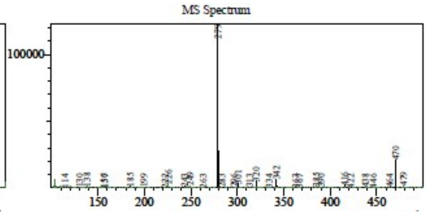

Fig. S39. HPLC-MS data for 6-(naphthalen-2-yl)pyridazin-3(2*H*)-one (11a) and 2-(2-hydroxypropyl)-6-(naphthalen-2-yl)pyridazin-3(2*H*)-one (11b) by Method B:

Sample Information

Acquired by	: System Administrator
Date Acquired	: 2018.06.27. 16:33:22
Sample Name	: CSA1046_AT1
Vial#	: 88
Injection Volume	: 2
Data File	: CSA1046_AT1.lcd
Method File	: A_5_100_1ml_13min.lcm
Tuning File	: Tuning_20170803.lct

Chromatogram

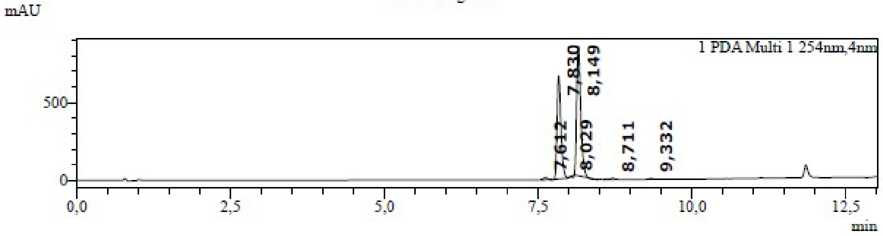

Peak Table

PDA Ch1 254nm

Peak#	Ret. Time	Area	Height	Area%
1	7.612	63747	14750	0.952
2	7.830	2851254	665957	42.578
3	8.029	-16175	1203	-0.242
4	8.149	3727757	835894	55.667
5	8.711	43020	7675	0.642
6	9.332	26931	6269	0.402
Total		6696534	1531748	100.000

Fig. S40. HPLC-MS data for purified 2-phenylpyridine (12):

Sample Information

Acquired by	: System Administrator
Sample Name	: CzA_1013
Sample ID	:
Tray#	: 1
Vail#	: 53
Injection Volume	: 3 uL
Data Filename	: CzA_1013.lcd
Method Filename	: A_5_100_1ml_13min.lcm
Batch Filename	: Batch03_re.lcb
Report Filename	: DEFAULT.lsr
Date Acquired	: 2018.01.30. 13:06:19
Data Processed	: 2018.02.09. 10:03:26

Chromatogram
CzA_1013 C:\LabSolutions\Data\Project1\DR\CzA_1013.lcd

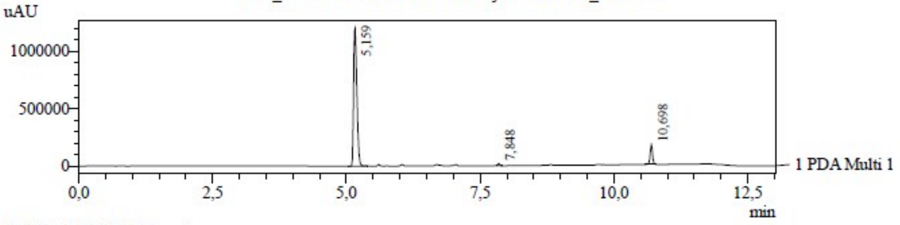

1 PDA Multi 1 / 254nm,4nm

PeakTable C:\LabSolutions\Data\Project1\DR\CzA_1013.lcd

PDA Ch1 254nm

Peak#	Ret. Time	Area	Height	Area %	Height %
1	5,159	5200988	1193627	89,968	86,329
2	7,848	50704	18150	0,877	1,313
3	10,698	529218	170865	9,155	12,358
Total		5780910	1382642	100,000	100,000

MS Spectrum

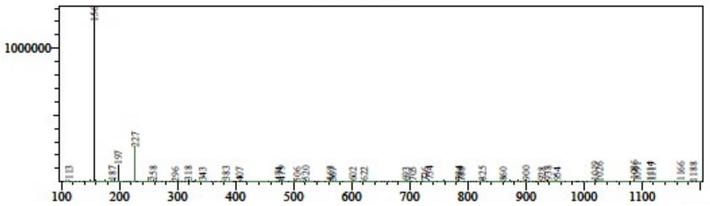

Fig. S41. HPLC-MS data for 2-phenylpyridine (12) by Method A:

Sample Information

Acquired by	: System Administrator
Date Acquired	: 2018.06.26. 12:27:08
Sample Name	: CSA1047_lio_AT1
Vial#	: 70
Injection Volume	: 2
Data File	: CSA1047_lio_AT1.lcd
Method File	: A_5_100_1ml_13min.lcm
Tuning File	: Tuning_20170803.lct

Chromatogram

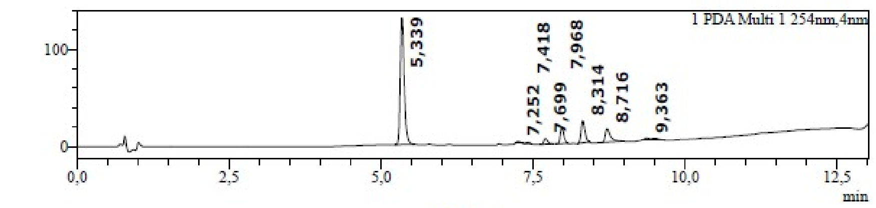

Peak Table

PDA Ch1 254nm

Peak#	Ret. Time	Area	Height	Area%
1	5,339	604271	130364	66,162
2	7,252	5134	1357	0,562
3	7,418	3908	1398	0,428
4	7,699	24058	5873	2,634
5	7,968	69144	16986	7,571
6	8,314	97357	22699	10,660

UV Spectrum

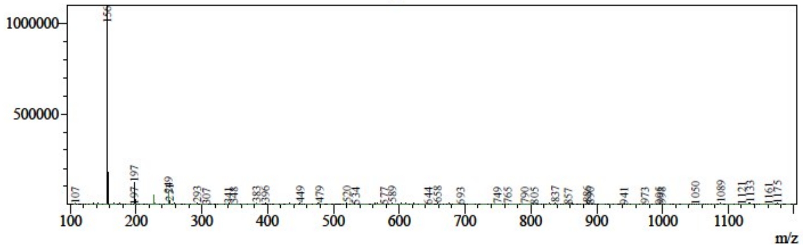

Fig. S42. HPLC-MS data for purified 2-phenylpyridine (12):

Sample Information

Acquired by	: System Administrator
Sample Name	: CSA1089_AT1
Sample ID	:
Tray#	: 1
Vail#	: 90
Injection Volume	: 2 uL
Data Filename	: CSA1089_AT1.lcd
Method Filename	: A_5_100_1ml_13min.lcm
Batch Filename	:
Report Filename	: Default.lsr
Date Acquired	: 2019.01.29. 8:54:33
Data Processed	: 2019.01.29. 12:00:59

Chromatogram

CSA1089_AT1 C:\LabSolutions\Data\Project1\DR\CSA5\CSA1089_AT1.lcd

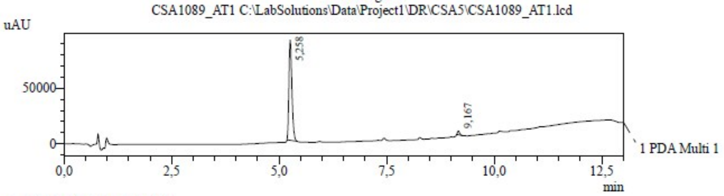

1 PDA Multi 1 / 254nm,4nm

PeakTable C:\LabSolutions\Data\Project1\DR\CSA5\CSA1089_AT1.lcd

PDA Ch1 254nm

Peak#	Ret. Time	Area	Height	Area %	Height %
1	5,258	432166	90667	97,526	96,441
2	9,167	10964	3346	2,474	3,559
Total		443130	94013	100,000	100,000

MS Spectrum

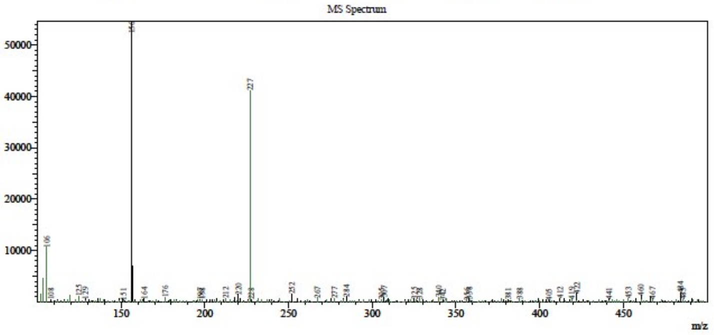

Fig. S43. HPLC-MS data for 2-phenylpyridine (12) by Method B:

Sample Information

Acquired by	: System Administrator
Date Acquired	: 2018.06.27. 13:23:35
Sample Name	: CSA1047_nyers_AT1
Vial#	: 83
Injection Volume	: 2
Data File	: CSA1047_nyers_AT1.lcd
Method File	: A_5_100_1ml_13min.lcm
Tuning File	: Tuning_20170803.lct

Chromatogram

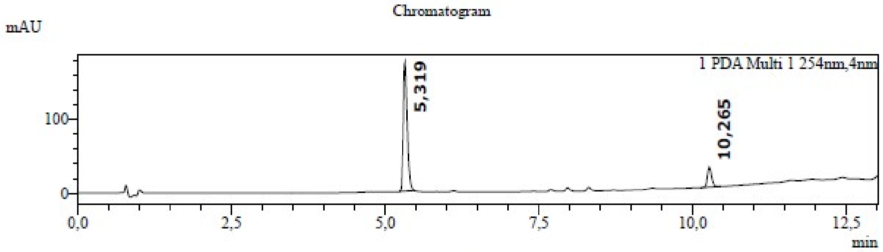

Peak Table

PDA Ch1 254nm

Peak#	Ret. Time	Area	Height	Area%
1	5.319	808691	175968	86.453
2	10.265	126722	27139	13.547
Total		935412	203108	100.000

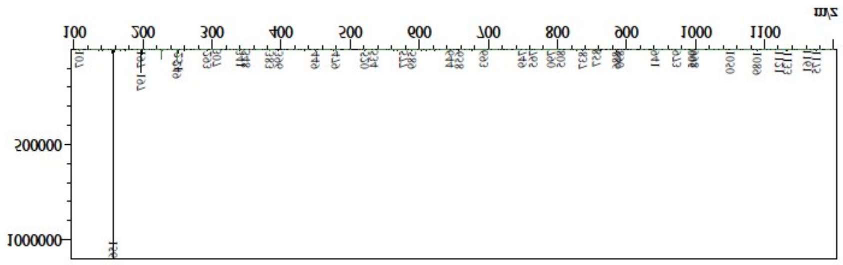

Fig. S44. HPLC-MS data for purified 4-phenylpyridine (13):

Sample Information

Acquired by	: System Administrator
Sample Name	: CzA_1007_lio
Sample ID	:
Tray#	: 1
Vail#	: 57
Injection Volume	: 3 uL
Data Filename	: CzA_1007_lio.lcd
Method Filename	: A_5_100_1ml_13min.lcm
Batch Filename	: Batch03_re.lcb
Report Filename	: DEFAULT.lsr
Date Acquired	: 2018.01.30. 14:01:11
Data Processed	: 2018.02.09. 10:07:24

Chromatogram
CzA_1007_lio C:\LabSolutions\Data\Project1\DR\CzA_1007_lio.lcd

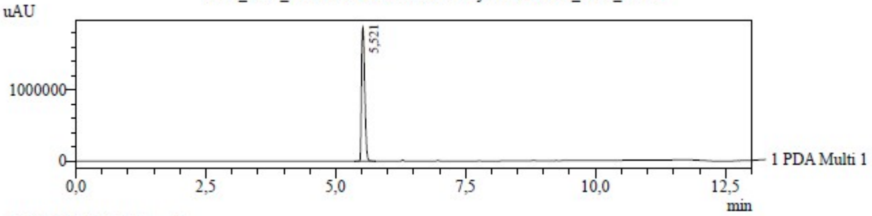

1 PDA Multi 1 / 254nm,4nm

PeakTable C:\LabSolutions\Data\Project1\DR\CzA_1007_lio.lcd

PDA Ch1 254nm

Peak#	Ret. Time	Area	Height	Area %	Height %
1	5,521	8168449	1866732	100,000	100,000
Total		8168449	1866732	100,000	100,000

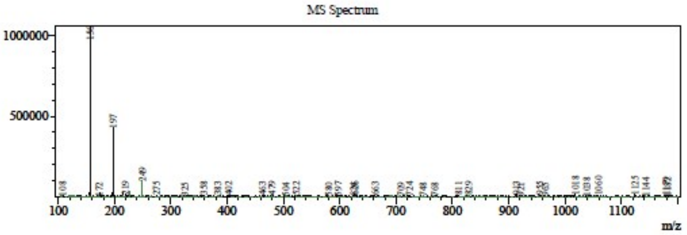

Fig. S45. HPLC-MS data for 4-phenylpyridine (13) by Method A:

Sample Information

Acquired by	: System Administrator
Sample Name	: CzA_1011ny_lio2
Sample ID	:
Tray#	: 1
Vail#	: 56
Injection Volume	: 3 uL
Data Filename	: CzA_1011ny_lio2.lcd
Method Filename	: A_5_100_1ml_13min.lcm
Batch Filename	: Batch03_re.lcb
Report Filename	: DEFAULT.lsr
Date Acquired	: 2018.01.30. 13:47:28
Data Processed	: 2018.02.09. 10:32:38

Chromatogram

CzA_1011ny_lio2 C:\LabSolutions\Data\Project1\DR\CzA_1011ny_lio2.lcd

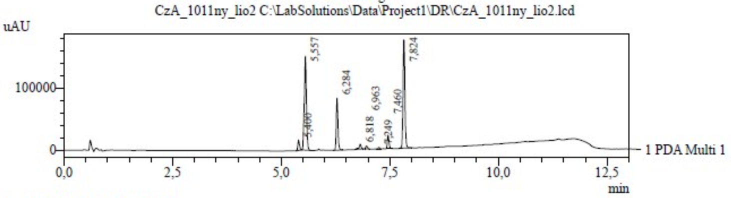

1 PDA Multi 1 / 254nm,4nm

PeakTable C:\LabSolutions\Data\Project1\DR\CzA_1011ny_lio2.lcd

PDA Ch1 254nm

Peak#	Ret. Time	Area	Height	Area %	Height %
1	5,400	45429	16330	3,169	3,518
2	5,557	527184	152710	36,774	32,895
3	6,284	223223	84695	15,571	18,244
4	6,818	25968	7565	1,811	1,629
5	6,963	13303	4620	0,928	0,995
6	7,249	5275	2129	0,368	0,459
7	7,460	60335	21665	4,209	4,667
8	7,824	532868	174514	37,170	37,592
Total		1433586	464228	100,000	100,000

MS Spectrum

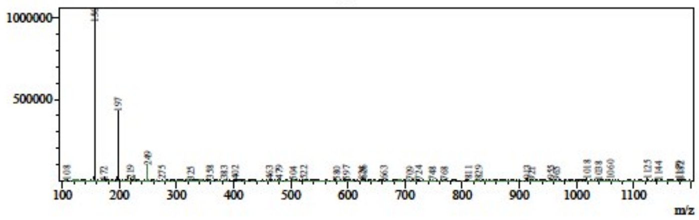

Fig. S46. HPLC-MS data for 4-phenylpyridine (13) by Method B:

Sample Information

Acquired by	: System Administrator
Sample Name	: CsA1072_ny_lio_AT1
Sample ID	:
Tray#	: 1
Vail#	: 72
Injection Volume	: 2 uL
Data Filename	: CsA1072_ny_lio_AT1.lcd
Method Filename	: A_5_100_1ml_13min.lcm
Batch Filename	:
Report Filename	: Default.lsr
Date Acquired	: 2018.12.07. 8:58:53
Data Processed	: 2018.12.07. 9:22:18

Chromatogram

CsA1072_ny_lio_AT1 C:\LabSolutions\Data\Project1\DR\CSA3\CsA1072_ny_lio_AT1.lcd

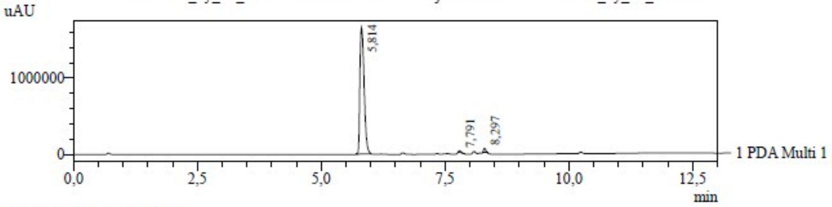

1 PDA Multi 1 / 254nm,4nm

PeakTable C:\LabSolutions\Data\Project1\DR\CSA3\CsA1072_ny_lio_AT1.lcd

PDA Ch1 254nm

Peak#	Ret. Time	Area	Height	Area %	Height %
1	5,814	9754162	1647208	97,603	95,548
2	7,791	61003	19138	0,610	1,110
3	8,297	178550	57620	1,787	3,342
Total		9993715	1723966	100,000	100,000

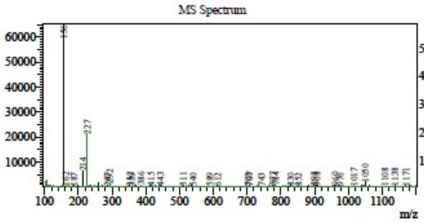

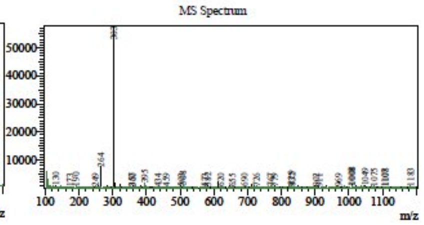

Fig. S47. HPLC-MS data for 4-phenylpyridine (13) by Method B:

Sample Information

Acquired by	: System Administrator
Date Acquired	: 2018.06.27. 16:16:14
Sample Name	: CSA1043_lio_AT1
Vial#	: 85
Injection Volume	: 2
Data File	: CSA1043_lio_AT1.lcd
Method File	: A_5_100_1ml_13min.lcm
Tuning File	: Tuning_20170803.lct

Chromatogram

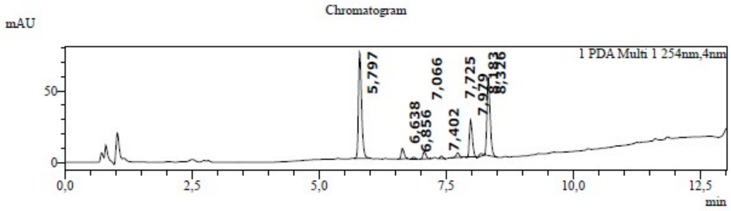

Peak Table

PDA Ch1 254nm

Peak#	Ret. Time	Area	Height	Area%
1	5,797	316768	74098	42,576
2	6,638	29480	7296	3,962
3	6,856	3970	1098	0,534
4	7,066	20679	5086	2,779
5	7,402	5864	1691	0,788
6	7,725	13743	3239	1,847
7	7,979	106368	25959	14,296
8	8,183	9331	1746	1,254
9	8,326	237810	54210	31,963
Total		744012	174423	100,000

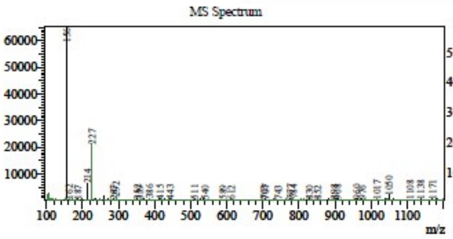

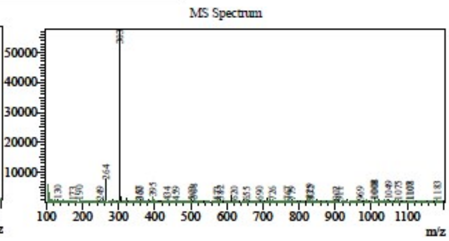

Fig. S48. HPLC-MS data for purified 2-(biphenyl-4-yl)pyridine (14):

Sample Information

Acquired by	: System Administrator
Sample Name	: CsA1067_lio_AT1
Sample ID	:
Tray#	: 1
Vail#	: 36
Injection Volume	: 2 uL
Data Filename	: CsA1067_lio_AT1.lcd
Method Filename	: A_5_100_1ml_13min.lcm
Batch Filename	:
Report Filename	: Default.lsr
Date Acquired	: 2018.12.05. 9:13:59
Data Processed	: 2018.12.05. 10:06:28

Chromatogram
CsA1067_lio_AT1 C:\LabSolutions\Data\Project1\DR\CSA3\CsA1067_lio_AT1.lcd

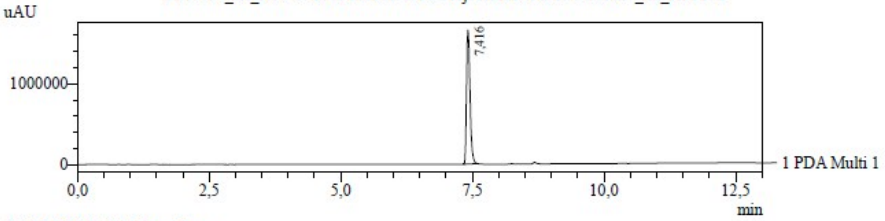

1 PDA Multi 1 / 254nm,4nm

PeakTable C:\LabSolutions\Data\Project1\DR\CSA3\CsA1067_lio_AT1.lcd

PDA Ch1 254nm

Peak#	Ret. Time	Area	Height	Area %	Height %
1	7,416	7678585	1660122	100,000	100,000
Total		7678585	1660122	100,000	100,000

MS Spectrum

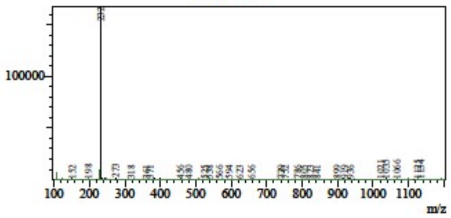

Fig. S49. HPLC-MS data for 2-(biphenyl-4-yl)pyridine (14) by Method A:

Sample Information

Acquired by	: System Administrator
Sample Name	: CSA1070_nyers_lio_AT1
Sample ID	:
Tray#	: 1
Vail#	: 66
Injection Volume	: 2 uL
Data Filename	: CSA1070_nyers_lio_AT1.lcd
Method Filename	: A_5_100_1ml_13min.lcm
Batch Filename	:
Report Filename	: Default.lsr
Date Acquired	: 2018.11.20. 16:18:23
Data Processed	: 2018.12.05. 10:38:48

Chromatogram
CSA1070_nyers_lio_AT1 C:\LabSolutions\Data\Project1\DR\CSA3\CSA1070_nyers_lio_AT1.lcd

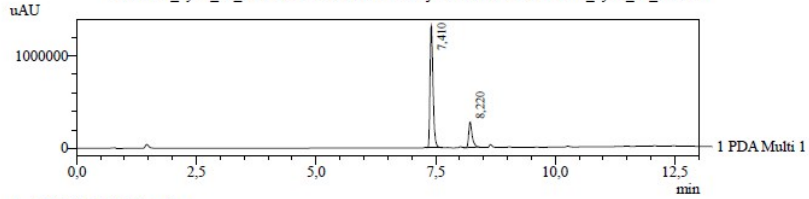

1 PDA Multi 1 / 254nm,4nm

PeakTable C:\LabSolutions\Data\Project1\DR\CSA3\CSA1070_nyers_lio_AT1.lcd

PDA Ch1 254nm

Peak#	Ret. Time	Area	Height	Area %	Height %
1	7,410	5739682	1317429	80,585	82,742
2	8,220	1382859	274791	19,415	17,258
Total		7122541	1592220	100,000	100,000

MS Spectrum

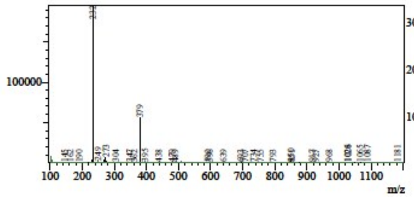

MS Spectrum

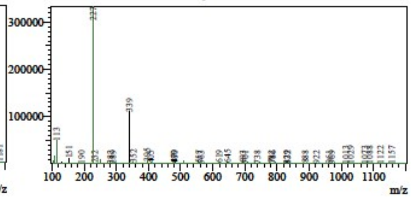

Fig. S50. HPLC-MS data for 2-(biphenyl-4-yl)pyridine (14) by Method B:

Sample Information

Acquired by	: System Administrator
Sample Name	: CsA1068_lio_AT1
Sample ID	:
Tray#	: 1
Vail#	: 6
Injection Volume	: 1 uL
Data Filename	: CsA1068_lio_AT1.lcd
Method Filename	: A_5_100_1ml_13min.lcm
Batch Filename	:
Report Filename	: Default.lsr
Date Acquired	: 2018.11.26. 15:24:41
Data Processed	: 2018.12.05. 10:24:43

Chromatogram
CsA1068_lio_AT1 C:\LabSolutions\Data\Project1\DR\CSA3\CsA1068_lio_AT1.lcd

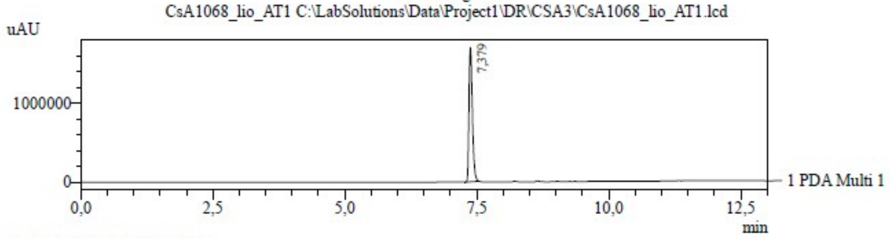

1 PDA Multi 1 / 254nm,4nm

PeakTable C:\LabSolutions\Data\Project1\DR\CSA3\CsA1068_lio_AT1.lcd

PDA Ch1 254nm

Peak#	Ret. Time	Area	Height	Area %	Height %
1	7,379	7534703	1697133	100,000	100,000
Total		7534703	1697133	100,000	100,000

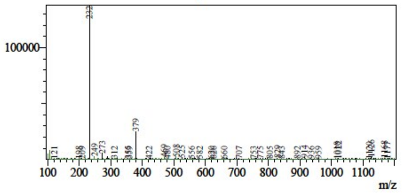

Fig. S51. HPLC-MS data for 2-(biphenyl-4-yl)pyridine (14) by Method B:

Sample Information

Acquired by	: System Administrator
Sample Name	: CSA1071_nyers_lio_AT1
Sample ID	:
Tray#	: 1
Vail#	: 65
Injection Volume	: 2 uL
Data Filename	: CSA1071_nyers_lio_AT1.lcd
Method Filename	: A_5_100_1ml_13min.lcm
Batch Filename	:
Report Filename	: Default.lsr
Date Acquired	: 2018.11.20. 15:59:28
Data Processed	: 2018.12.05. 10:39:41

Chromatogram

CSA1071_nyers_lio_AT1 C:\LabSolutions\Data\Project1\DR\CSA3\CSA1071_nyers_lio_AT1.lcd

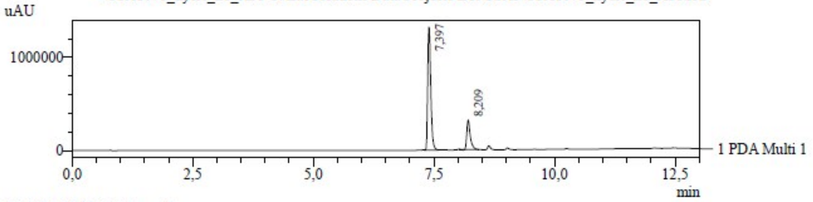

1 PDA Multi 1 / 254nm,4nm

PeakTable C:\LabSolutions\Data\Project1\DR\CSA3\CSA1071_nyers_lio_AT1.lcd

PDA Ch1 254nm

Peak#	Ret. Time	Area	Height	Area %	Height %
1	7,397	5789112	1314397	78,957	80,768
2	8,209	1542828	312983	21,043	19,232
Total		7331940	1627379	100,000	100,000

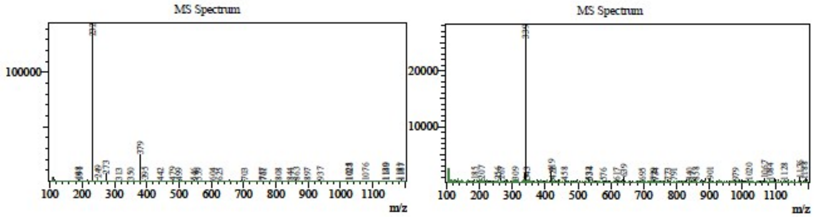

Fig. S52. HPLC-MS data for purified 4-(biphenyl-4-yl)pyridine (15):

Sample Information

Acquired by	: System Administrator
Sample Name	: CsA1063_AT1
Sample ID	:
Tray#	: 1
Vail#	: 71
Injection Volume	: 2 uL
Data Filename	: CsA1063_AT1.lcd
Method Filename	: A_5_100_1ml_13min.lcm
Batch Filename	:
Report Filename	: Default.lsr
Date Acquired	: 2018.12.07. 8:41:51
Data Processed	: 2019.01.30. 11:25:18

Chromatogram
CsA1063_AT1 C:\LabSolutions\Data\Project1\DR\CSA3\CsA1063_AT1.lcd

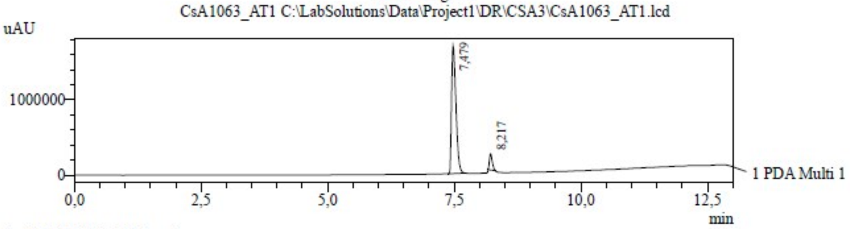

1 PDA Multi 1 / 236nm,4nm

PeakTable C:\LabSolutions\Data\Project1\DR\CSA3\CsA1063_AT1.lcd

PDA Ch1 236nm

Peak#	Ret. Time	Area	Height	Area %	Height %
1	7,479	10367346	1699213	92,406	88,762
2	8,217	851982	215125	7,594	11,238
Total		11219328	1914338	100,000	100,000

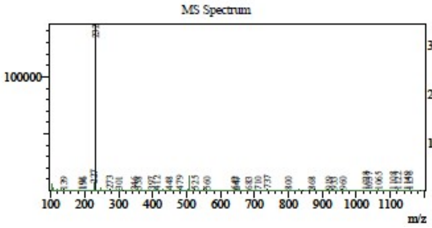

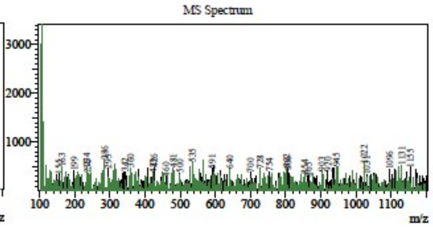

Fig. S53. HPLC-MS data for 4-(biphenyl-4-yl)pyridine (15) by Method A:

Sample Information

Acquired by	: System Administrator
Sample Name	: CSA1066_ny_lio_AT1
Sample ID	:
Tray#	: 1
Vail#	: 91
Injection Volume	: 1 uL
Data Filename	: CSA1066_ny_lio_AT1.lcd
Method Filename	: A_5_100_1ml_13min.lcm
Batch Filename	:
Report Filename	: Default.lsr
Date Acquired	: 2018.11.23. 12:38:41
Data Processed	: 2018.12.05. 10:36:36

Chromatogram
CSA1066_ny_lio_AT1 C:\LabSolutions\Data\Project1\DR\CSA3\CSA1066_ny_lio_AT1.lcd

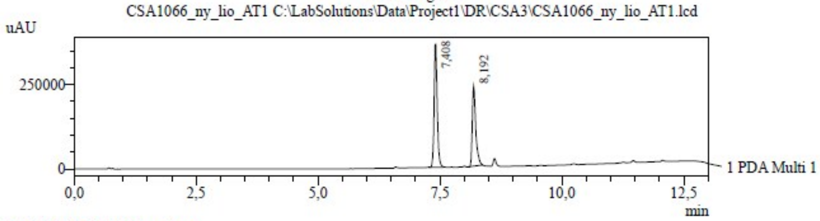

1 PDA Multi 1 / 254nm,4nm

PeakTable C:\LabSolutions\Data\Project1\DR\CSA3\CSA1066_ny_lio_AT1.lcd

PDA Ch1 254nm

Peak#	Ret. Time	Area	Height	Area %	Height %
1	7,408	1587511	364090	57,508	60,633
2	8,192	1173010	236389	42,492	39,367
Total		2760521	600480	100,000	100,000

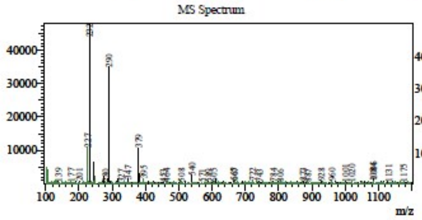

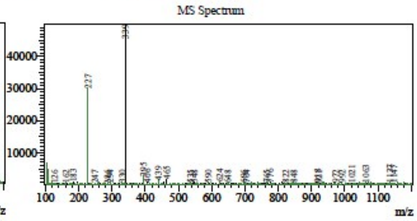

Fig. S54. HPLC-MS data for 4-(biphenyl-4-yl)pyridine (15) by Method B:

Sample Information

Acquired by	: System Administrator
Sample Name	: CsA1063_AT1
Sample ID	:
Tray#	: 1
Vail#	: 71
Injection Volume	: 2 uL
Data Filename	: CsA1063_AT1.lcd
Method Filename	: A_5_100_1ml_13min.lcm
Batch Filename	:
Report Filename	: Default.lsr
Date Acquired	: 2018.12.07. 8:41:51
Data Processed	: 2019.01.30. 11:25:18

Chromatogram

CsA1063_AT1 C:\LabSolutions\Data\Project1\DR\CSA3\CsA1063_AT1.lcd

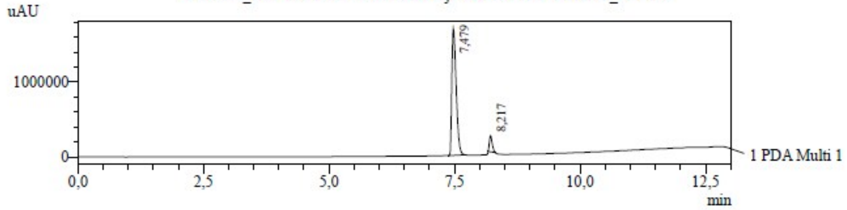

1 PDA Multi 1 / 236nm,4nm

PeakTable C:\LabSolutions\Data\Project1\DR\CSA3\CsA1063_AT1.lcd

PDA Ch1 236nm

Peak#	Ret. Time	Area	Height	Area %	Height %
1	7,479	10367346	1699213	92,406	88,762
2	8,217	851982	215125	7,594	11,238
Total		11219328	1914338	100,000	100,000

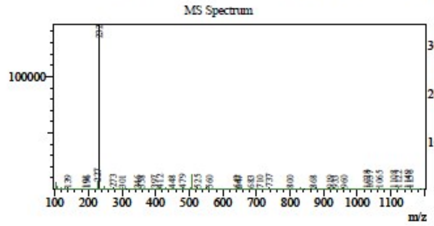

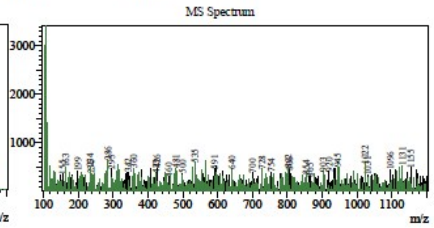

Fig. S55. HPLC-MS data for 4-(biphenyl-4-yl)pyridine (15) by Method B:

Sample Information

Acquired by	: System Administrator
Sample Name	: CsA1065_ny_lio_AT1
Sample ID	:
Tray#	: 1
Vail#	: 73
Injection Volume	: 2 uL
Data Filename	: CsA1065_ny_lio_AT1.lcd
Method Filename	: A_5_100_1ml_13min.lcm
Batch Filename	:
Report Filename	: Default.lsr
Date Acquired	: 2018.12.07. 9:14:04
Data Processed	: 2018.12.07. 9:30:09

Chromatogram
CsA1065_ny_lio_AT1 C:\LabSolutions\Data\Project1\DR\CSA3\CsA1065_ny_lio_AT1.lcd

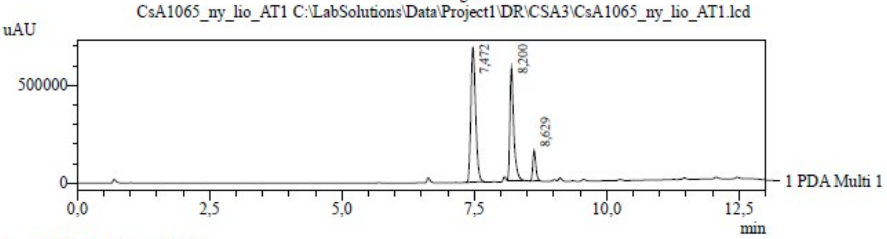

1 PDA Multi 1 / 254nm,4nm

PeakTable C:\LabSolutions\Data\Project1\DR\CSA3\CsA1065_ny_lio_AT1.lcd
PDA Ch1 254nm

Peak#	Ret. Time	Area	Height	Area %	Height %
1	7,472	4293452	686825	55,173	48,485
2	8,200	2877097	574573	36,972	40,561
3	8,629	611209	155179	7,854	10,955
Total		7781759	1416577	100,000	100,000

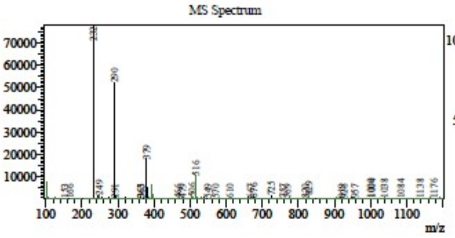

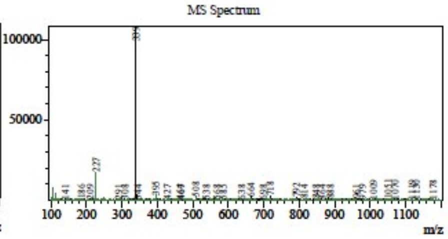

Fig. S56. HPLC-MS data for purified 2-(4-fluorophenyl)pyridine (16):

Sample Information

Acquired by	: System Administrator
Sample Name	: CSA1093_AT1
Sample ID	:
Tray#	: 1
Vail#	: 34
Injection Volume	: 2 uL
Data Filename	: CSA1093_AT1.lcd
Method Filename	: A_5_100_1ml_13min.lcm
Batch Filename	:
Report Filename	: Default.lsr
Date Acquired	: 2019.01.18. 12:15:46
Data Processed	: 2019.01.28. 13:03:31

Chromatogram
CSA1093_AT1 C:\LabSolutions\Data\Project1\DR\CSA5\CSA1093_AT1.lcd

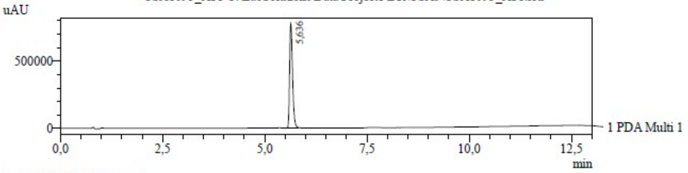

1 PDA Multi 1 / 254nm,4nm

PeakTable C:\LabSolutions\Data\Project1\DR\CSA5\CSA1093_AT1.lcd

PDA Ch1 254nm

Peak#	Ret. Time	Area	Height	Area %	Height %
1	5,636	3783627	770409	100,000	100,000
Total		3783627	770409	100,000	100,000

MS Spectrum

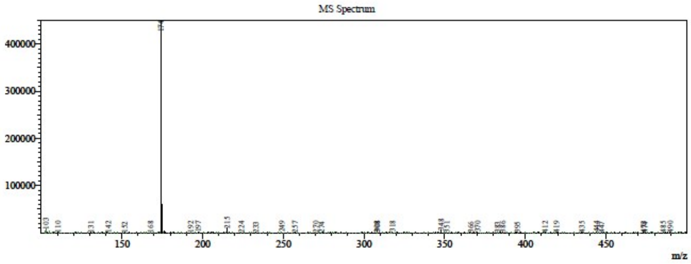

Fig. S57. HPLC-MS data for 2-(4-fluorophenyl)pyridine (16) by Method A:

Sample Information

Acquired by	: System Administrator
Sample Name	: CSA1094_nyers_lio_AT1
Sample ID	:
Tray#	: 1
Vail#	: 1
Injection Volume	: 2 uL
Data Filename	: CSA1094_nyers_lio_AT1.lcd
Method Filename	: A_5_100_1ml_13min.lcm
Batch Filename	:
Report Filename	: Default.lsr
Date Acquired	: 2019.01.29. 13:22:15
Data Processed	: 2019.01.29. 14:29:31

Chromatogram
CSA1094_nyers_lio_AT1 C:\LabSolutions\Data\Project1\DR\CSA5\CSA1094_nyers_lio_AT1.lcd

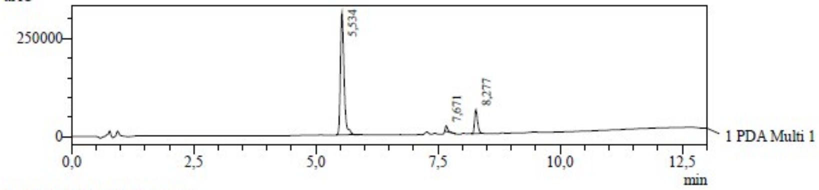

1 PDA Multi 1 / 254nm,4nm

PeakTable C:\LabSolutions\Data\Project1\DR\CSA5\CSA1094_nyers_lio_AT1.lcd

PDA Ch1 254nm

Peak#	Ret. Time	Area	Height	Area %	Height %
1	5,534	1620878	311615	84,081	80,443
2	7,671	50977	14363	2,644	3,708
3	8,277	255897	61396	13,274	15,849
Total		1927752	387374	100,000	100,000

MS Spectrum

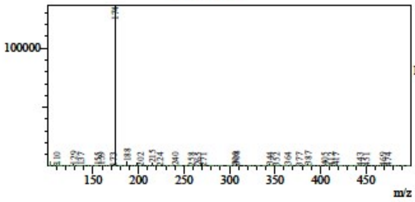

MS Spectrum

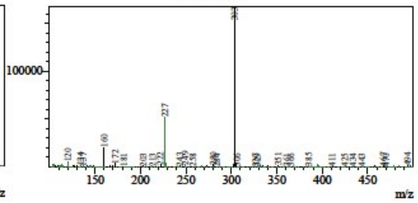

Fig. S58. HPLC-MS data for 2-(4-fluorophenyl)pyridine (16) by Method B:

Sample Information

Acquired by : System Administrator
Sample Name : CSA1095_AT1
Sample ID :
Tray# : 1
Vail# : 101
Injection Volume : 2 uL
Data Filename : CSA1095_AT1.lcd
Method Filename : A_5_100_1ml_13min.lcm
Batch Filename :
Report Filename : Default.lsr
Date Acquired : 2019.01.29. 12:38:22
Data Processed : 2019.01.29. 14:32:45

Chromatogram
CSA1095_AT1 C:\LabSolutions\Data\Project1\DR\CSA5\CSA1095_AT1.lcd

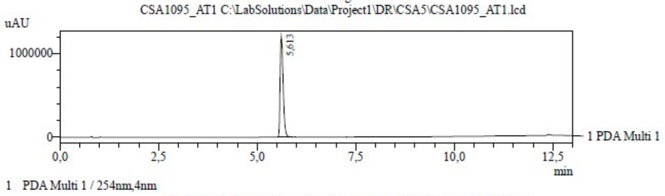

1 PDA Multi 1 / 254nm,4nm

PeakTable C:\LabSolutions\Data\Project1\DR\CSA5\CSA1095_AT1.lcd

PDA Ch1 254nm

Peak#	Ret. Time	Area	Height	Area %	Height %
1	5,613	6427984	1198238	100,000	100,000
Total		6427984	1198238	100,000	100,000

MS Spectrum

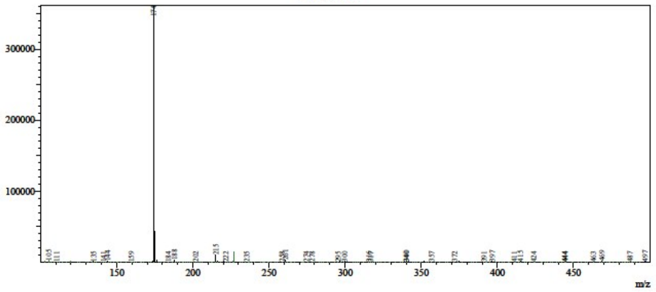

Fig. S59. HPLC-MS data for 2-(4-fluorophenyl)pyridine (16) by Method B:

Sample Information

Acquired by : System Administrator
Sample Name : CSA1096_nyers_lio_AT1
Sample ID :
Tray# : 1
Vail# : 98
Injection Volume : 2 uL
Data Filename : CSA1096_nyers_lio_AT1.lcd
Method Filename : A_5_100_1ml_13min.lcm
Batch Filename :
Report Filename : Default.lsr
Date Acquired : 2019.01.29. 11:02:23
Data Processed : 2019.01.29. 12:08:38

Chromatogram
CSA1096_nyers_lio_AT1 C:\LabSolutions\Data\Project1\DR\CSA5\CSA1096_nyers_lio_AT1.lcd

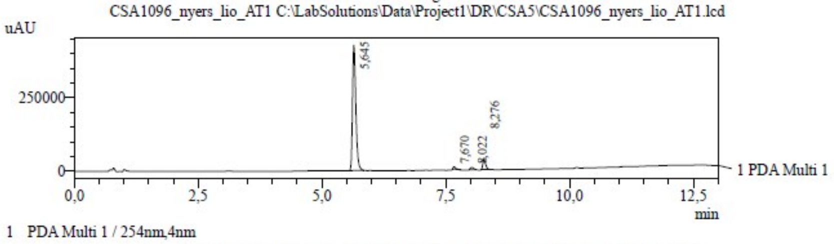

1 PDA Multi 1 / 254nm,4nm

PeakTable C:\LabSolutions\Data\Project1\DR\CSA5\CSA1096_nyers_lio_AT1.lcd

PDA Ch1 254nm

Peak#	Ret. Time	Area	Height	Area %	Height %
1	5,645	1967960	426229	91,231	89,856
2	7,670	26262	7726	1,217	1,629
3	8,022	38244	7611	1,773	1,605
4	8,276	124658	32783	5,779	6,911
Total		2157125	474349	100,000	100,000

MS Spectrum

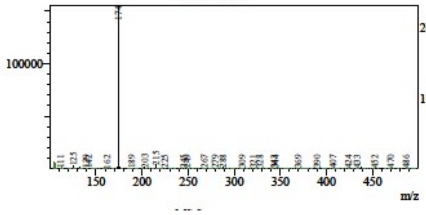

MS Spectrum

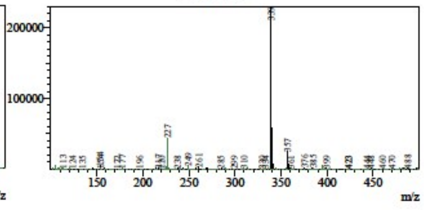

Fig. S60. HPLC-MS data for purified 4-(4-fluorophenyl)pyridine (17):

Sample Information

Acquired by	: System Administrator
Sample Name	: CSA1086_ny_lio_AT1
Sample ID	:
Tray#	: 1
Vail#	: 17
Injection Volume	: 2 uL
Data Filename	: CSA1086_ny_lio_AT3.lcd
Method Filename	: A_5_100_1ml_13min.lcm
Batch Filename	:
Report Filename	: Default.lsr
Date Acquired	: 2019.01.16. 9:22:24
Data Processed	: 2019.01.16. 15:07:54

Chromatogram

CSA1086_ny_lio_AT1 C:\LabSolutions\Data\Project1\DR\CSA4\CSA1086_ny_lio_AT3.lcd

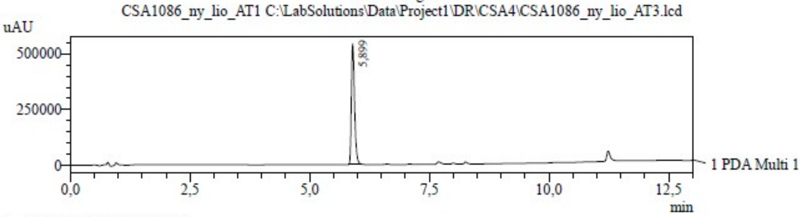

1 PDA Multi 1 / 254nm,4nm

PeakTable C:\LabSolutions\Data\Project1\DR\CSA4\CSA1086_ny_lio_AT3.lcd

PDA Ch1 254nm

Peak#	Ret. Time	Area	Height	Area %	Height %
1	5,899	2485864	539817	100,000	100,000
Total		2485864	539817	100,000	100,000

MS Spectrum

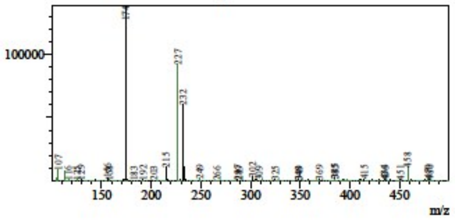

Fig. S61. HPLC-MS data for 4-(4-fluorophenyl)pyridine (17) by Method A:

Sample Information

Acquired by	: System Administrator
Sample Name	: CSA1087_nyers_lio_AT1
Sample ID	:
Tray#	: 1
Vail#	: 93
Injection Volume	: 2 uL
Data Filename	: CSA1087_nyers_lio_AT1.lcd
Method Filename	: A_5_100_1ml_13min.lcm
Batch Filename	:
Report Filename	: Default.lsr
Date Acquired	: 2019.01.29. 9:43:59
Data Processed	: 2019.01.29. 12:06:02

Chromatogram
CSA1087_nyers_lio_AT1 C:\LabSolutions\Data\Project1\DR\CSA5\CSA1087_nyers_lio_AT1.lcd

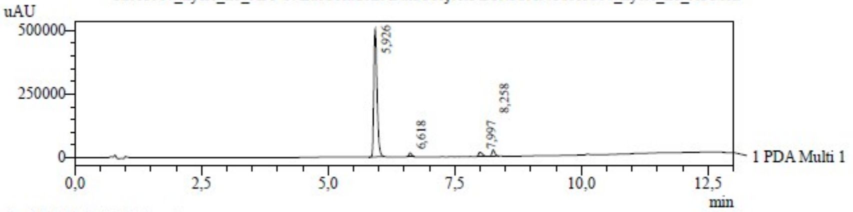

1 PDA Multi 1 / 254nm,4nm

PeakTable C:\LabSolutions\Data\Project1\DR\CSA5\CSA1087_nyers_lio_AT1.lcd

PDA Ch1 254nm

Peak#	Ret. Time	Area	Height	Area %	Height %
1	5,926	2226704	503066	90,542	90,172
2	6,618	46606	13297	1,895	2,383
3	7,997	84969	16450	3,455	2,949
4	8,258	101032	25083	4,108	4,496
Total		2459311	557895	100,000	100,000

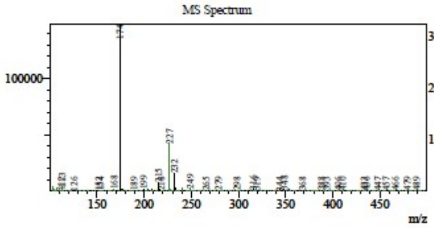

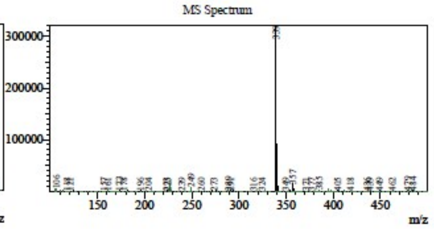

Fig. S62. HPLC-MS data for 4-(4-fluorophenyl)pyridine (17) by Method B:

Sample Information

Acquired by	: System Administrator
Sample Name	: CSA1085_ny_lio_AT1
Sample ID	:
Tray#	: 1
Vail#	: 9
Injection Volume	: 3 uL
Data Filename	: CSA1085_ny_lio_AT1.lcd
Method Filename	: A_5_100_1ml_13min.lcm
Batch Filename	: 20180725.lcb
Report Filename	: DEFAULT.lsr
Date Acquired	: 2019.01.15. 11:44:49
Data Processed	: 2019.01.16. 15:05:48

Chromatogram
CSA1085_ny_lio_AT1 C:\LabSolutions\Data\Project1\DR\CSA4\CSA1085_ny_lio_AT1.lcd

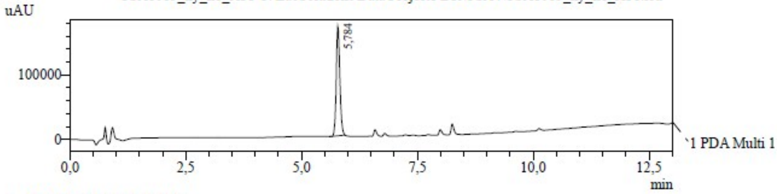

1 PDA Multi 1 / 254nm,4nm

PeakTable C:\LabSolutions\Data\Project1\DR\CSA4\CSA1085_ny_lio_AT1.lcd

PDA Ch1 254nm

Peak#	Ret. Time	Area	Height	Area %	Height %
1	5,784	920474	169950	100,000	100,000
Total		920474	169950	100,000	100,000

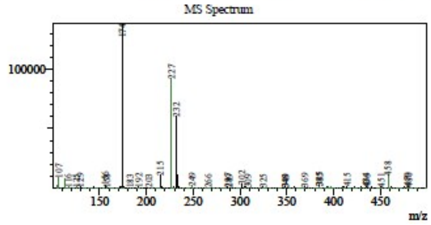

Fig. S63. HPLC-MS data for 4-(4-fluorophenyl)pyridine (17) by Method B:

Sample Information

Acquired by	: System Administrator
Sample Name	: CSA1091_nyers_lio_AT1
Sample ID	:
Tray#	: 1
Vail#	: 92
Injection Volume	: 2 uL
Data Filename	: CSA1091_nyers_lio_AT1.lcd
Method Filename	: A_5_100_1ml_13min.lcm
Batch Filename	:
Report Filename	: Default.lsr
Date Acquired	: 2019.01.29. 9:27:03
Data Processed	: 2019.01.29. 12:04:18

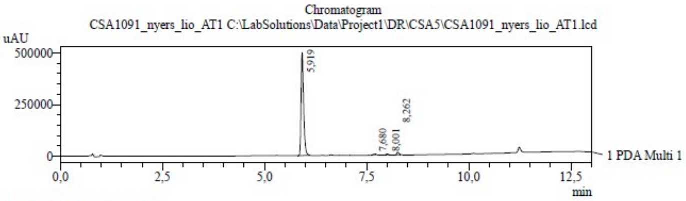

1 PDA Multi 1 / 254nm,4nm

PeakTable C:\LabSolutions\Data\Project1\DR\CSA5\CSA1091_nyers_lio_AT1.lcd

PDA Ch1 254nm

Peak#	Ret. Time	Area	Height	Area %	Height %
1	5,919	2177887	498944	97,197	96,523
2	7,680	5641	2063	0,252	0,399
3	8,001	11067	3877	0,494	0,750
4	8,262	46105	12034	2,058	2,328
Total		2240700	516918	100,000	100,000

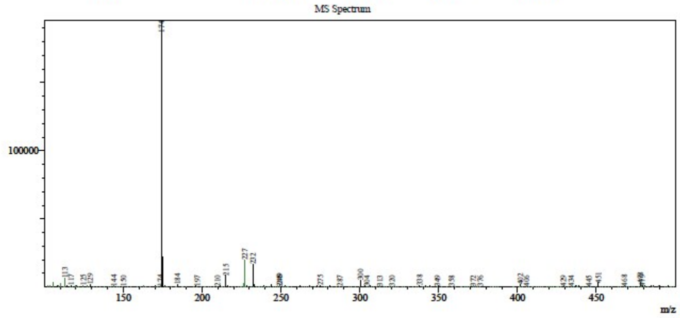

Fig. S64. HPLC-MS data for purified 6-phenylpyridazin-3(2*H*)-one (18a):

Sample Information

Acquired by	: System Administrator
Sample Name	: CSA1088_F_AT1
Sample ID	:
Tray#	: 1
Vail#	: 22
Injection Volume	: 2 uL
Data Filename	: CSA1088_F_AT1.lcd
Method Filename	: A_5_100_1ml_13min.lcm
Batch Filename	:
Report Filename	: Default.lsr
Date Acquired	: 2019.01.16. 14:41:18
Data Processed	: 2019.01.16. 15:48:28

Chromatogram
CSA1088_F_AT1 C:\LabSolutions\Data\Project1\DR\CSA4\CSA1088_F_AT1.lcd

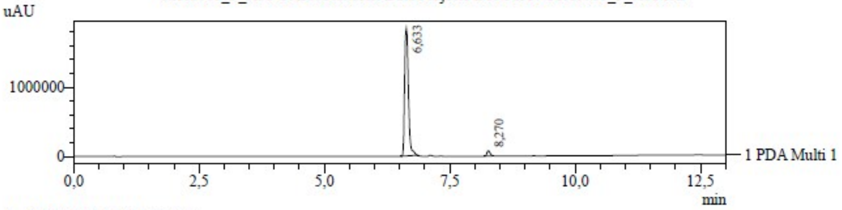

1 PDA Multi 1 / 254nm,4nm

PeakTable C:\LabSolutions\Data\Project1\DR\CSA4\CSA1088_F_AT1.lcd

PDA Ch1 254nm

Peak#	Ret. Time	Area	Height	Area %	Height %
1	6,633	9669890	1844191	96,734	95,930
2	8,270	326448	78241	3,266	4,070
Total		9996338	1922433	100,000	100,000

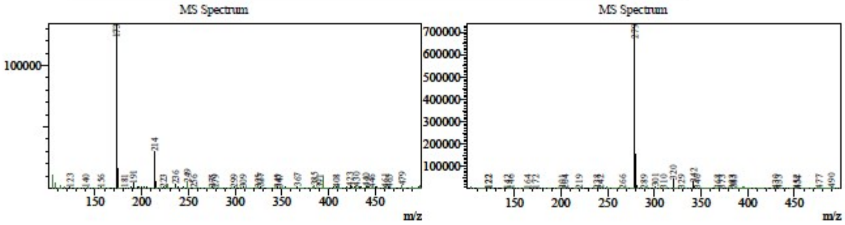

Fig. S65. HPLC-MS data for 6-phenylpyridazin-3(2*H*)-one (18a) by Method A

Sample Information

Acquired by	: System Administrator
Sample Name	: CsA_1001_4_AT1
Sample ID	:
Tray#	: 1
Vail#	: 22
Injection Volume	: 2 uL
Data Filename	: CsA_1001_4_AT1.lcd
Method Filename	: A_5_100_1ml_13min.lcm
Batch Filename	: Batch03_re.lcb
Report Filename	: DEFAULT.lsr
Date Acquired	: 2017.12.15. 11:00:17
Data Processed	: 2017.12.15. 13:49:17

Chromatogram
CsA_1001_4_AT1 C:\LabSolutions\Data\Project1\DR\CsA_1001_4_AT1.lcd

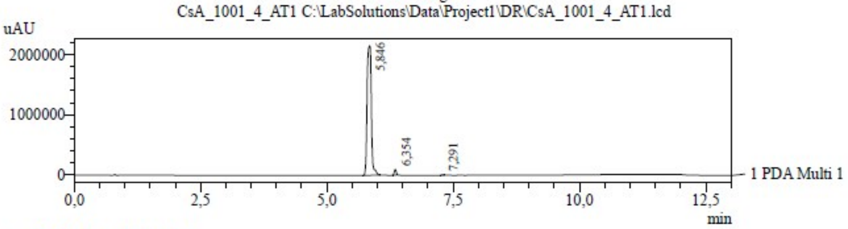

1 PDA Multi 1 / 254nm,4nm

PeakTable C:\LabSolutions\Data\Project1\DR\CsA_1001_4_AT1.lcd

PDA Ch1 254nm

Peak#	Ret. Time	Area	Height	Area %	Height %
1	5,846	12436930	2144643	97,917	95,641
2	6,354	246725	90786	1,942	4,049
3	7,291	17854	6963	0,141	0,311
Total		12701510	2242393	100,000	100,000

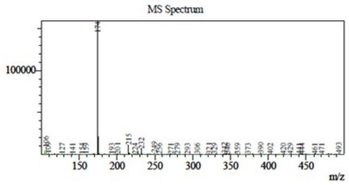

Fig. S66. HPLC-MS data for 6-phenylpyridazin-3(2*H*)-one (18a) and 2-(2-hydroxypropyl)-6-phenylpyridazin-3(2*H*)-one (18b) by Method A:

Sample Information

Acquired by	: System Administrator
Sample Name	: CSA1088_ny_lio_AT1
Sample ID	:
Tray#	: 1
Vail#	: 2
Injection Volume	: 3 uL
Data Filename	: CSA1088_ny_lio_AT1.lcd
Method Filename	: A_5_100_1ml_13min.lcm
Batch Filename	: 20180725.lcb
Report Filename	: DEFAULT.lsr
Date Acquired	: 2019.01.15. 9:25:45
Data Processed	: 2019.01.16. 15:49:31

Chromatogram
CSA1088_ny_lio_AT1 C:\LabSolutions\Data\Project1\DR\CSA4\CSA1088_ny_lio_AT1.lcd

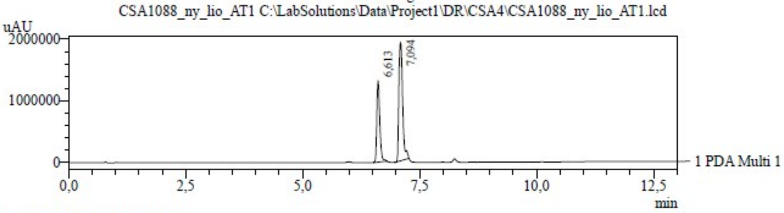

1 PDA Multi 1 / 254nm,4nm

PeakTable C:\LabSolutions\Data\Project1\DR\CSA4\CSA1088_ny_lio_AT1.lcd
PDA Ch1 254nm

Peak#	Ret. Time	Area	Height	Area %	Height %
1	6,613	5791441	1324028	34,520	40,981
2	7,094	10985694	1906836	65,480	59,019
Total		16777135	3230865	100,000	100,000

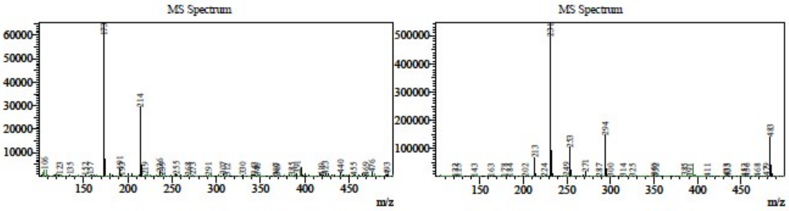

Fig. S67. HPLC-MS data for purified 2-(2-hydroxypropyl)-6-phenylpyridazin-3(2H)-one (18b):

Sample Information

Acquired by	: System Administrator
Sample Name	: CSA1088A_AT1
Sample ID	:
Tray#	: 1
Vail#	: 14
Injection Volume	: 3 uL
Data Filename	: CSA1088A_AT1.lcd
Method Filename	: A_5_100_1ml_13min.lcm
Batch Filename	: 20180725.lcb
Report Filename	: DEFAULT.lsr
Date Acquired	: 2019.01.15. 12:53:09
Data Processed	: 2019.01.16. 15:50:15

Chromatogram

CSA1088A_AT1 C:\LabSolutions\Data\Project1\DR\CSA4\CSA1088A_AT1.lcd

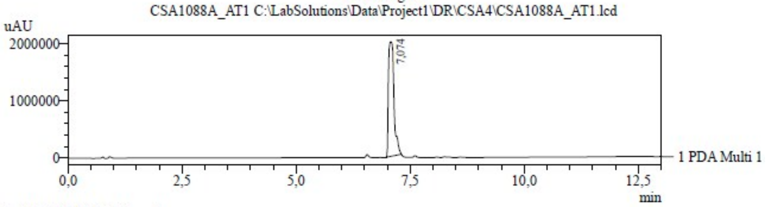

1 PDA Multi 1 / 254nm,4nm

PeakTable C:\LabSolutions\Data\Project1\DR\CSA4\CSA1088A_AT1.lcd

PDA Ch1 254nm

Peak#	Ret. Time	Area	Height	Area %	Height %
1	7,074	16853683	2003820	100,000	100,000
Total		16853683	2003820	100,000	100,000

MS Spectrum

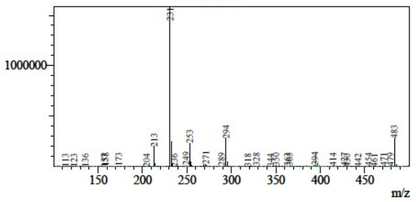

Fig. S68. HPLC-MS data for 2-(2-hydroxypropyl)-6-phenylpyridazin-3(2*H*)-one (18b) by Method B:

Sample Information

Acquired by	: System Administrator
Sample Name	: CsA_1001_6_AT1
Sample ID	:
Tray#	: 1
Vail#	: 23
Injection Volume	: 2 uL
Data Filename	: CsA_1001_6_AT1.lcd
Method Filename	: A_5_100_1ml_13min.lcm
Batch Filename	: Batch03_re.lcb
Report Filename	: DEFAULT.lsr
Date Acquired	: 2017.12.15. 11:14:01
Data Processed	: 2017.12.15. 13:52:33

Chromatogram

CsA_1001_6_AT1 C:\LabSolutions\Data\Project1\DR\CsA_1001_6_AT1.lcd

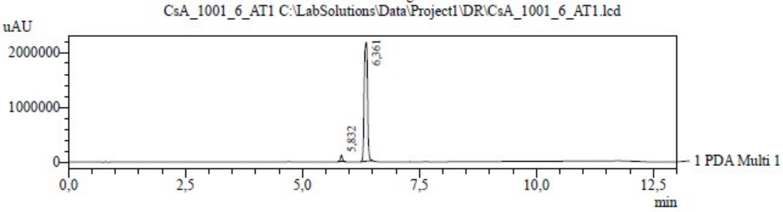

1 PDA Multi 1 / 254nm,4nm

PeakTable C:\LabSolutions\Data\Project1\DR\CsA_1001_6_AT1.lcd

PDA Ch1 254nm

Peak#	Ret. Time	Area	Height	Area %	Height %
1	5,832	378209	122384	3,407	5,355
2	6,361	10722197	2162950	96,593	94,645
Total		11100406	2285335	100,000	100,000

MS Spectrum

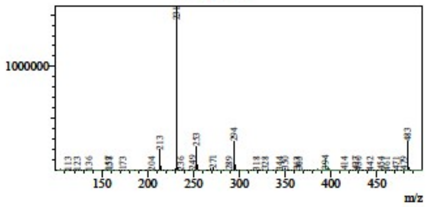

Fig. S69. HPLC-MS data for 6-phenylpyridazin-3(2*H*)-one (18a) and 2-(2-hydroxypropyl)-6-phenylpyridazin-3(2*H*)-one (18b) by Method B:

Sample Information

Acquired by	: System Administrator
Date Acquired	: 2018.06.26. 11:17:36
Sample Name	: CSA1045_lio_AT1
Vial#	: 68
Injection Volume	: 2
Data File	: CSA1045_lio_AT1.lcd
Method File	: A_5_100_1ml_13min.lcm
Tuning File	: Tuning_20170803.lct

Chromatogram

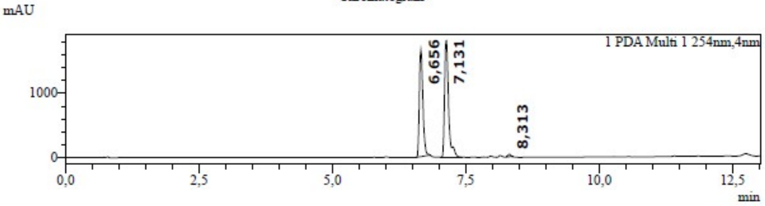

Peak Table

PDA Ch1 254nm

Peak#	Ret. Time	Area	Height	Area%
1	6.656	7654285	1660246	44.177
2	7.131	9537644	1812695	55.047
3	8.313	134370	38623	0.776
Total		17326299	3511564	100.000

MS Spectrum

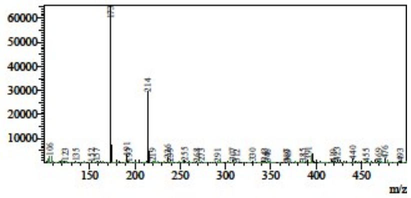

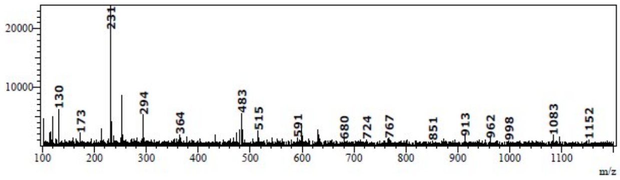

Fig. S70. HPLC-MS data for purified 6-(biphenyl-4-yl)pyridazin-3(2*H*)-one (19a):

Sample Information

Acquired by	: System Administrator
Sample Name	: CSA1058F_AT1
Sample ID	:
Tray#	: 1
Vail#	: 41
Injection Volume	: 2 uL
Data Filename	: CSA1058F_AT1.lcd
Method Filename	: A_5_100_1ml_13min.lcm
Batch Filename	:
Report Filename	: Default.lsr
Date Acquired	: 2018.11.28. 16:49:55
Data Processed	: 2018.12.05. 10:08:26

Chromatogram

CSA1058F_AT1 C:\LabSolutions\Data\Project1\DR\CSA3\CSA1058F_AT1.lcd

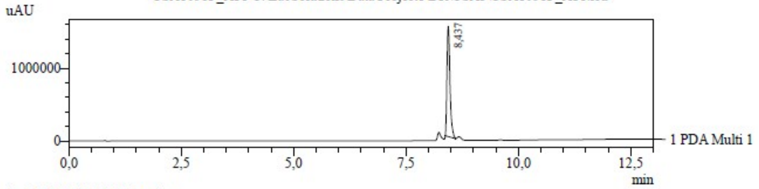

1 PDA Multi 1 / 254nm,4nm

PeakTable C:\LabSolutions\Data\Project1\DR\CSA3\CSA1058F_AT1.lcd

PDA Ch1 254nm

Peak#	Ret. Time	Area	Height	Area %	Height %
1	8,437	7107770	1516824	100,000	100,000
Total		7107770	1516824	100,000	100,000

MS Spectrum

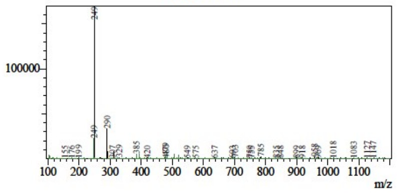

Fig. S71. HPLC-MS data for 6-(biphenyl-4-yl)pyridazin-3(2*H*)-one (19a) by Method A:

Sample Information

Acquired by	: System Administrator
Date Acquired	: 2018.09.04. 10:02:50
Sample Name	: CsA1057_F_AT1
Vial#	: 6
Injection Volume	: 2
Data File	: CsA1057_F_AT1.lcd
Method File	: A_5_100_1ml_13min.lcm
Tuning File	: Tuning_20170803.lct

Chromatogram

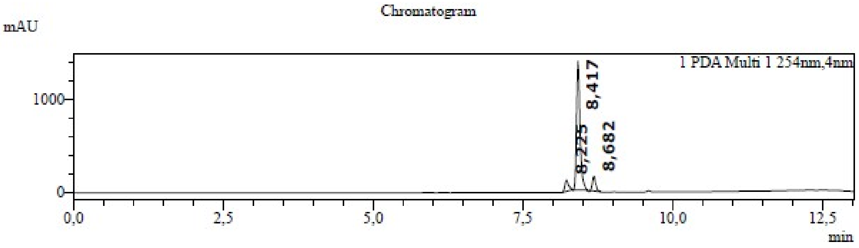

Peak Table

PDA Ch1 254nm

Peak#	Ret. Time	Area	Height	Area%
1	8.225	539733	121233	7.652
2	8.417	5941707	1388606	84.242
3	8.682	571690	154511	8.105
Total		7053131	1664350	100.000

MS Spectrum

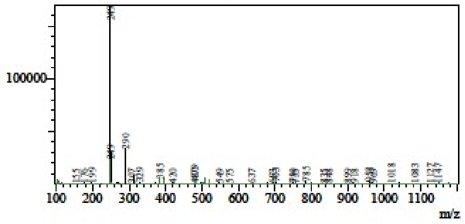

Fig. S72. HPLC-MS data for 6-(biphenyl-4-yl)pyridazin-3(2*H*)-one (19a) and 6-(biphenyl-4-yl)-2-(2-hydroxypropyl)-pyridazin-3(2*H*)-one (19b) by Method A:

Sample Information

Acquired by	: System Administrator
Sample Name	: CSA1059_ny_lio2_AT1
Sample ID	:
Tray#	: 1
Vail#	: 69
Injection Volume	: 2 uL
Data Filename	: CSA1059_ny_lio2_AT1.lcd
Method Filename	: A_5_100_1ml_13min.lcm
Batch Filename	:
Report Filename	: Default.lsr
Date Acquired	: 2018.11.20. 17:05:01
Data Processed	: 2018.12.05. 10:35:33

Chromatogram
CSA1059_ny_lio2_AT1 C:\LabSolutions\Data\Project1\DR\CSA3\CSA1059_ny_lio2_AT1.lcd

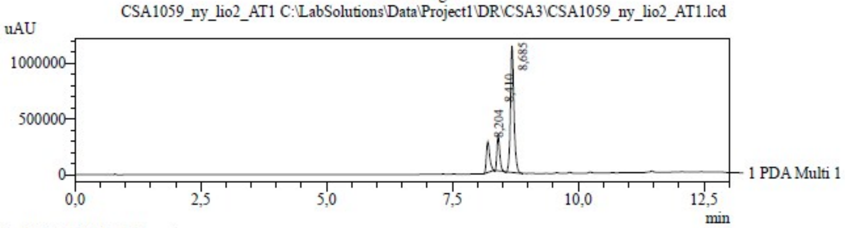

1 PDA Multi 1 / 254nm,4nm

PeakTable C:\LabSolutions\Data\Project1\DR\CSA3\CSA1059_ny_lio2_AT1.lcd
PDA Ch1 254nm

Peak#	Ret. Time	Area	Height	Area %	Height %
1	8,204	1266932	276016	15,944	15,966
2	8,410	1240390	315652	15,610	18,259
3	8,685	5438718	1137112	68,446	65,775
Total		7946040	1728780	100,000	100,000

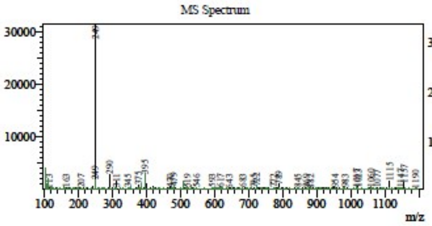

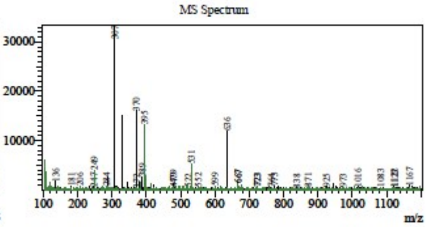

Fig. S73. HPLC-MS data for purified 6-(biphenyl-4-yl)-2-(2-hydroxypropyl)pyridazin-3(2H)-one (19b):

Sample Information

Acquired by	: System Administrator
Sample Name	: CSA1058A_AT1
Sample ID	:
Tray#	: 1
Vail#	: 92
Injection Volume	: 1 uL
Data Filename	: CSA1058A_AT1.lcd
Method Filename	: A_5_100_1ml_13min.lcm
Batch Filename	:
Report Filename	: Default.lsr
Date Acquired	: 2018.11.23. 14:13:20
Data Processed	: 2018.12.05. 10:33:34

Chromatogram

CSA1058A_AT1 C:\LabSolutions\Data\Project1\DR\CSA3\CSA1058A_AT1.lcd

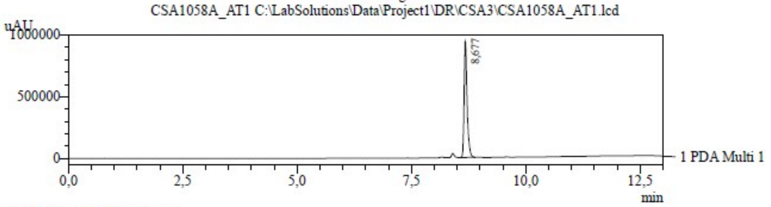

1 PDA Multi 1 / 254nm,4nm

PeakTable C:\LabSolutions\Data\Project1\DR\CSA3\CSA1058A_AT1.lcd

PDA Ch1 254nm

Peak#	Ret. Time	Area	Height	Area %	Height %
1	8,677	4294413	938108	100,000	100,000
Total		4294413	938108	100,000	100,000

MS Spectrum

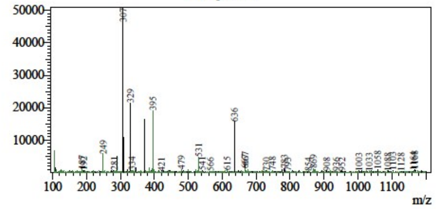

Fig. S74. HPLC-MS data for 6-(biphenyl-4-yl)-2-(2-hydroxypropyl)pyridazin-3(2*H*)-one (19b) by Method B:

Sample Information

Acquired by	: System Administrator
Sample Name	: CSA1057A_lio_AT1
Sample ID	:
Tray#	: 1
Vail#	: 30
Injection Volume	: 2 uL
Data Filename	: CSA1057A_lio_AT1.lcd
Method Filename	: A_5_100_1ml_13min.lcm
Batch Filename	:
Report Filename	: Default.lsr
Date Acquired	: 2018.12.06. 10:32:36
Data Processed	: 2018.12.06. 10:59:12

Chromatogram
CSA1057A_lio_AT1 C:\LabSolutions\Data\Project1\DR\CSA3\CSA1057A_lio_AT1.lcd

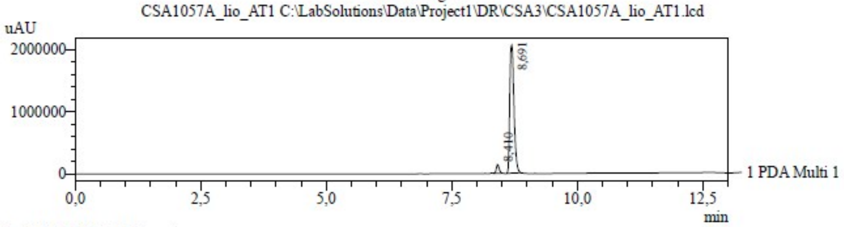

1 PDA Multi 1 / 254nm,4nm

PeakTable C:\LabSolutions\Data\Project1\DR\CSA3\CSA1057A_lio_AT1.lcd

PDA Ch1 254nm

Peak#	Ret. Time	Area	Height	Area %	Height %
1	8,410	552174	140933	4,370	6,428
2	8,691	12084704	2051404	95,630	93,572
Total		12636878	2192337	100,000	100,000

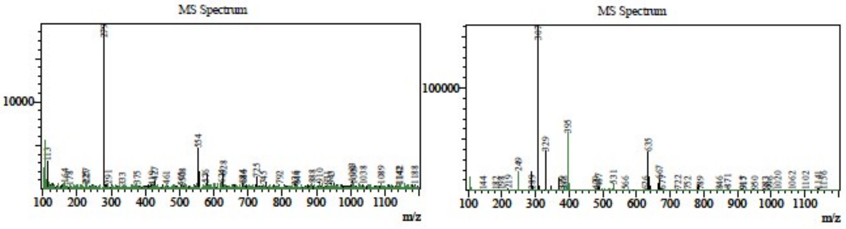

Fig. S75. HPLC-MS data for 6-(biphenyl-4-yl)pyridazin-3(2*H*)-one (19a) and 6-(biphenyl-4-yl)-2-(2-hydroxypropyl)-pyridazin-3(2*H*)-one (19b) by Method B:

Sample Information

Acquired by	: System Administrator
Sample Name	: CsA1060_ny_lio_AT1
Sample ID	:
Tray#	: 1
Vail#	: 20
Injection Volume	: 1 uL
Data Filename	: CsA1060_ny_lio_AT1.lcd
Method Filename	: A_5_100_1ml_13min.lcm
Batch Filename	:
Report Filename	: Default.lsr
Date Acquired	: 2018.11.28. 9:26:11
Data Processed	: 2018.12.05. 10:12:38

Chromatogram
CsA1060_ny_lio_AT1 C:\LabSolutions\Data\Project1\DR\CSA3\CsA1060_ny_lio_AT1.lcd

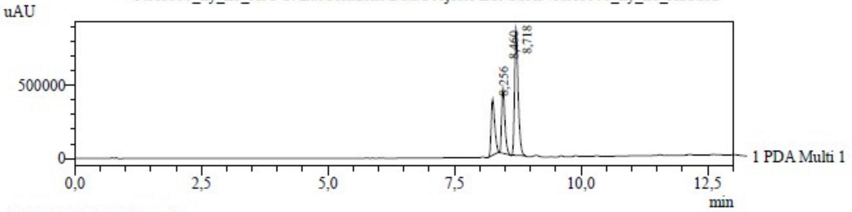

1 PDA Multi 1 / 254nm,4nm

PeakTable C:\LabSolutions\Data\Project1\DR\CSA3\CsA1060_ny_lio_AT1.lcd

PDA Ch1 254nm

Peak#	Ret. Time	Area	Height	Area %	Height %
1	8,256	1889375	382015	23,486	22,597
2	8,460	1957650	445110	24,335	26,330
3	8,718	4197499	863399	52,178	51,073
Total		8044524	1690525	100,000	100,000

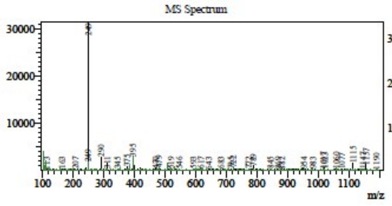

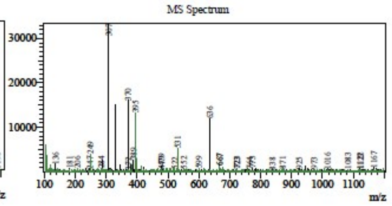

Fig. *S*76. HPLC-MS data for purified 6-(4-fluorophenyl)pyridazin-3(2*H*)-one (20a):

Sample Information

Acquired by	: System Administrator
Sample Name	: CSA1076_F_AT1
Sample ID	:
Tray#	: 1
Vail#	: 21
Injection Volume	: 2 uL
Data Filename	: CSA1076_F_AT1.lcd
Method Filename	: A_5_100_1ml_13min.lcm
Batch Filename	:
Report Filename	: Default.lsr
Date Acquired	: 2019.01.16. 14:23:51
Data Processed	: 2019.01.16. 14:41:42

Chromatogram

CSA1076_F_AT1 C:\LabSolutions\Data\Project1\DR\CSA4\CSA1076_F_AT1.lcd

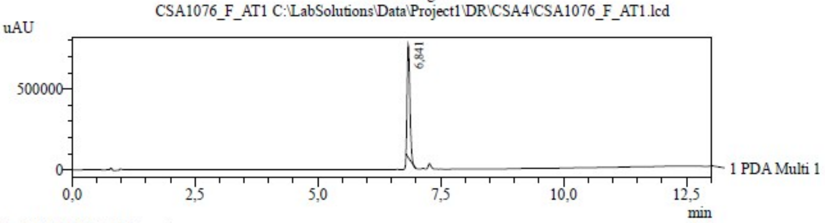

1 PDA Multi 1 / 254nm,4nm

PeakTable C:\LabSolutions\Data\Project1\DR\CSA4\CSA1076_F_AT1.lcd

PDA Ch1 254nm

Peak#	Ret. Time	Area	Height	Area %	Height %
1	6,841	2651299	697997	100,000	100,000
Total		2651299	697997	100,000	100,000

MS Spectrum

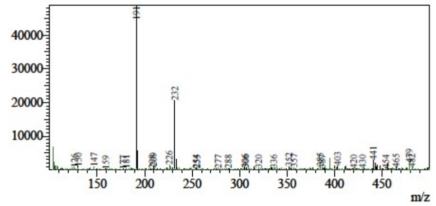

Fig. *S*77. HPLC-MS data for purified 6-(4-fluorophenyl)-2-(2-hydroxypropyl)pyridazin-3(2*H*)-one (20b):

Sample Information

Acquired by	: System Administrator
Sample Name	: CSA1076A_AT1
Sample ID	:
Tray#	: 1
Vail#	: 20
Injection Volume	: 2 uL
Data Filename	: CSA1076A_AT1.lcd
Method Filename	: A_5_100_1ml_13min.lcm
Batch Filename	: 20180725.lcb
Report Filename	: DEFAULT.lsr
Date Acquired	: 2019.01.16. 14:08:54
Data Processed	: 2019.01.16. 14:51:47

Chromatogram
CSA1076A_AT1 C:\LabSolutions\Data\Project1\DR\CSA4\CSA1076A_AT1.lcd

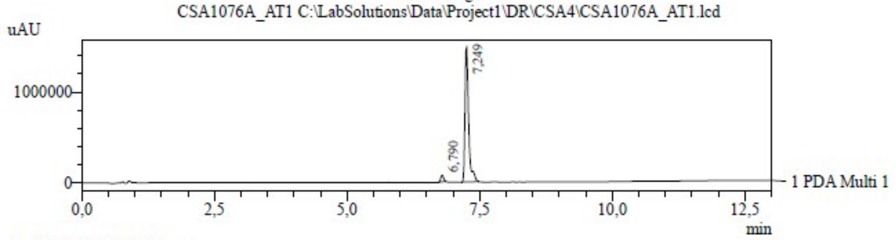

1 PDA Multi 1 / 254nm,4nm

PeakTable C:\LabSolutions\Data\Project1\DR\CSA4\CSA1076A_AT1.lcd

PDA Ch1 254nm

Peak#	Ret. Time	Area	Height	Area %	Height %
1	6,790	281411	73764	3,806	4,752
2	7,249	7112876	1478393	96,194	95,248
Total		7394287	1552156	100,000	100,000

MS Spectrum

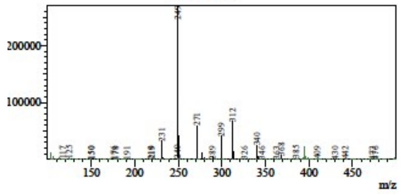

Fig. S78. HPLC-MS data for 6-(4-fluorophenyl)pyridazin-3(2*H*)-one (20a) and 6-(4-fluorophenyl)-2-(2-hydroxypro-pyl)pyridazin-3(2*H*)-one (20b) by Method A:

Sample Information

Acquired by	: System Administrator
Sample Name	: CSA1082_ny_lio_AT1
Sample ID	:
Tray#	: 1
Vail#	: 5
Injection Volume	: 3 uL
Data Filename	: CSA1082_ny_lio_AT1.lcd
Method Filename	: A_5_100_1ml_13min.lcm
Batch Filename	: 20180725.lcb
Report Filename	: DEFAULT.lsr
Date Acquired	: 2019.01.15. 10:06:46
Data Processed	: 2019.01.16. 15:03:41

Chromatogram
CSA1082_ny_lio_AT1 C:\LabSolutions\Data\Project1\DR\CSA4\CSA1082_ny_lio_AT1.lcd

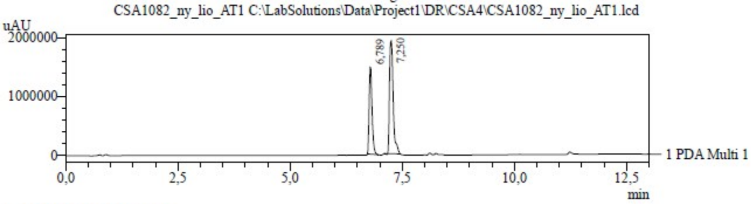

1 PDA Multi 1 / 254nm,4nm

PeakTable C:\LabSolutions\Data\Project1\DR\CSA4\CSA1082_ny_lio_AT1.lcd
PDA Ch1 254nm

Peak#	Ret. Time	Area	Height	Area %	Height %
1	6,789	6677942	1470701	37,701	43,513
2	7,250	11034942	1909177	62,299	56,487
Total		17712884	3379877	100,000	100,000

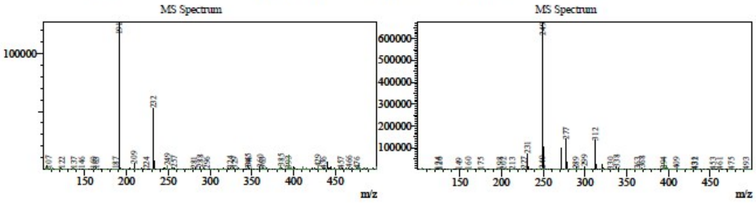

Fig. S79. HPLC-MS data for 6-(4-fluorophenyl)-2-(2-hydroxypropyl)pyridazin-3(2*H*)-one (20b) by Method B:

Sample Information

Acquired by	: System Administrator
Sample Name	: CSA1084_nyers_lio_AT1
Sample ID	:
Tray#	: 1
Vail#	: 94
Injection Volume	: 2 uL
Data Filename	: CSA1084_nyers_lio_AT1.lcd
Method Filename	: A_5_100_1ml_13min.lcm
Batch Filename	:
Report Filename	: Default.lsr
Date Acquired	: 2019.01.29. 9:59:21
Data Processed	: 2019.01.29. 12:07:01

Chromatogram
CSA1084_nyers_lio_AT1 C:\LabSolutions\Data\Project1\DR\CSA5\CSA1084_nyers_lio_AT1.lcd

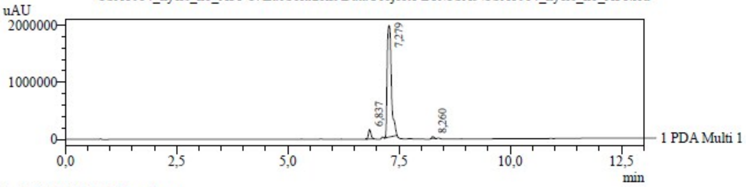

1 PDA Multi 1 / 254nm,4nm

PeakTable C:\LabSolutions\Data\Project1\DR\CSA5\CSA1084_nyers_lio_AT1.lcd
PDA Ch1 254nm

Peak#	Ret. Time	Area	Height	Area %	Height %
1	6,837	662170	167057	4,835	7,770
2	7,279	12934411	1950243	94,444	90,713
3	8,260	98808	32604	0,721	1,517
Total		13695389	2149904	100,000	100,000

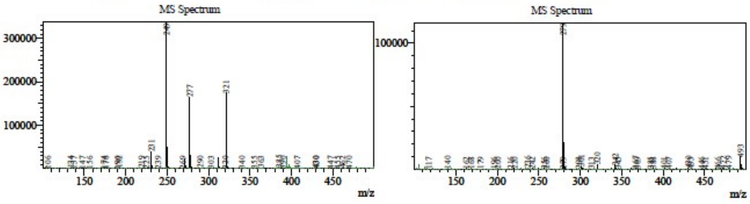

Fig. S80. HPLC-MS data for 6-(4-fluorophenyl)pyridazin-3(2*H*)-one (20a) and 6-(4-fluorophenyl)-2-(2-hydroxypro-pyl)pyridazin-3(2*H*)-one (20b) by Method B:

Sample Information

Acquired by	: System Administrator
Sample Name	: CSA1076_nyers_AT1
Sample ID	:
Tray#	: 1
Vail#	: 1
Injection Volume	: 3 uL
Data Filename	: CSA1076_nyers_AT1.lcd
Method Filename	: A_5_100_1ml_13min.lcm
Batch Filename	:
Report Filename	: Default.lsr
Date Acquired	: 2019.01.15. 8:52:50
Data Processed	: 2019.01.16. 14:50:25

Chromatogram
CSA1076_nyers_AT1 C:\LabSolutions\Data\Project1\DR\CSA4\CSA1076_nyers_AT1.lcd

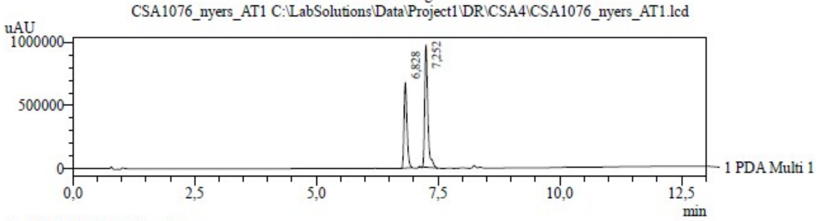

1 PDA Multi 1 / 254nm,4nm

PeakTable C:\LabSolutions\Data\Project1\DR\CSA4\CSA1076_nyers_AT1.lcd

PDA Ch1 254nm

Peak#	Ret. Time	Area	Height	Area %	Height %
1	6,828	2836034	680745	39,726	41,223
2	7,252	4302924	970623	60,274	58,777
Total		7138957	1651369	100,000	100,000

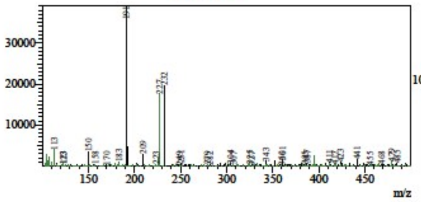

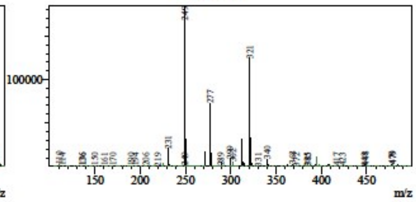

Fig. S81. HPLC-MS data for purified 2-(2-hydroxypropyl)-6-iodopyridazin-3(2*H*)-one (23):

Sample Information

Acquired by	: System Administrator
Sample Name	: CSA1098_nyers_lio_AT1
Sample ID	:
Tray#	: 1
Vail#	: 17
Injection Volume	: 3 uL
Data Filename	: CSA1098_nyers_lio_AT1.lcd
Method Filename	: A_5_100_1ml_13min.lcm
Batch Filename	: 20190318.lcb
Report Filename	: DEFAULT.lsr
Date Acquired	: 2019.03.18. 15:58:06
Data Processed	: 2019.03.20. 10:34:55

Chromatogram

CSA1098_nyers_lio_AT1 C:\LabSolutions\Data\Project1\DR\20190318\CSA1098_nyers_lio_AT1.lcd

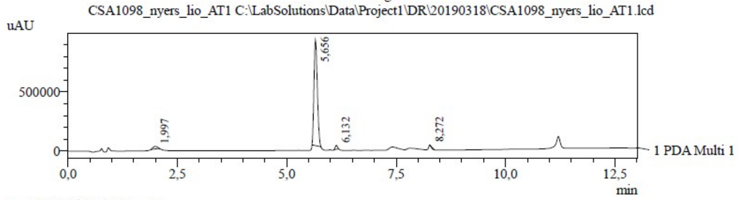

1 PDA Multi 1 / 254nm,4nm

PeakTable C:\LabSolutions\Data\Project1\DR\20190318\CSA1098_nyers_lio_AT1.lcd

PDA Ch1 254nm

Peak#	Ret. Time	Area	Height	Area %	Height %
1	1,997	149724	21446	3,050	2,255
2	5,656	4675302	893386	95,233	93,954
3	6,132	121702	34052	2,479	3,581
4	8,272	-37421	1993	-0,762	0,210
Total		4909306	950877	100,000	100,000

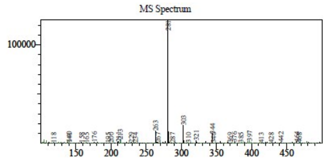

Fig. S82. HPLC-MS data for purified 2-(2-hydroxypropyl)-6-(naphthalene-2-yl)pyridazin-3(2*H*)-one (11b) obtained from 6-(naphthalene-2-yl)pyridazin-3(2*H*)-one (11a):

Sample Information

Acquired by	: System Administrator
Sample Name	: CSA1103_nyers_AT1
Sample ID	:
Tray#	: 1
Vail#	: 20
Injection Volume	: 3 uL
Data Filename	: CSA1103_nyers_AT1.lcd
Method Filename	: A_5_100_1ml_13min.lcm
Batch Filename	: 20190318.lcb
Report Filename	: DEFAULT.lsr
Date Acquired	: 2019.03.18. 16:39:01
Data Processed	: 2019.03.20. 10:45:26

Chromatogram

CSA1103_nyers_AT1 C:\LabSolutions\Data\Project1\DR\20190318\CSA1103_nyers_AT1.lcd

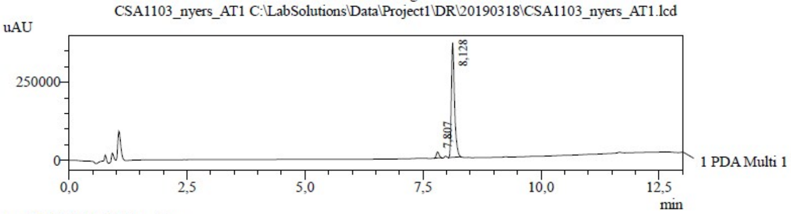

1 PDA Multi 1 / 254nm,4nm

PeakTable C:\LabSolutions\Data\Project1\DR\20190318\CSA1103_nyers_AT1.lcd

PDA Ch1 254nm

Peak#	Ret. Time	Area	Height	Area %	Height %
1	7,807	73545	19090	4,171	4,939
2	8,128	1689792	367462	95,829	95,061
Total		1763337	386552	100,000	100,000

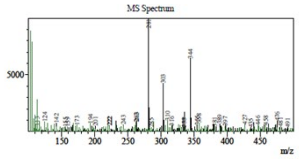

Fig. S83. ^{1}H NMR spectrum of 3,6-diiodopyridazine:

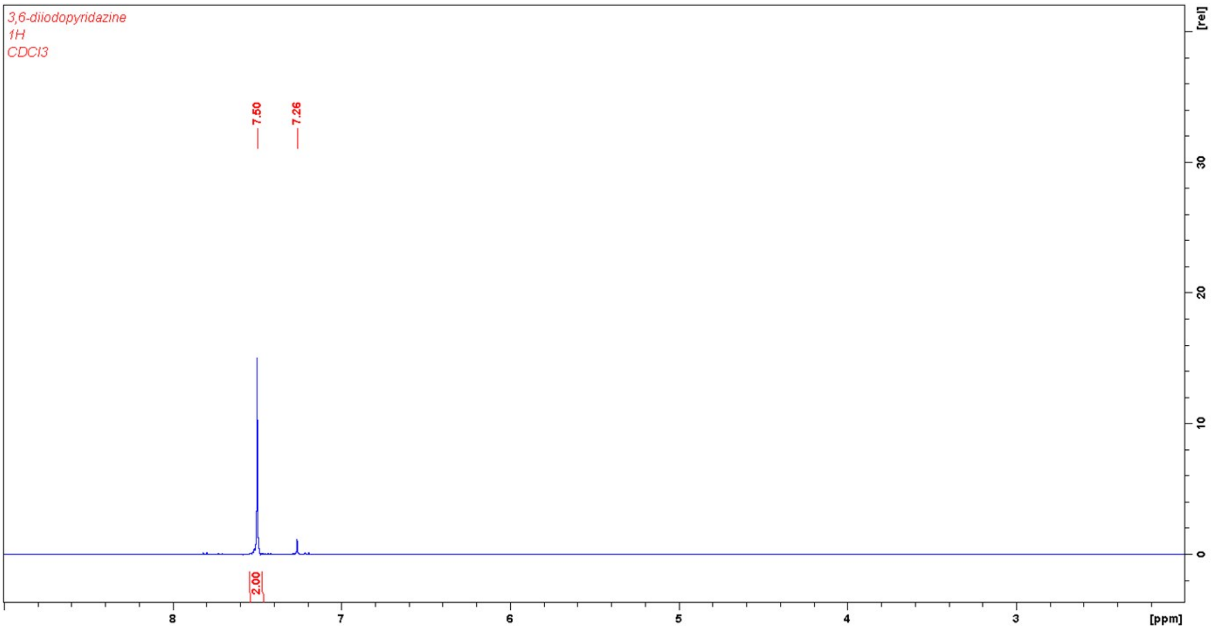

Fig. S84. ^{13}C NMR spectrum of 3,6-diiodopyridazine:

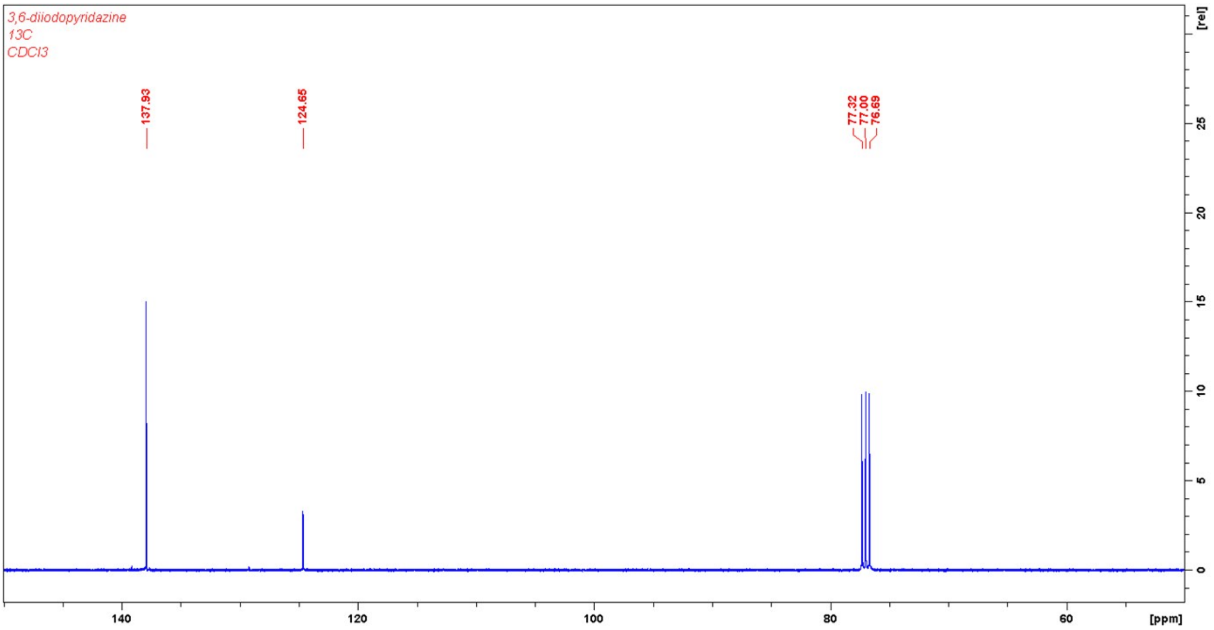

Fig. S85. ^{1}H NMR spectrum of (4): 6-iodopyridazin-3(2*H*)-one

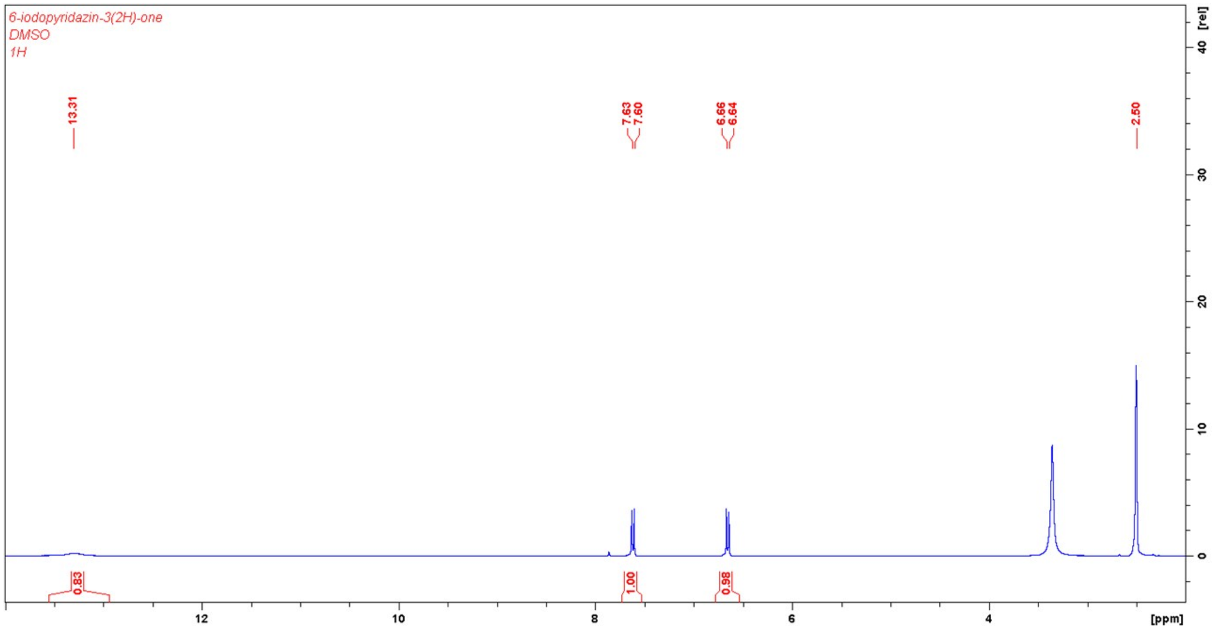

Fig. S86. ^{13}C NMR spectrum of (4): 6-iodopyridazin-3(2*H*)-one

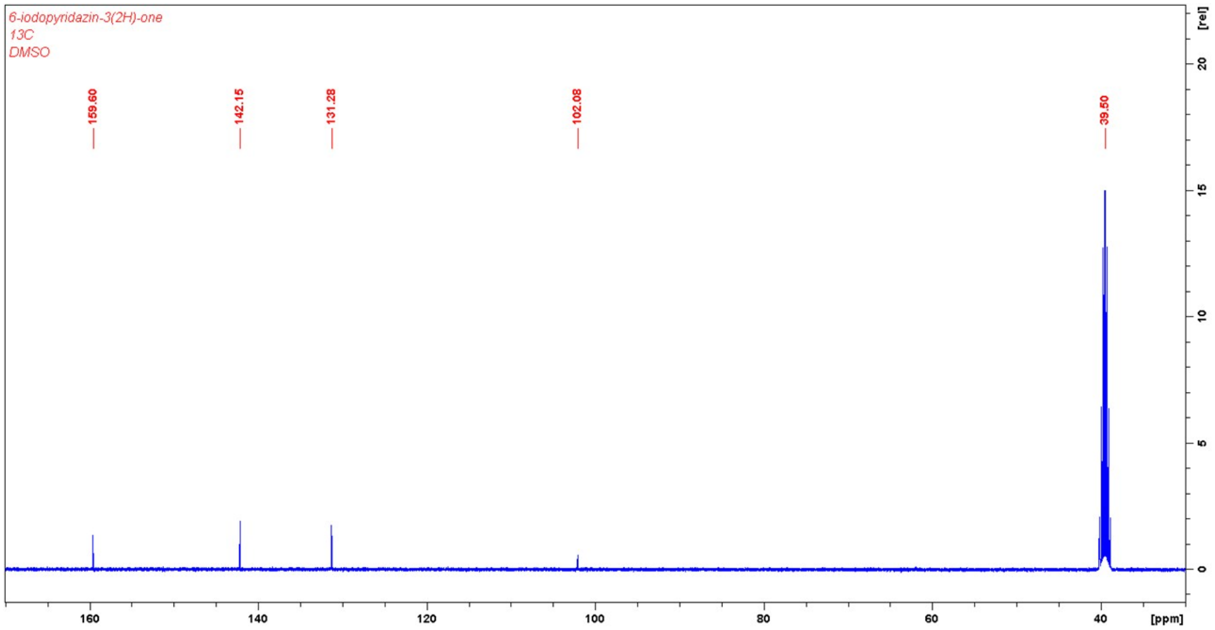

Fig. S87. ^{1}H NMR spectrum of (9): 2-(naphthalen-2-yl)pyridine

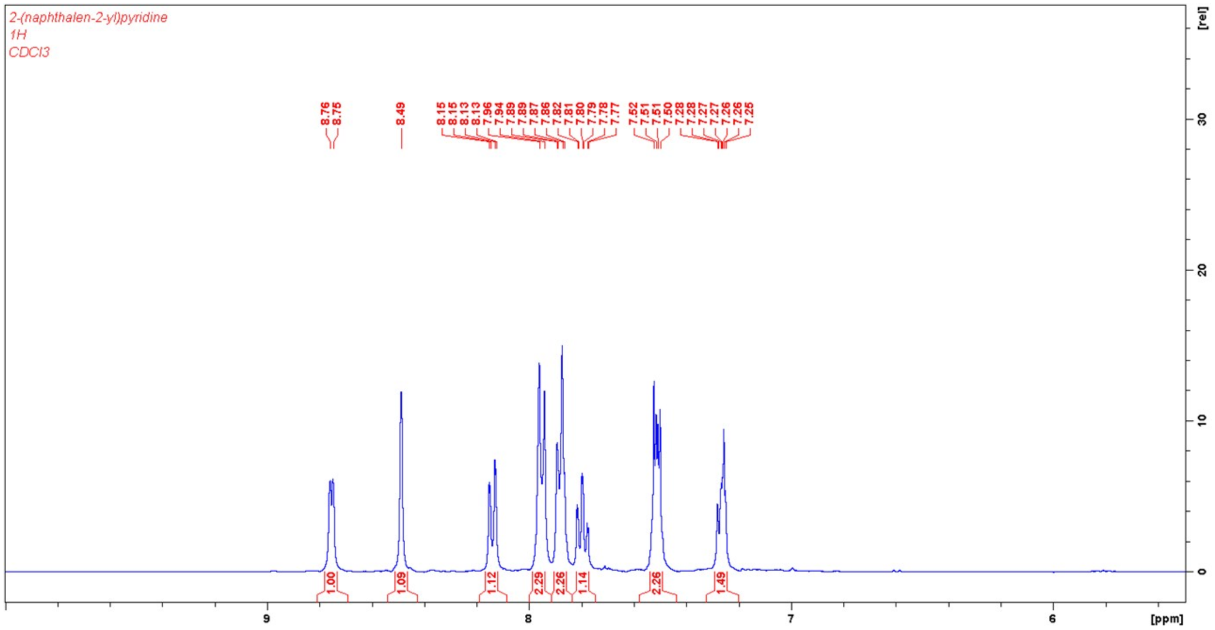

Fig. S88. ^{13}C NMR spectrum of (9): 2-(naphthalen-2-yl)pyridine

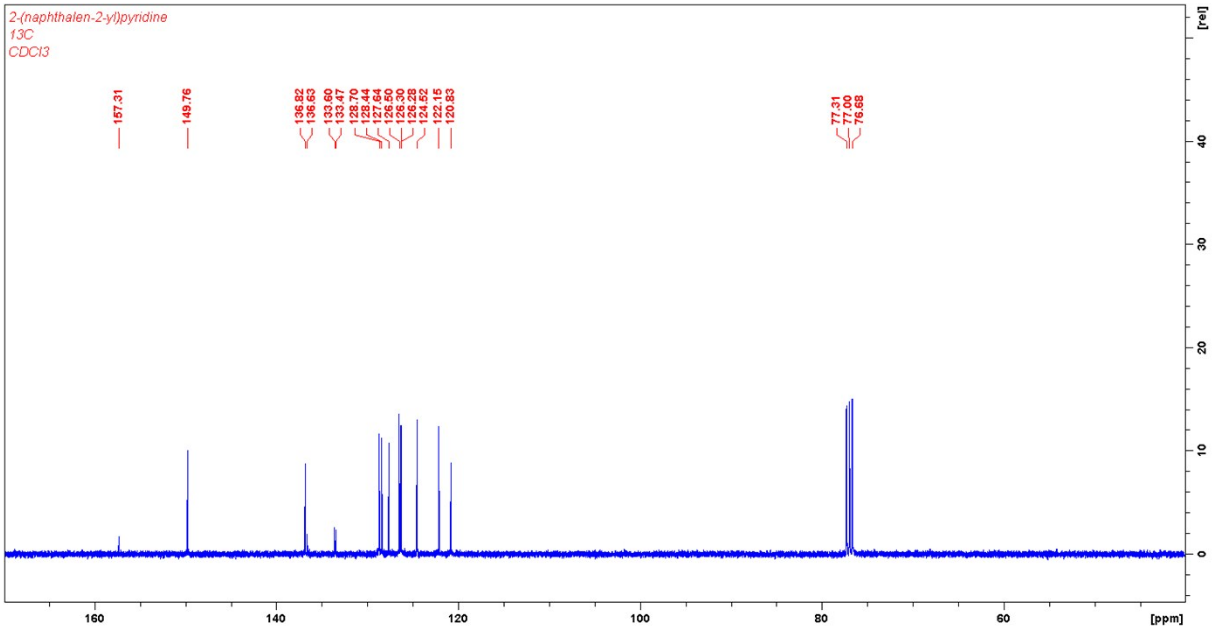

Fig. S89. ^{1}H NMR spectrum of (10): 4-(naphthalen-2-yl)pyridine

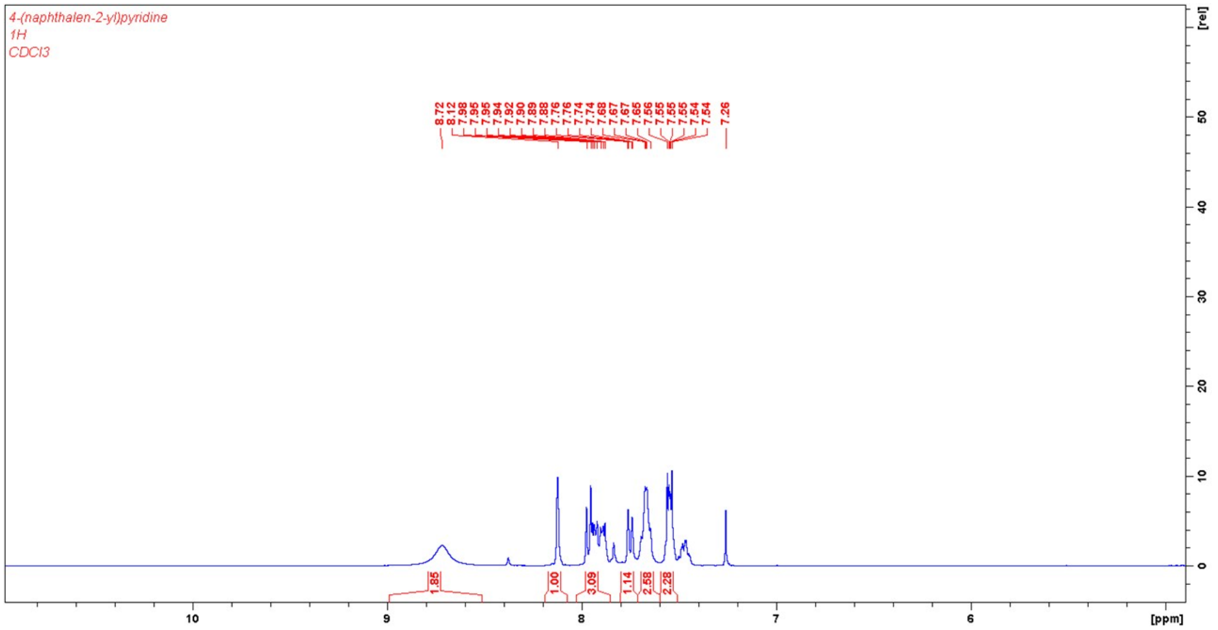

Fig. S90. ^{13}C NMR spectrum of (10): 4-(naphthalen-2-yl)pyridine

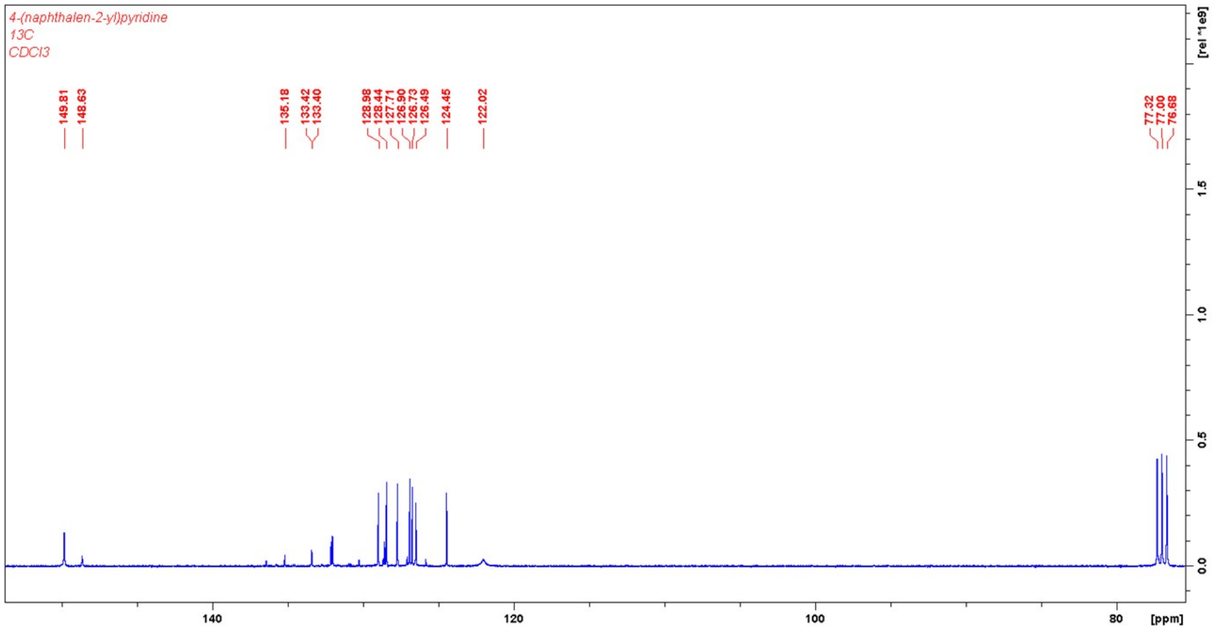

Fig. S91. ^{1}H NMR spectrum of (11a): 6-(naphthalen-2-yl)pyridazin-3(2*H*)-one

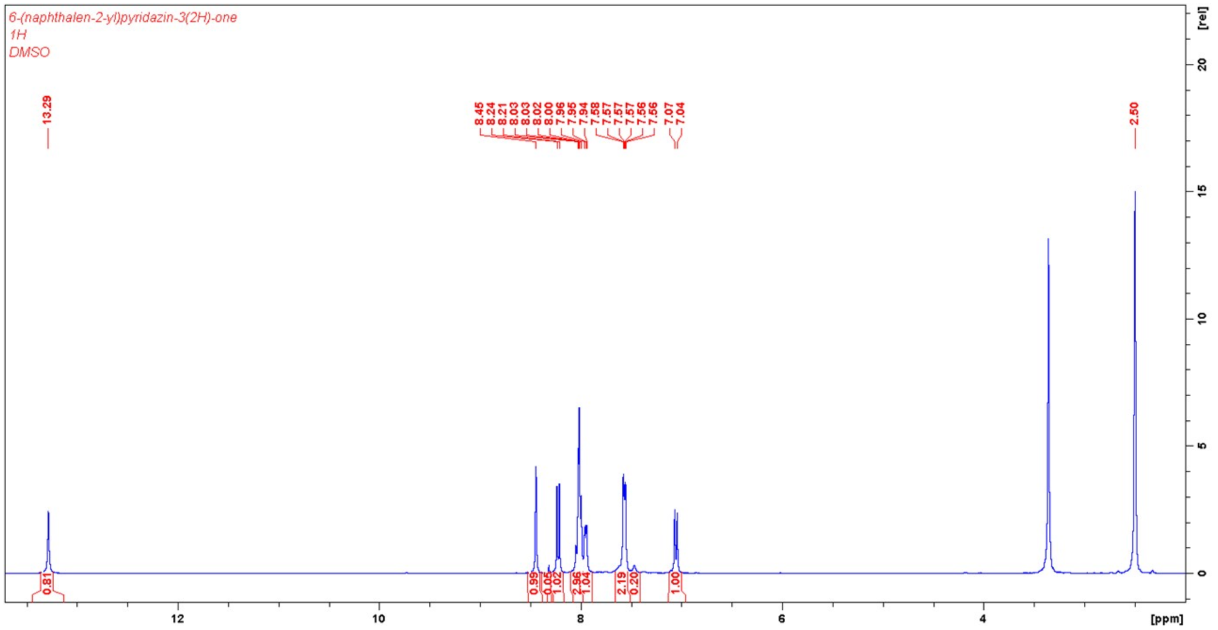

Fig. S92. ^{13}C NMR spectrum of (11a): 6-(naphthalen-2-yl)pyridazin-3(2*H*)-one

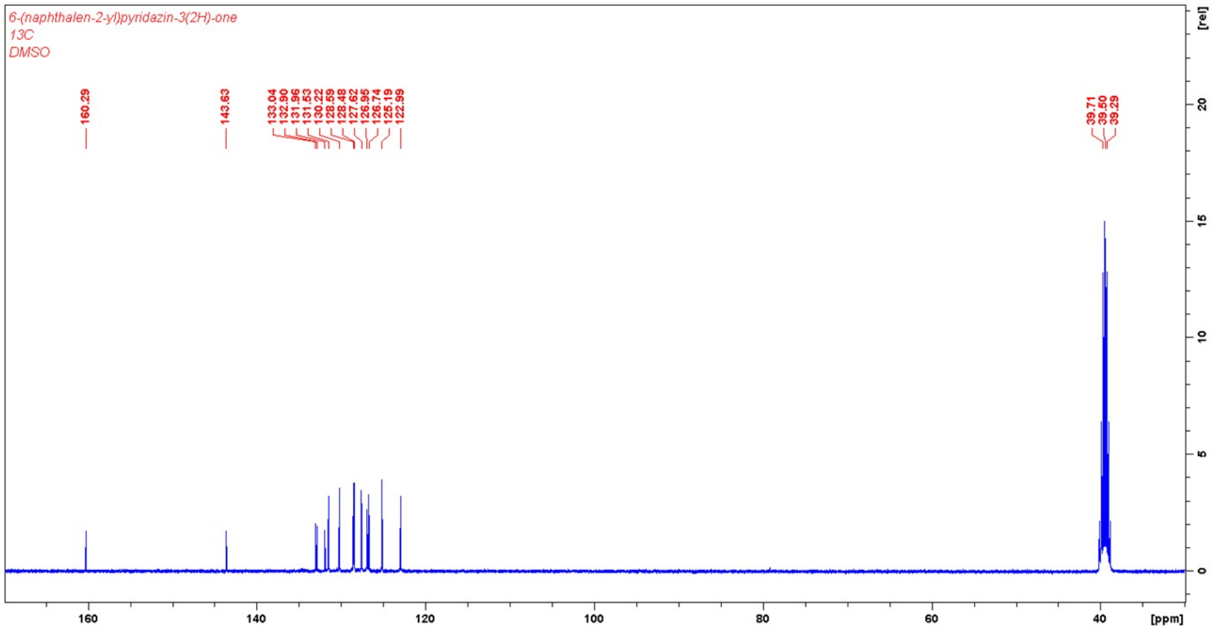

Fig. S93. ^{1}H NMR spectrum of (11b): 2-(2-hydroxypropyl)-6-(naphthalen-2-yl)pyridazin-3(2*H*)-one

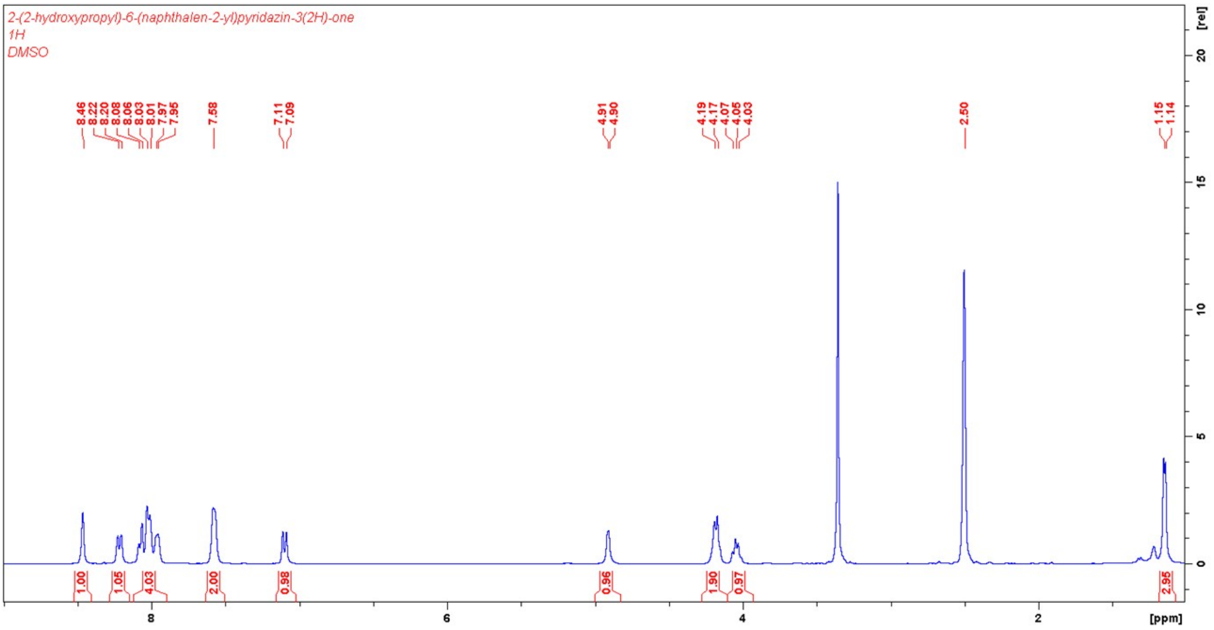

Fig. S94. ^{13}C NMR spectrum of (11b): 2-(2-hydroxypropyl)-6-(naphthalen-2-yl)pyridazin-3(2*H*)-one

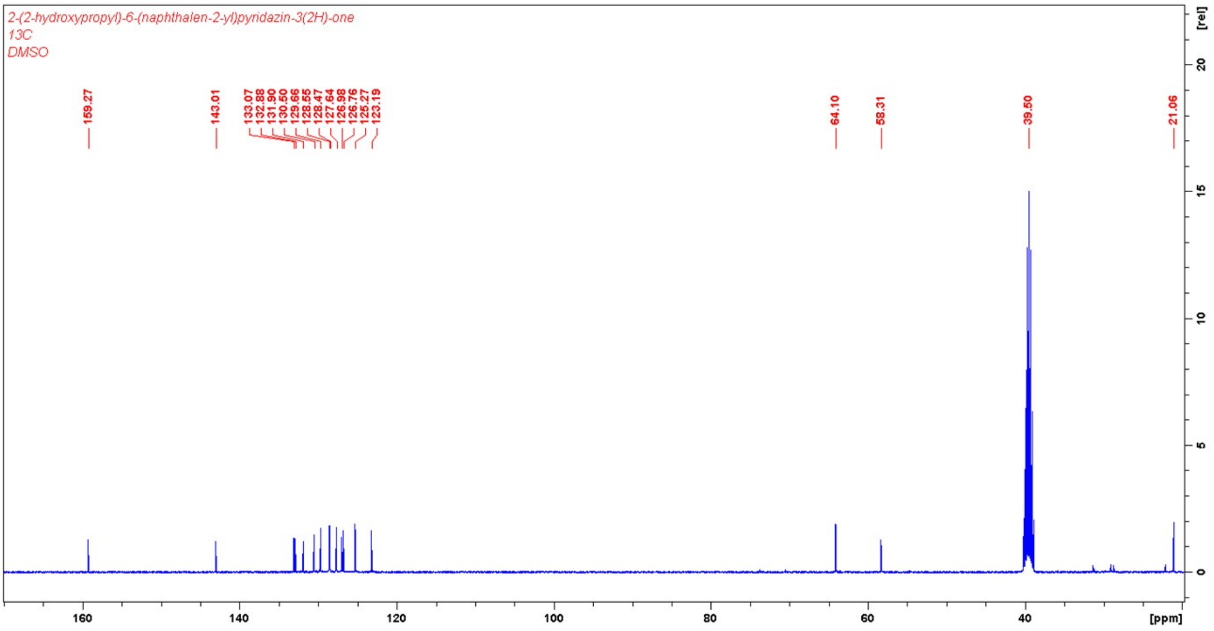

Fig. S95. ^{1}H NMR spectrum of (12): 2-phenylpyridine

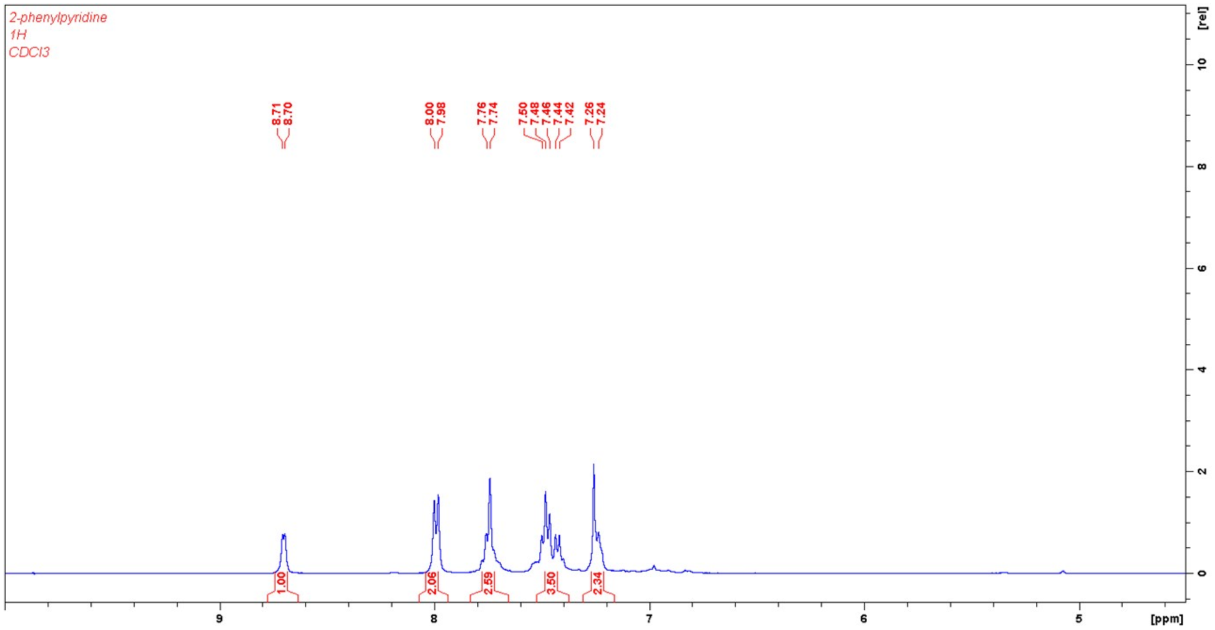

Fig. S96. ^{13}C NMR spectrum of (12): 2-phenylpyridine

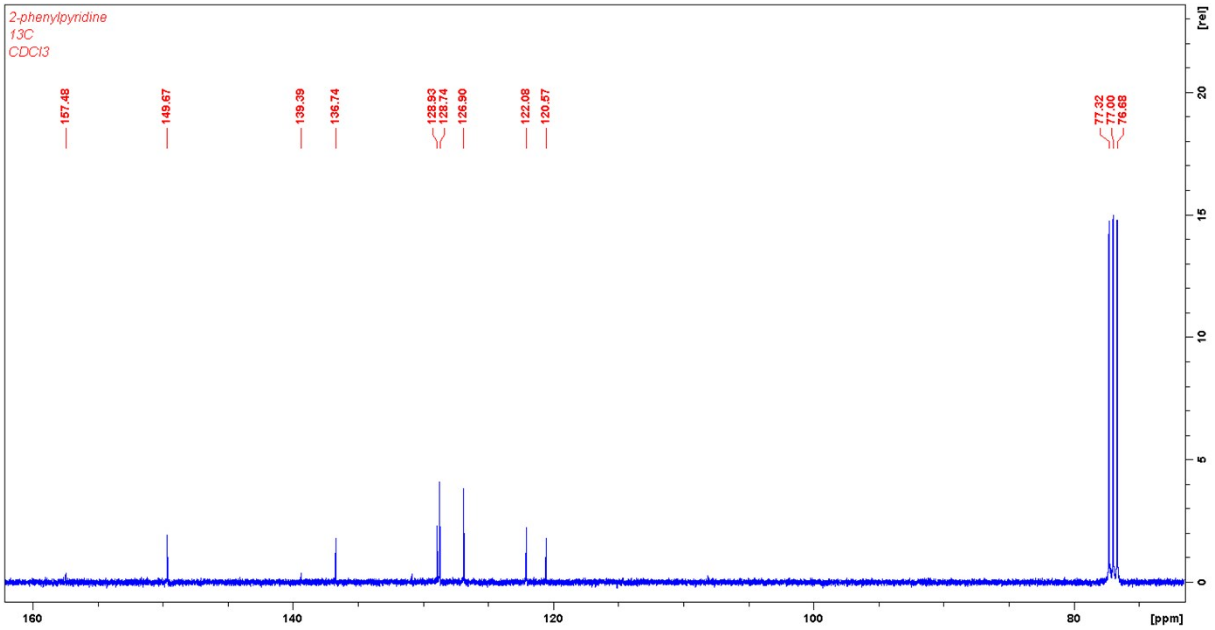

Fig. S97. ^{1}H NMR spectrum of (13): 4-phenylpyridine

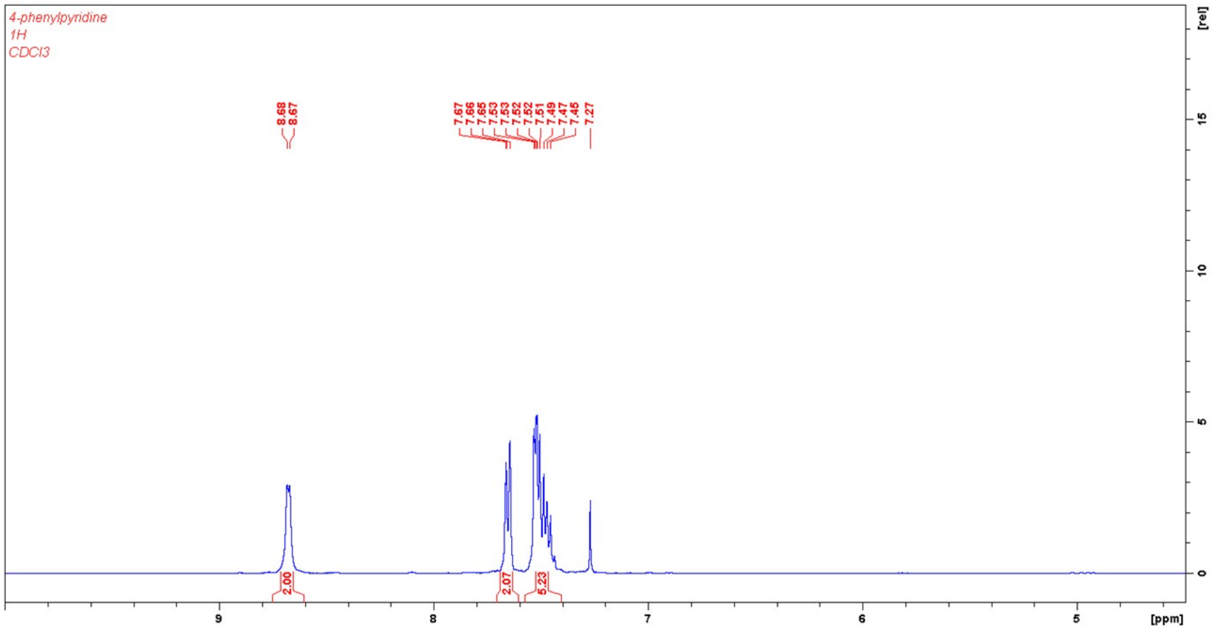

Fig. S98. ^{13}C NMR spectrum of (13): 4-phenylpyridine

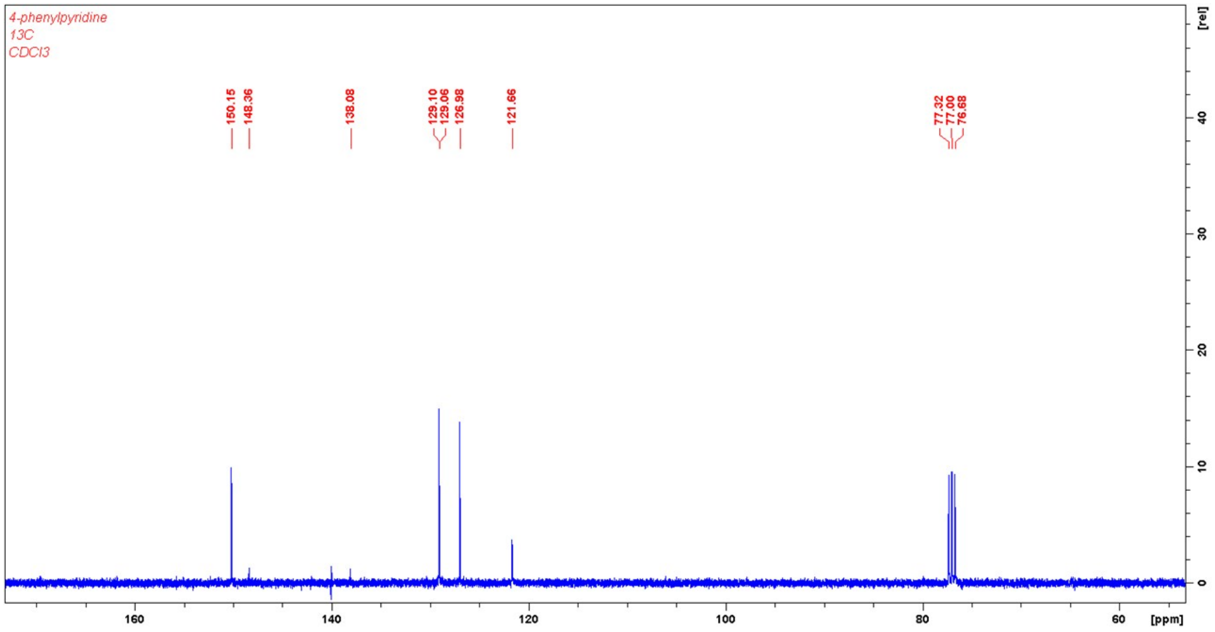

Fig. S99. ^{1}H NMR spectrum of (14): 2-(biphenyl-4-yl)pyridine

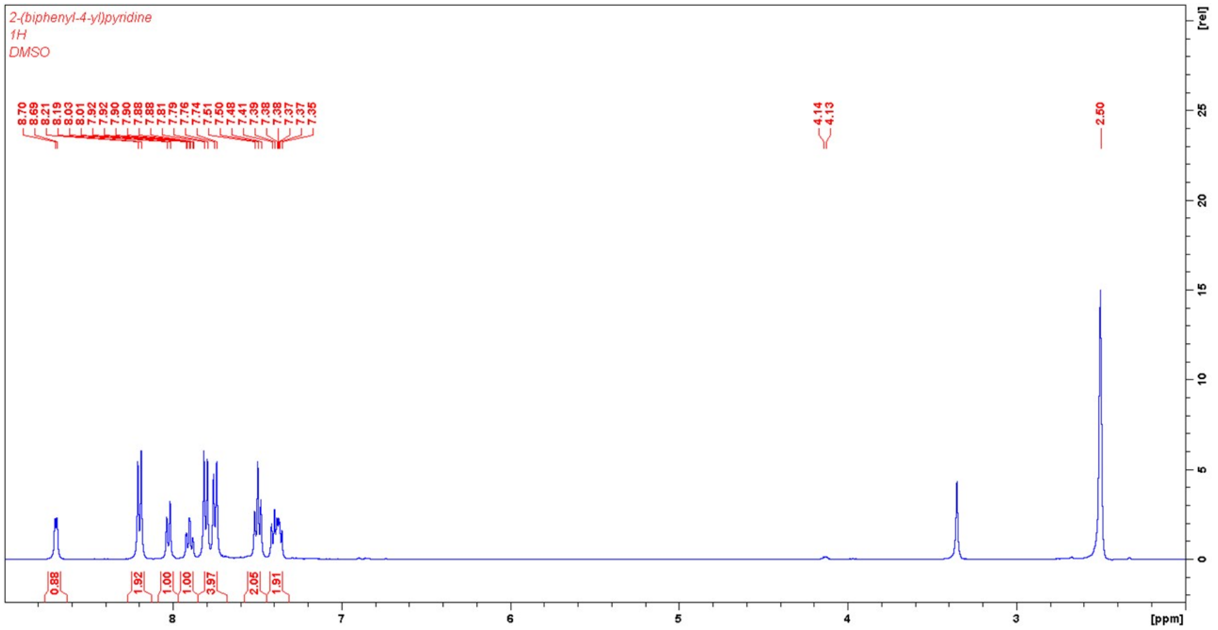

Fig. S100. ^{13}C NMR spectrum of (14): 2-(biphenyl-4-yl)pyridine

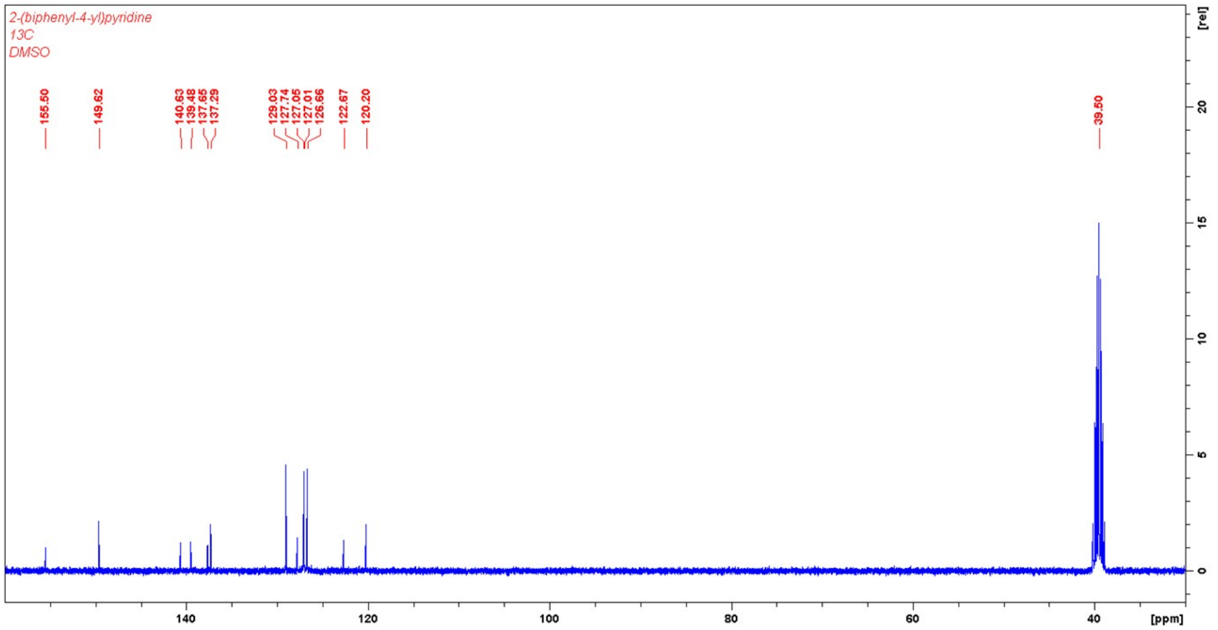

Fig. S101. ^{1}H NMR spectrum of (15): 4-(biphenyl-4-yl)pyridine

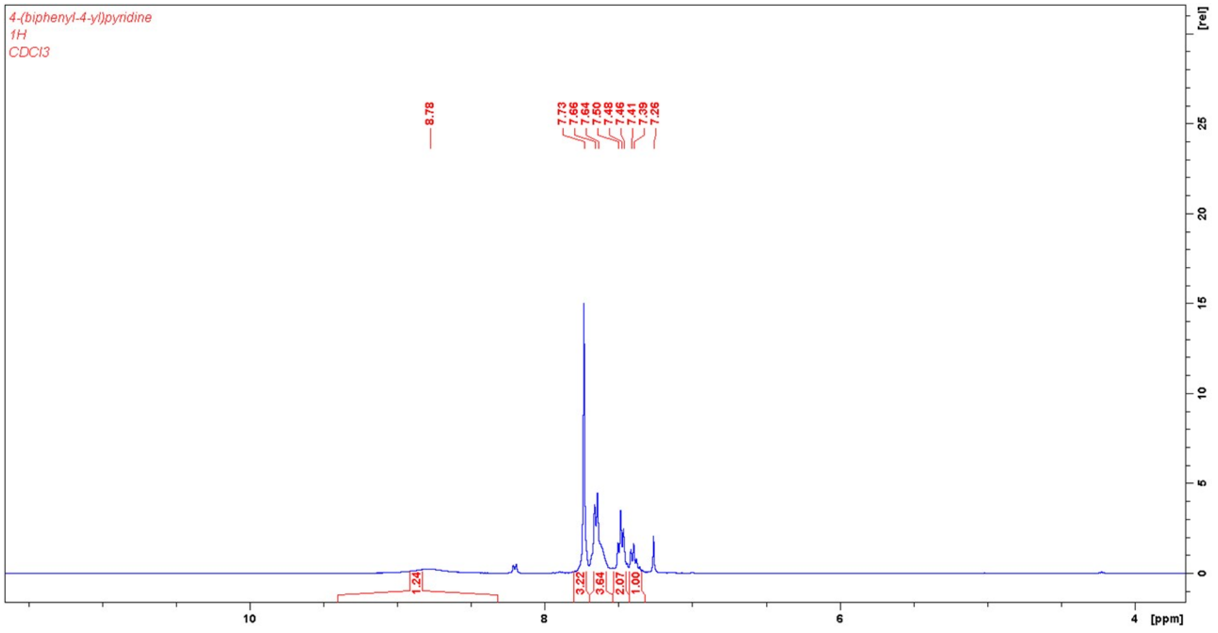

Fig. S102. ^{13}C NMR spectrum of (15): 4-(biphenyl-4-yl)pyridine

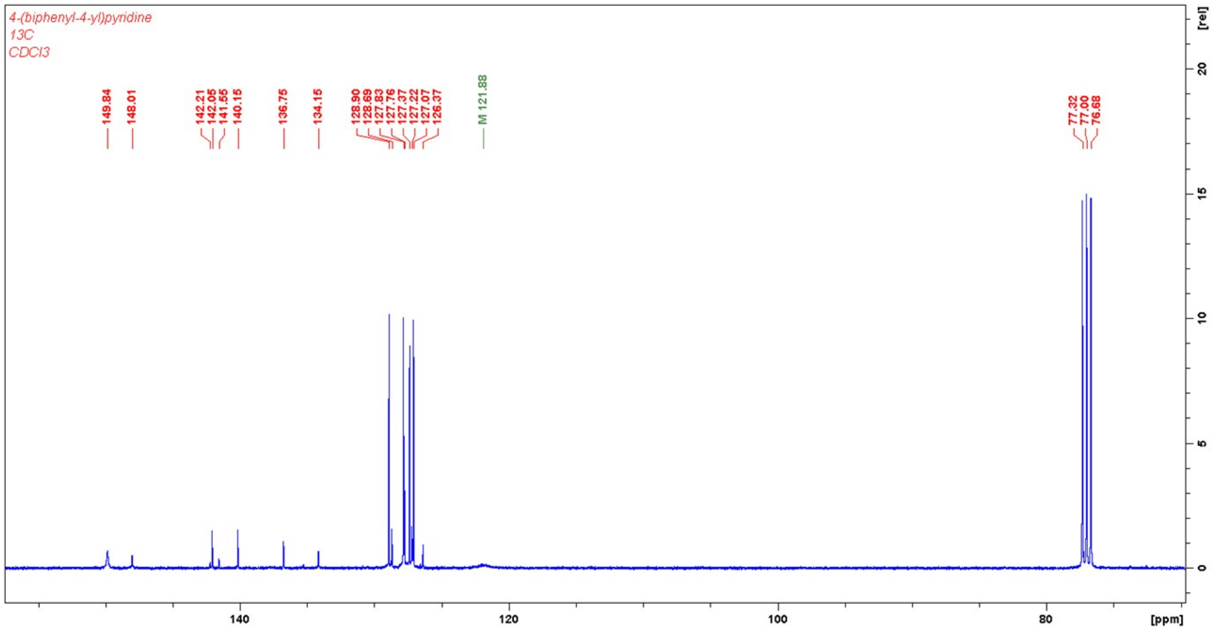

Fig. S103. ^{1}H NMR spectrum of (16): 2-(4-fluorophenyl)pyridine

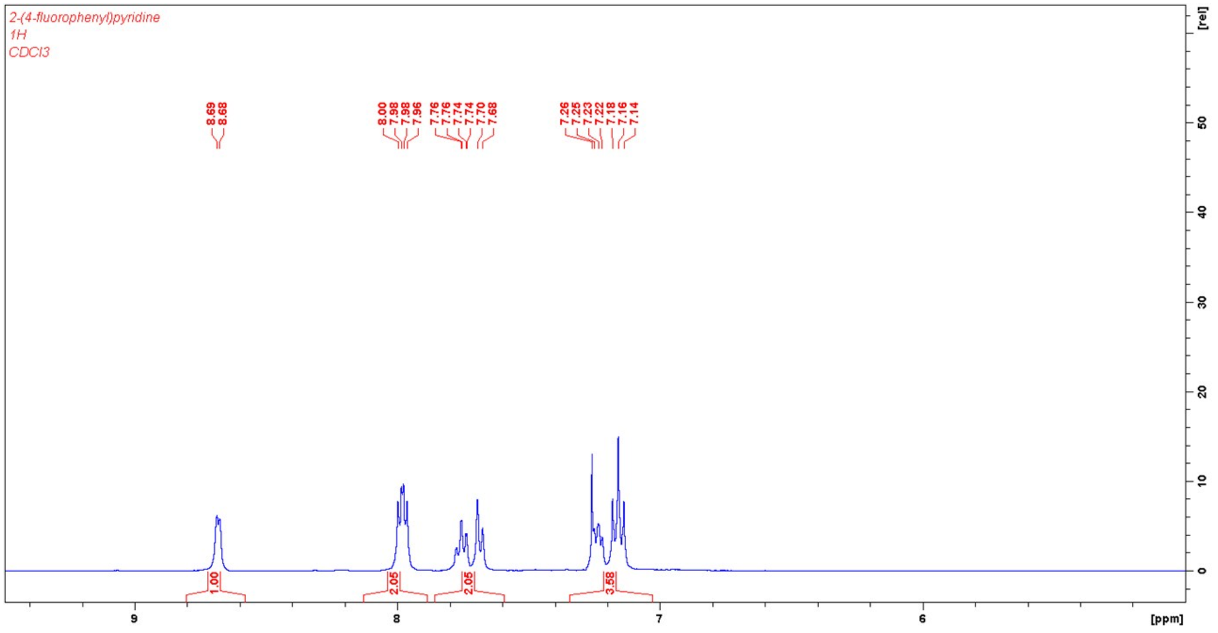

Fig. S104. ^{13}C NMR spectrum of (16): 2-(4-fluorophenyl)pyridine

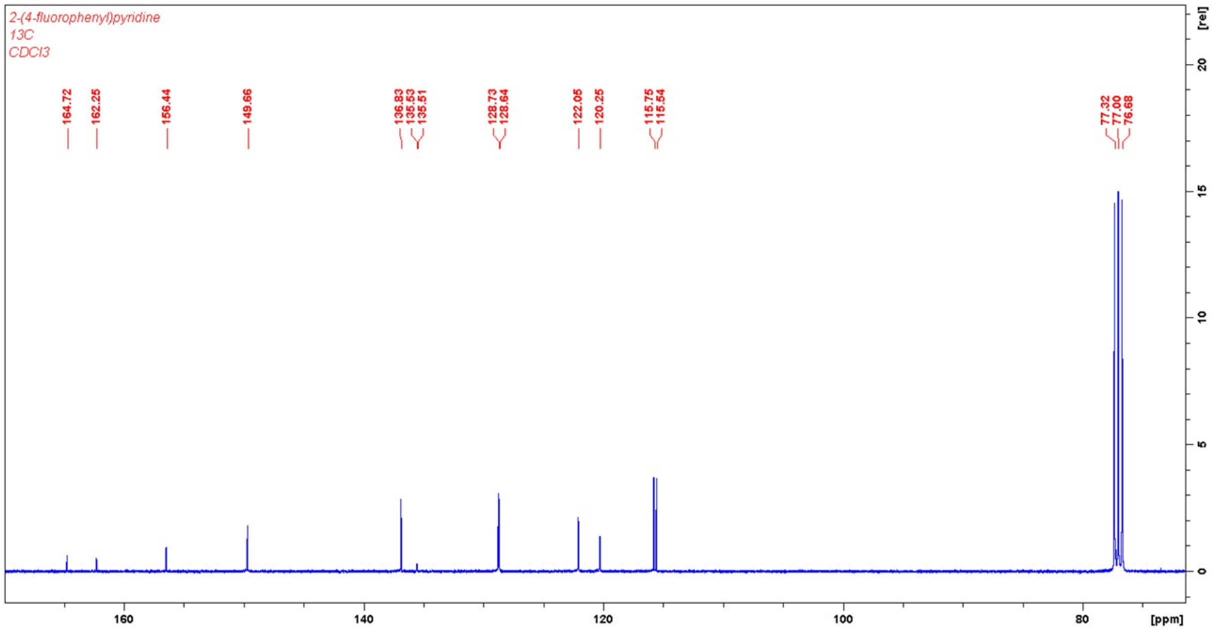

Fig. S105. ^{1}H NMR spectrum of (17): 4-(4-fluorophenyl)pyridine

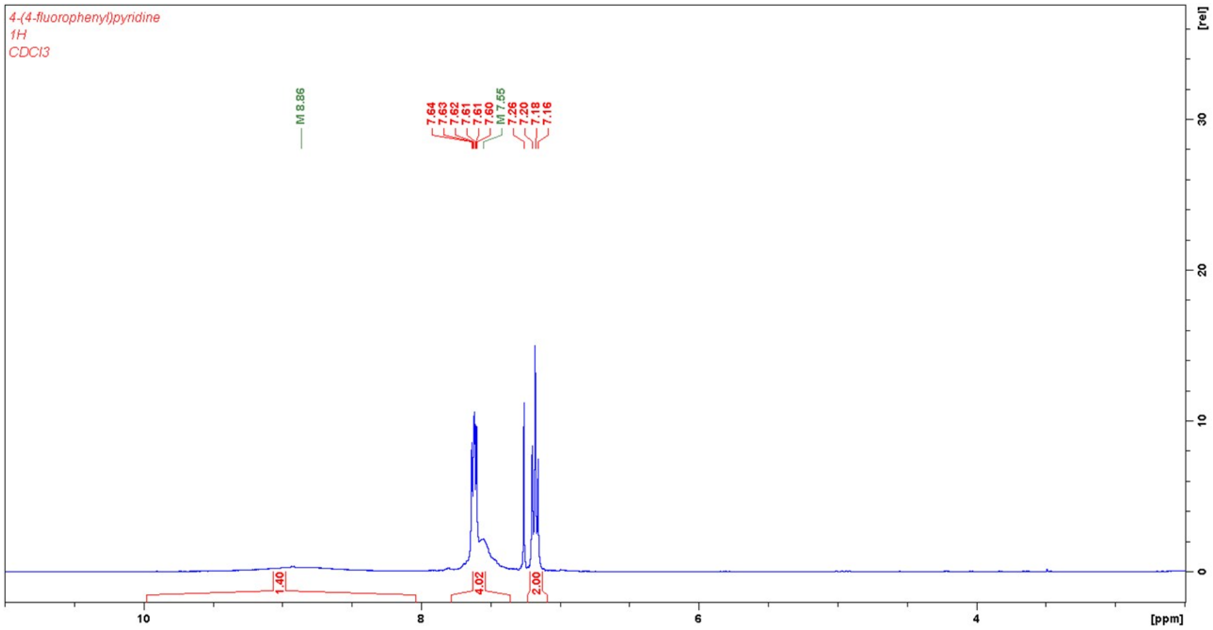

Fig. S106. ^{13}C NMR spectrum of (17): 4-(4-fluorophenyl)pyridine

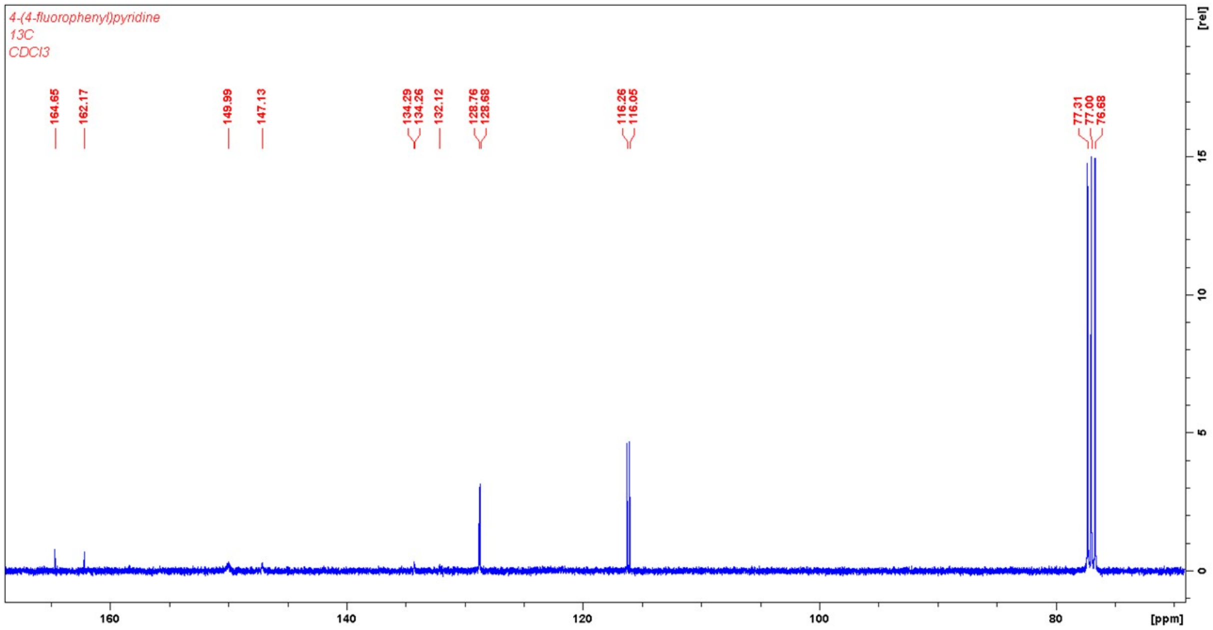

Fig. S107. ^{1}H NMR spectrum of (18a): 6-phenylpyridazin-3(2*H*)-one

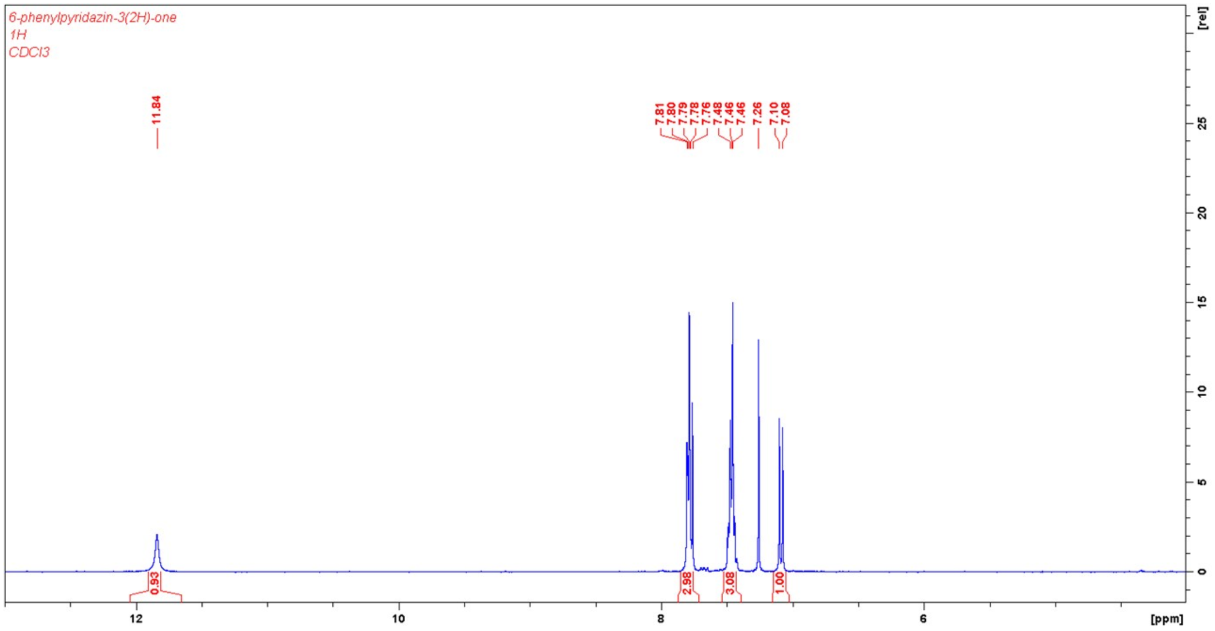

Fig. S108. ^{13}C NMR spectrum of (18a): 6-phenylpyridazin-3(2*H*)-one

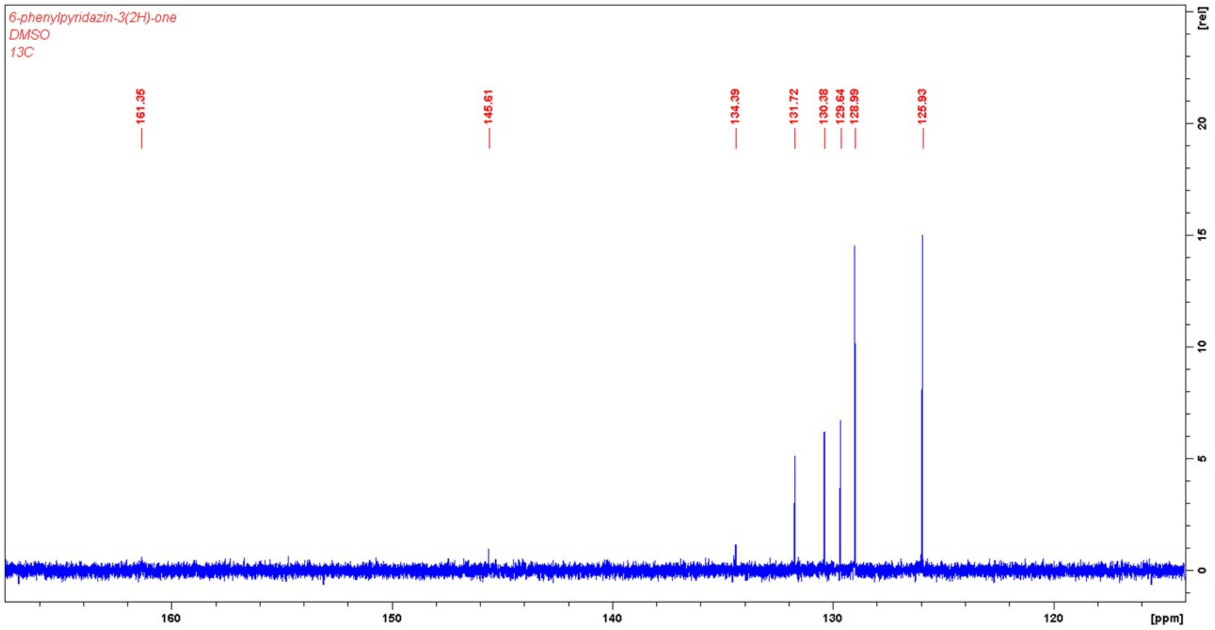

Fig. S109. ^{1}H NMR spectrum of (18b): 2-(2-hydroxypropyl)-6-phenylpyridazin-3(2*H*)-one

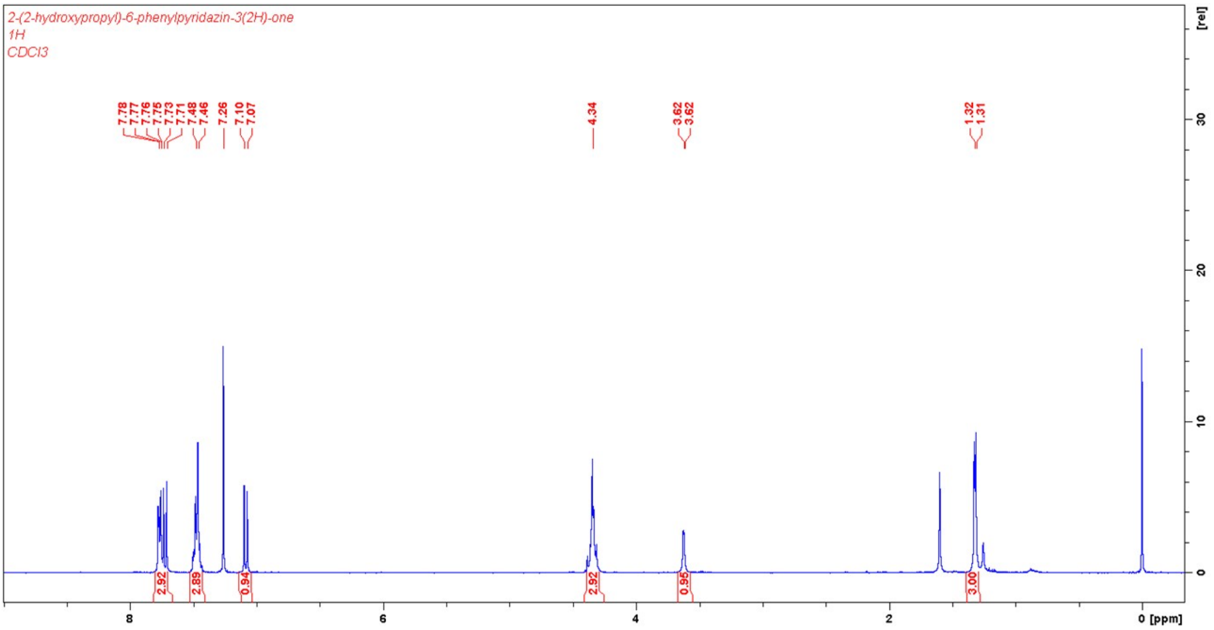

Fig. S110. ^{13}C NMR spectrum of (18b): 2-(2-hydroxypropyl)-6-phenylpyridazin-3(2*H*)-one

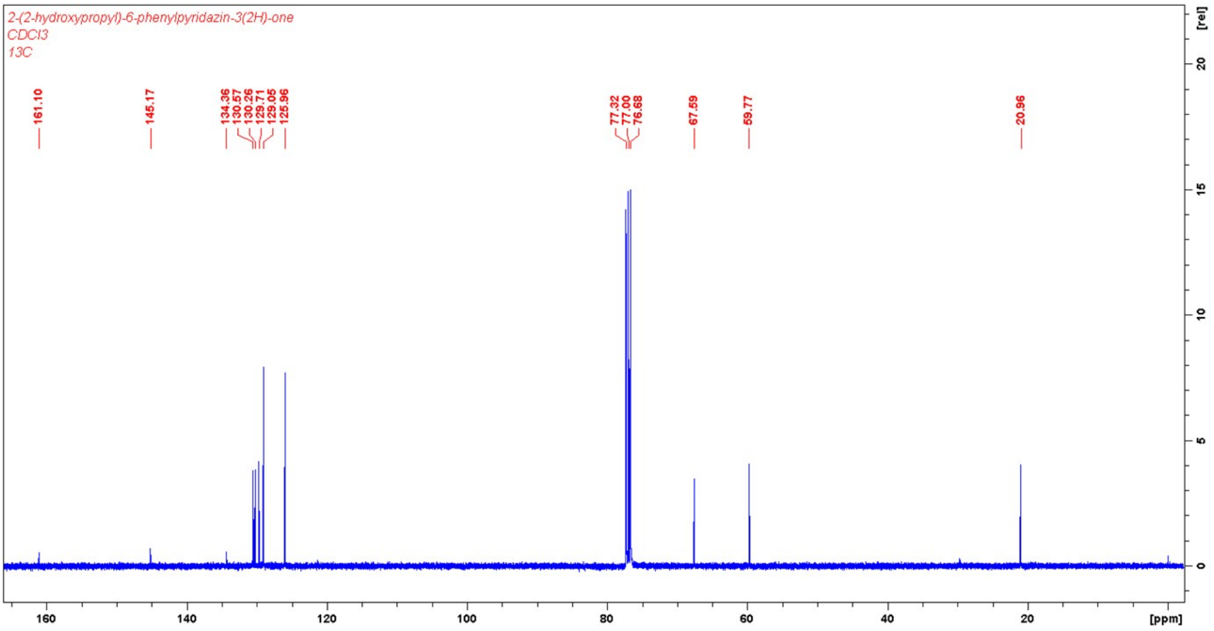

Fig. S111. ^{1}H NMR spectrum of (19a): 6-(biphenyl-4-yl)pyridazin-3(2H)-one

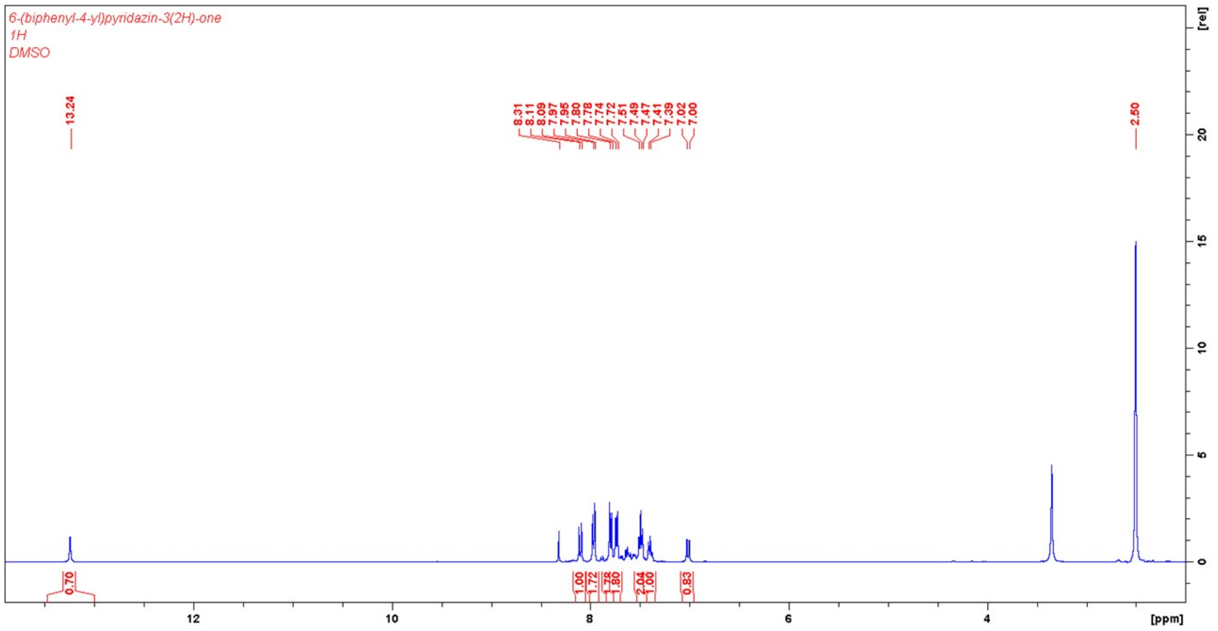

Fig. S112. ^{13}C NMR spectrum of (19a): 6-(biphenyl-4-yl)pyridazin-3(2H)-one

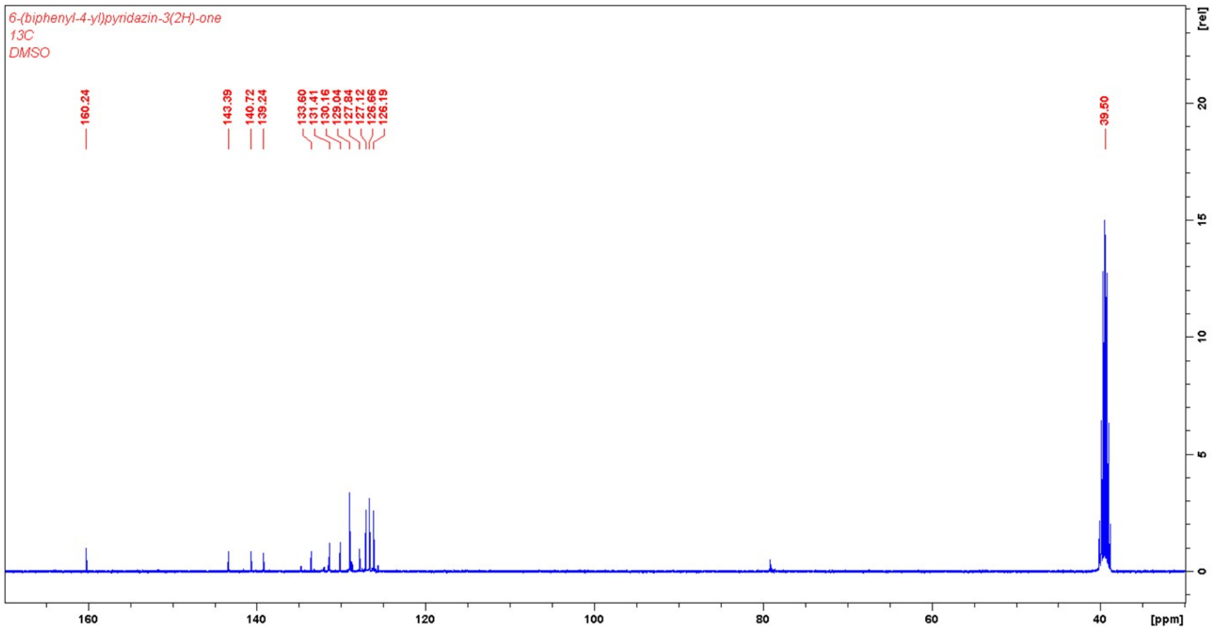

Fig. S113. ^{1}H NMR spectrum of (19b): 6-(biphenyl-4-yl)-2-(2-hydroxypropyl)pyridazin-3(2*H*)-one

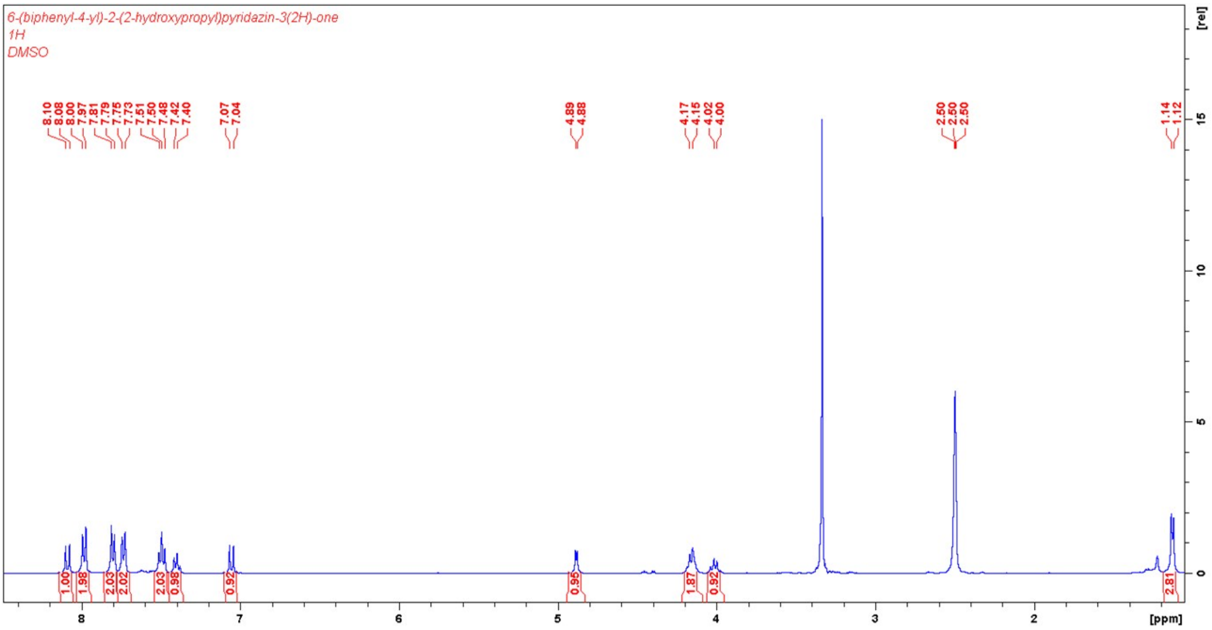

Fig. S114. ^{13}C NMR spectrum of (19b): 6-(biphenyl-4-yl)-2-(2-hydroxypropyl)pyridazin-3(2*H*)-one

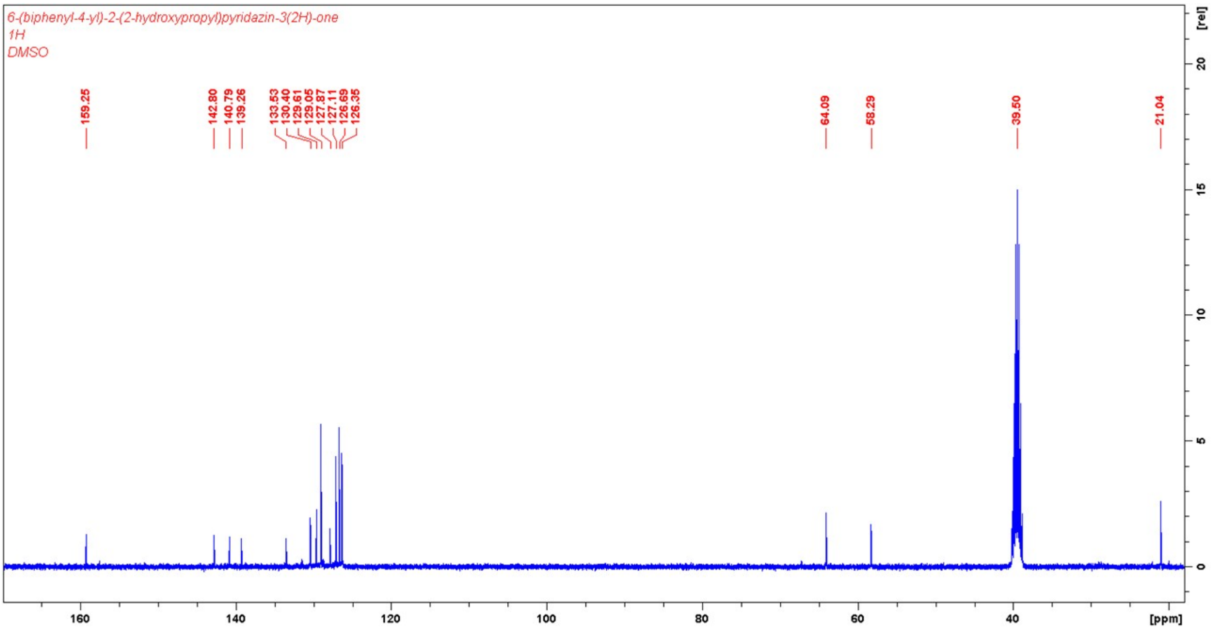

Fig. S115. ^{1}H NMR spectrum of (20a): 6-(4-fluorophenyl)pyridazin-3(*2H*)-one

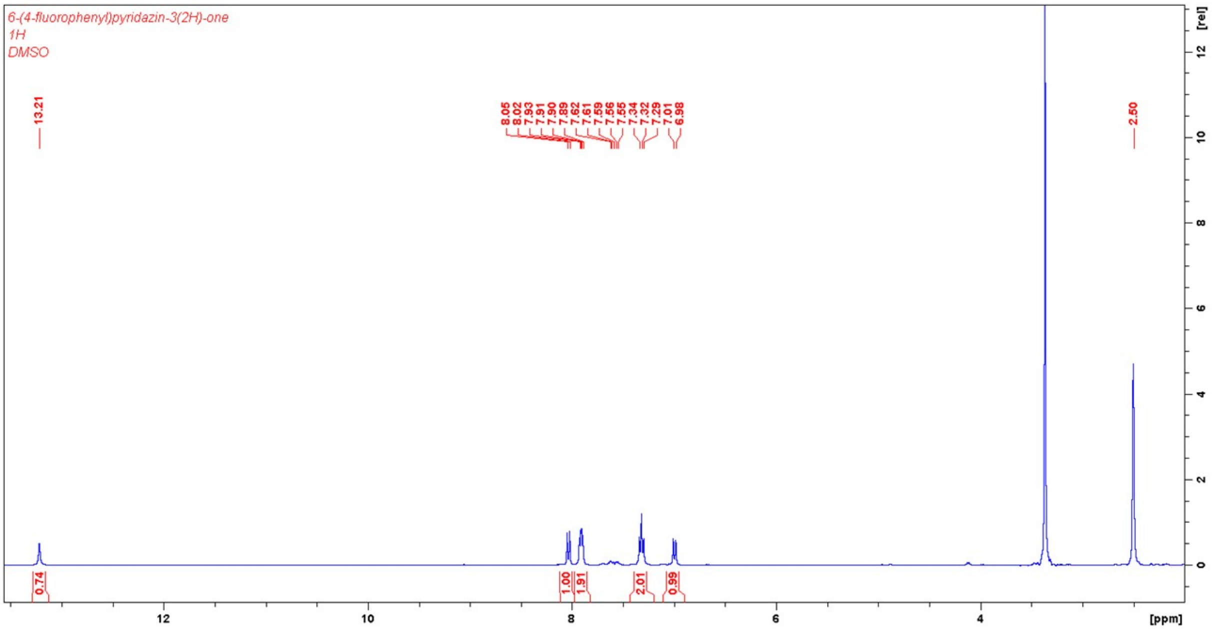

Fig. S116. ^{13}C NMR spectrum of (20a): 6-(4-fluorophenyl)pyridazin-3(*2H*)-one

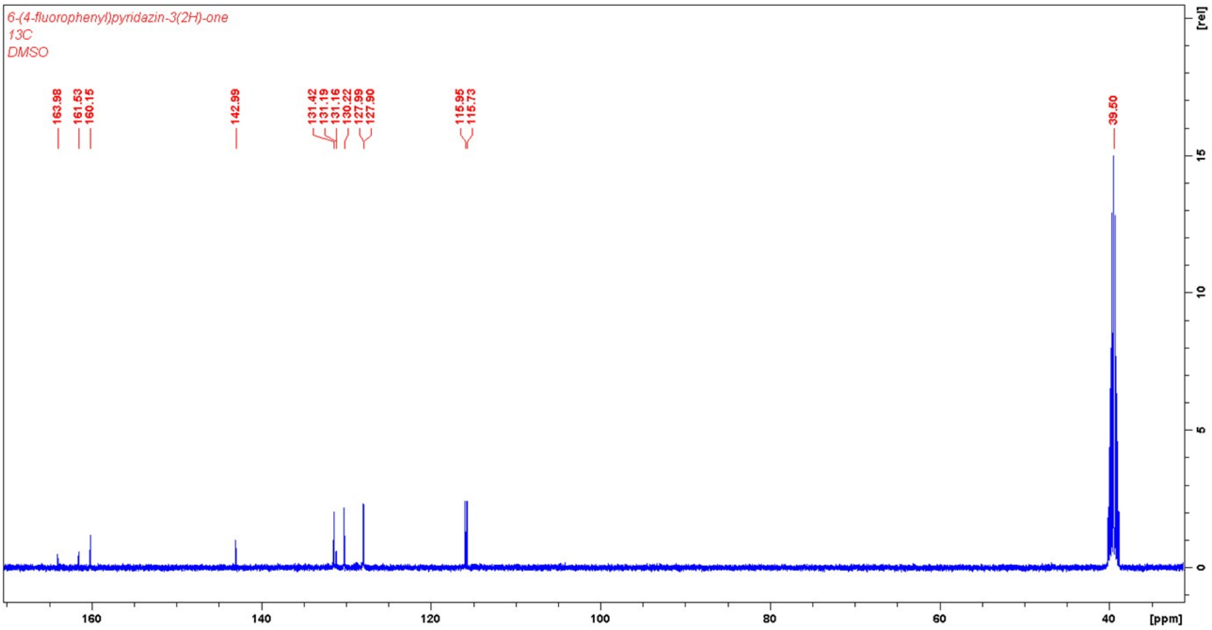

Fig. S117. ^{1}H NMR spectrum of (20b): 6-(4-fluorophenyl)-2-(2-hydroxypropyl)pyridazin-3(*2H*)-one

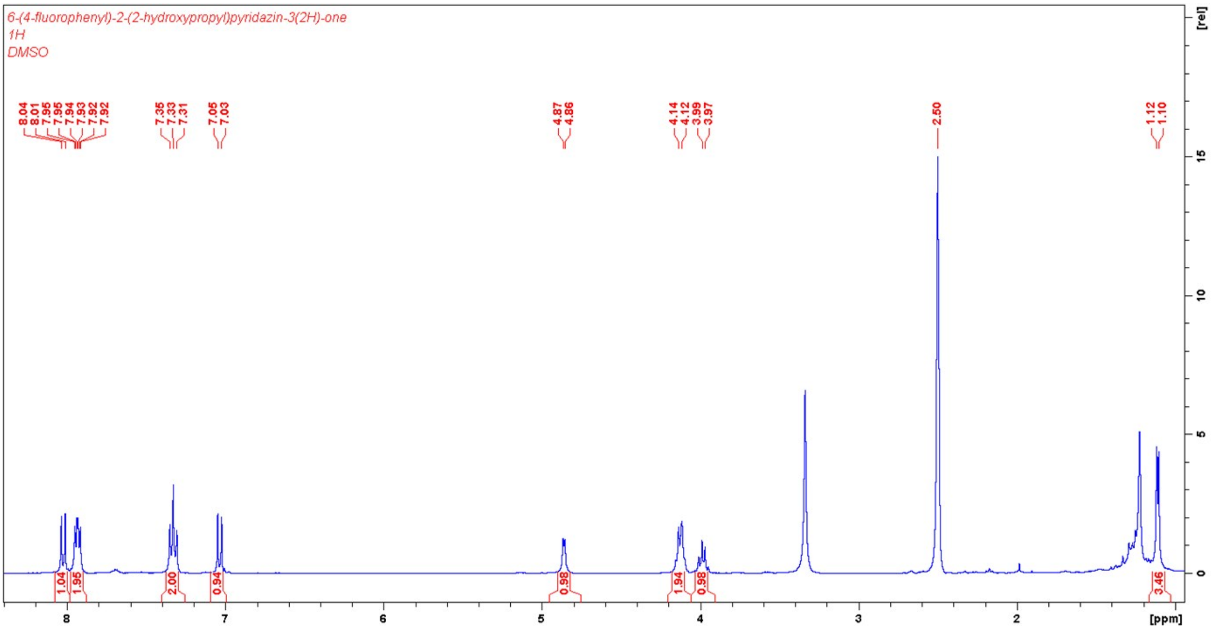

Fig. S118. ^{13}C NMR spectrum of (20b): 6-(4-fluorophenyl)-2-(2-hydroxypropyl)pyridazin-3(*2H*)-one

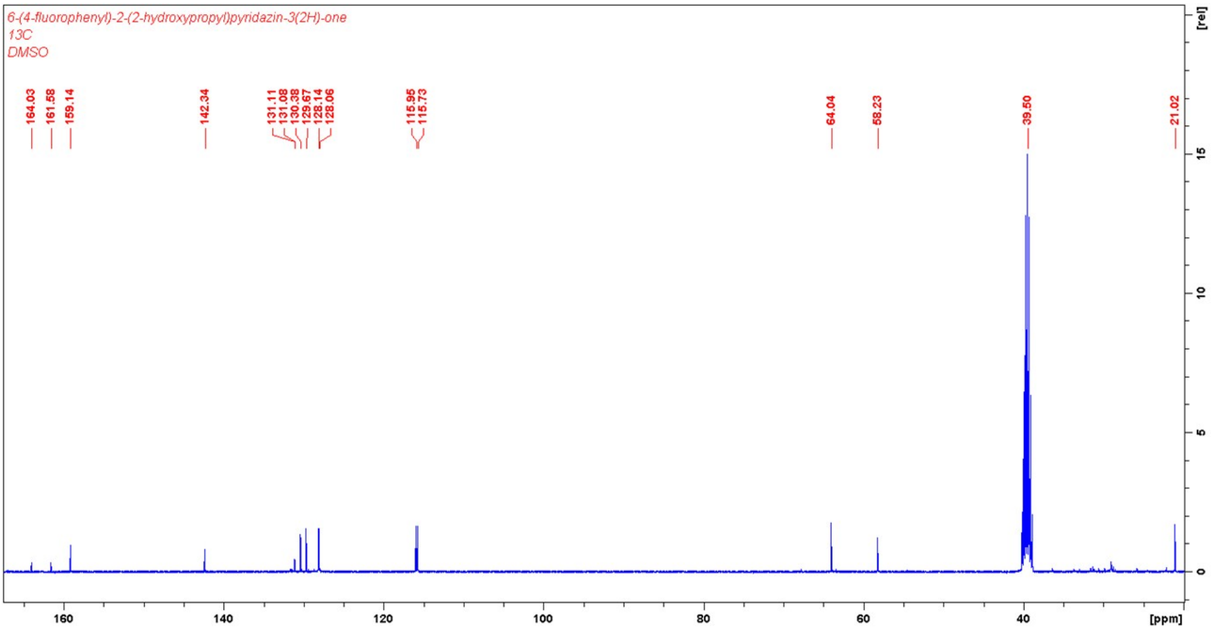

Fig. S119. ^{1}H NMR spectrum of (23): 2-(2-hydroxypropyl)-6-iodopyridazin-3(*2H*)-one

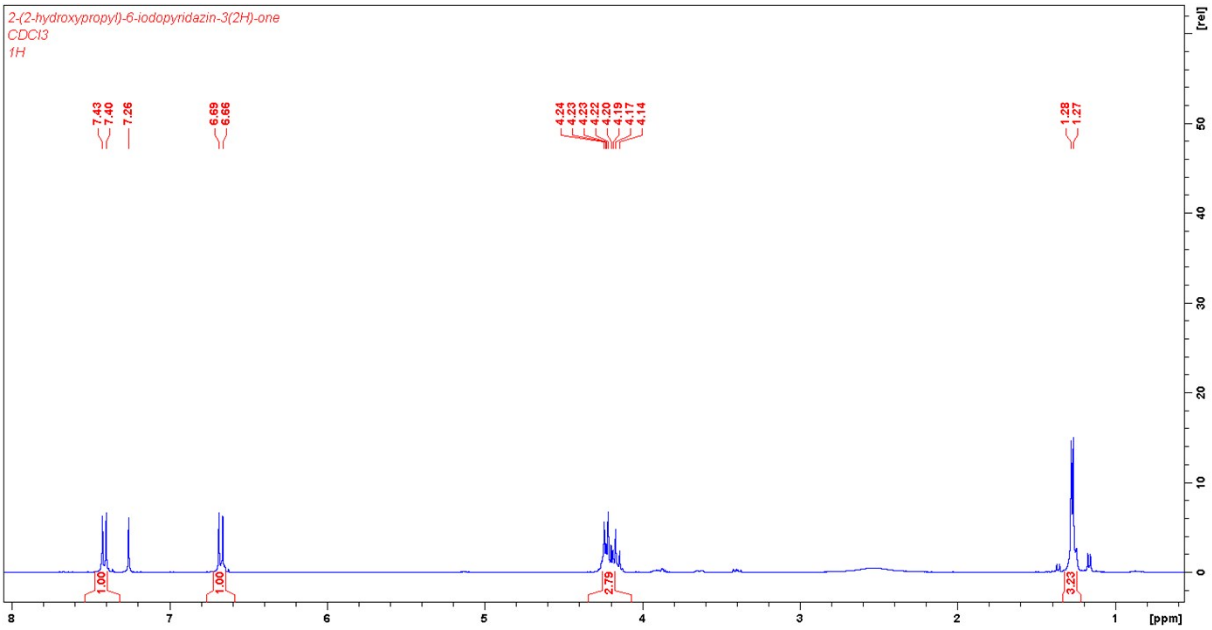

Fig. S120. ^{13}C NMR spectrum of (23): 2-(2-hydroxypropyl)-6-iodopyridazin-3(*2H*)-one

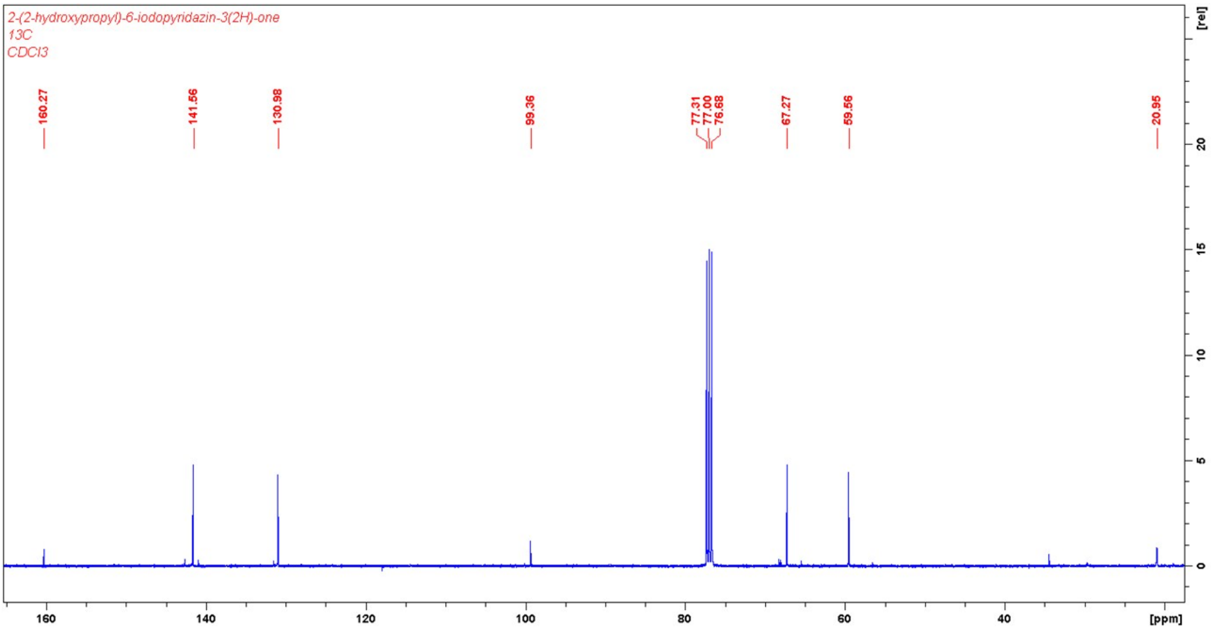

References:

S1 T. Lehmann-Lintz, D. Stenkamp, G. J. Roth, S. G. Mueller, J. Kley, A. Heckel, K. Rudolf, M. Schindler, R. Lotz, P. Tielmann, WO2008071646 (A1), **2008**.

S2 G. J. Roth, S. G. Mueller, T. Lehmann-Lintz, D. Stenkamp, P. Lustenberger, J. Kley, K. Rudolf, A. Heckel, M. Schindler, L. Thomas, R. R. H. Lotz, WO2007048802 (A1), **2007**.

S3 S. Kamiya, A. Nakamura, T. Itai, K. Koshinuma, G. Okusa, *Yakugaku Zasshi* **1966**, *86*, 1099-1104 10.1248/yakushi1947.86.11_1099.

S4 L.-Y. Xi, R.-Y. Zhang, S. Liang, S.-Y. Chen, X.-Q. Yu, *Organic Letters* **2014**, *16*, 5269–5271.

S5 J.-Q. Liu, B.-Y. Hao, H. Zou, W.-H. Zhang, X.-Z. Chen, *Archivoc* **2014**, *5*, 72-93.

S6 V. B. Nazarov, V. G. Avakyan, S. P. Gromov, M. V. Fomina, T. G. Vershinnikova, M. V. Alfimov, *Russian Chemical Bulletin* **2004**, *53*, 2525–2531.

S7 H. Ila, H. Junjappa, *Tetrahedron* **1990**, *46*, 2561–2572.

S8 S. P. Gromov, M. V. Fomina, *Russian Chemical Bulletin* **2004**, *53*, 901-905 10.1023/B:RUCB.0000037861.00184.82.

S9 Q. Gao, Y. Zhu, M. Lian, M. Liu, J. Yuan, G. Yin, A. Wu, *The Journal of Organic Chemistry* **2012**, *77*, 9865–9870.

S10 J. Druey, B. H. Ringier, *Helvetica Chimica Acta* **1951**, *34*, 195–210.

S11 F. Haviv, R. W. DeNet, R. J. Michaels, J. D. Ratajczyk, G. W. Carter, P. R. Young, *Journal of medicinal chemistry* **1983**, *26*, 218–222.

S12 H. Hu, C. Ge, A. Zhang, L. Ding, *Molecules* **2009**, *14*, 3153–3160.

S13 S. M. Ghodse, V. N. Telvekar, *Tetrahedron Letters* **2017**, *58*, 524–526.

S14 M. Tobisu, T. Furukawa, N. Chatani, *Chemistry Letters* **2013**, *42*, 1203–1205.

S15 B. Li, Y. Yu, P. Zhao, S. Zhang, *Chemistry–An Asian Journal* **2016**, *11*, 3550–3556.

S16 G. Ranjani, R. Nagarajan, *Organic Letters* **2017**, *19*, 3974–3977.

S17 Y. Shen, J. Chen, M. Liu, J. Ding, W. Gao, X. Huang, H. Wu, *Chemical Communications* **2014**, *50*, 4292–4295.

S18 T. Funaki, H. Otsuka, N. Onozawa-Komatsuzaki, K. Kasuga, K. Sayama, H. Sugihara, *Journal of Materials Chemistry A* **2014**, *2*, 15945–15951.

S19 O. Kobayashi, D. Uraguchi, T. Yamakawa, *Organic Letters* **2009**, *11*, 2679–2682.

S20 T. Markovic, B. N. Rocke, D. C. Blakemore, V. Mascitti, M. C. Willis, *Chemical science* **2017**, *8*, 4437–4442.

S21 L. Ackermann, H. K. Potukuchi, A. R. Kapdi, C. Schulzke, *Chemistry - A European Journal* **2010**, *16*, 3300-3303 10.1002/chem.201000032.

S22 A. R. Mazzotti, M. G. Campbell, P. Tang, J. M. Murphy, T. Ritter, *Journal of the American Chemical Society* **2013**, *135*, 14012-14015 10.1021/ja405919z.

S23 A. Jakab, Z. Dalicsek, T. Holczbauer, A. Hamza, I. Pápai, Z. Finta, G. Timári, T. Soós, *European Journal of Organic Chemistry* **2015**, *2015*, 60–66.

S24 O. P. Shkurko, S. G. Baram, V. P. Mamaev, *Chemistry of Heterocyclic Compounds* **1983**, *19*, 60-65 10.1007/BF00512817.

S25 B. Li, Z. Zhang, *Journal of the Iranian Chemical Society* **2016**, *13*, 637-644 10.1007/s13738-015-0775-9.

S26 K. Kubota, Y. Watanabe, K. Hayama, H. Ito, *Journal of the American Chemical Society* **2016**, *138*, 4338-4341 10.1021/jacs.6b01375.

S27 E. Sotelo, N. Fraiz, M. Yáñez, V. Terrades, R. Laguna, E. Cano, E. Raviña, *Bioorganic & Medicinal Chemistry* **2002**, *10*, 2873–2882.

S28 R. Salives, G. Dupas, N. Plé, G. Quéguiner, A. Turck, P. George, M. Sevrin, J. Frost, A. Almario, A. Li, *Journal of Combinatorial Chemistry* **2005**, *7*, 414–420.

S29 P. D. Reich-Rohrwig, R. D. Woerther, R. D. Schoenbeck, E. Kloimstein, DE2445681A1, **1976**.

S30 R. D. Schonbeck, E. I. Kloimstein, R. D. Worther, P. D. Reich-Rohrwig, AT333774B, **1976**.

S31 R. Ross, S. H. Shaber, US5763440A, **1998**.

S32 J. G. R. Allen, J. J. W. Hanifin, D. B. Moran, J. D. Albright, DE2741763A1, **1978**.

S33 J. D. Albright, D. B. Moran, W. B. Wright Jr, J. B. Collins, B. Beer, A. S. Lippa, E. N. Greenblatt, *Journal of Medicinal Chemistry* **1981**, *24*, 592–600.
S34 F. Csende, Z. Szabo, G. Bernath, G. Stajer, *Synthesis* **1995**, *10*, 1240–1242.
